# Physical activity and health-related quality of life in former elite and recreational cricketers from the UK with upper extremity or lower extremity persistent joint pain: a cross-sectional study

**DOI:** 10.1136/bmjopen-2019-032606

**Published:** 2019-11-11

**Authors:** Garrett Scott Bullock, Gary Collins, Nicholas Peirce, Nigel K Arden, Stephanie R Filbay

**Affiliations:** 1 Nuffield Department of Orthopaedics, Rheumatology and Musculoskeletal Sciences, University of Oxford, Oxford, UK; 2 Arthritis Research UK Centre for Sport, Exercise and Osteoarthritis, Nuffield Department of Orthopaedics, Rheumatology and Musculoskeletal Sciences, University of Oxford, Oxford, UK; 3 Centre for Statistics in Medicine, University of Oxford, Oxford, UK; 4 Centre For Sports Medicine, Nottingham University Hospitals Trust, Nottingham, UK; 5 National Cricket Performance Centre, England and Wales Cricket Board, Loughborough, UK

**Keywords:** shoulder, knee, ankle, hip, hand, sport, retired athletes

## Abstract

**Objective:**

To evaluate and compare physical activity (PA) and health-related quality of life (HRQoL) in former elite and recreational cricketers with upper extremity (UE), lower extremity (LE) or no joint pain.

**Study design:**

Cross-sectional cohort.

**Setting:**

Despite the high prevalence of joint pain in former athletes, the impact of UE pain and LE pain on PA and HRQoL and potential differences between former recreational and elite athletes are poorly understood.

**Participants:**

703 former cricketers aged ≥18 years (mean age 58.7, SD 12.9, played an average of 30 (IQR 20–40) seasons, 72% of whom had played at a recreational level) were recruited through the Cricket Health and Wellbeing Study and met eligibility requirements (UE pain, LE pain or no joint pain (defined as pain on most days of the past month)).

**Primary and secondary outcomes:**

The International Physical Activity Questionnaire-Short Form collected weekly metabolic equivalents (METS), while the Short-Form 8 collected physical (PCS) and mental (MCS) component scores. Kruskal-Wallis tests with Dunn’s post-hoc and multivariable linear regressions were performed.

**Results:**

Weekly METS were similar in former cricketers with UE pain (median (IQR) 2560 (722–4398)), LE pain (2215 (527–3903)) and no pain (2449 (695–4203), p=0.39). MCS were similar between groups (UE pain 56.0 (52.1–60.0); LE pain 55.2 (51.1–59.4); no pain 54.7 (50.7–58.7), p=0.38). PCS were more impaired in former cricketers with UE pain (49.8 (44.9–54.8)) or LE pain (46.7 (41.0–51.9)) compared with no pain (54.2 (51.5–56.9), p<0.0001). Former cricketers with LE pain reported worse PCS than those with UE pain (p=0.04). Similar relationships were observed in former elite and recreational cricketers.

**Conclusion:**

Despite impaired physical components of HRQoL in former cricketers with UE pain or LE pain, pain was not related to PA levels or mental components of HRQoL. Physical components of HRQoL were most impaired in those with LE pain, and findings were similar among former elite and recreational cricketers.

**Strengths and limitations of this study**

- This study took into account the non-linear relationships between different continuous variables (ie, by using fractional polynomials), where most studies assume linearity.
- Missing data were low, allowing for low bias in a complete case analysis.
- Participants with persistent pain in both the upper and lower extremities and participants with back pain were excluded from the analyses, decreasing the generalisability of these findings.
- The literature on Short-Form 8 minimal detectable difference and minimal clinically important difference is sparse and does not evaluate athletic populations, decreasing the interpretability of these data.

## Introduction

Upper and lower extremity osteoarthritis (OA) has a high incidence^1 2^ and a substantial personal and societal burden.^3 4^ Post-traumatic OA occurs at a younger age, with a longer period of disability and less effective treatments than idiopathic OA.^5–7^ Former sport participants have increased post-traumatic OA prevalence compared with the general population,^5 8^ which is associated with persistent joint pain.^9^ Persistent joint pain is the major cause of patients seeking medical attention for OA,^9^ and has been shown to influence a person’s ability to participate in desired forms of activity,^10 11^ which may result in reduced physical activity (PA) levels and negatively impact health-related quality of life (HRQoL).^10–13^

To date, most research investigating OA and pain has focused on the lower extremity.^10–14^ Former professional soccer players were found to have a high prevalence of lower extremity OA and pain compared with controls,^10 11^ while former female recreational athletes were found to have two to three times increased hip and knee OA risk compared with population-matched controls.^14^ Lower extremity OA and pain in former sport participants are associated with impaired function and mobility, increased levels of anxiety and depression, and reduced HRQoL compared with the general population.^10 12–14^

Upper extremity pain can have different barriers to PA participation compared with lower extremity pain.^15–18^ For example, lower extremity pain may have a greater effect on jumping, stair use, walking and running.^19 20^ In contrast, upper extremity pain can have more effect on specific functional activities such as dressing and eating,^15^ or work-related tasks such as carrying and handling objects,^16^ with potential negative impacts on HRQoL.^15^ Upper extremity pain has been related to reduced PA and function, impaired HRQoL, stress and disability in workers.^21–25^ Despite a high prevalence of upper extremity pain in former throwing-sport participants,^26–28^ the impact of upper extremity pain on PA levels and HRQoL in former athletes is poorly understood.

Psychological characteristics associated with successful high-level athletic performance include resilience, mental toughness and advanced coping skills.^29–32^ Greater resilience and coping strategies can have positive impact on PA levels^33–35^ and HRQoL.^34 36–38^ Thus, former elite athletes may possess psychological characteristics that enable them to better cope with chronic pain compared with former recreational sport participants, and this could positively impact PA levels and HRQoL. However, this has not yet been investigated, highlighting the need for further research. It is also not clear if PA levels and HRQoL differ in former sport participants with and without persistent joint pain. The rationale for investigating these relationships in former cricketers was largely due to the high prevalence of upper and lower extremity OA and persistent pain among former cricketers,^12 39^ and our previous qualitative work highlighting that a subgroup of former cricketers experience high quality of life and maintain physically active lifestyles despite persistent joint pain.^40 41^ Therefore, the purposes of this study were the following:

- To evaluate the relationship between persistent joint pain (upper extremity, lower extremity or no persistent joint pain), PA levels and HRQoL in former cricketers.
- To compare these relationships in former elite and recreational cricketer subgroups.

## Methods

### Patient and public involvement

Findings from two qualitative studies investigating the relationship of PA and quality of life in former elite cricketers^40 41^ highlighted a need for further research investigating the relationship between cricket participation and health. In collaboration with stakeholders, including governing bodies and current and former cricketers, a questionnaire was developed and refined. This information was disseminated through presentations to stakeholders.

### Participants and recruitment

**Figure 1:**
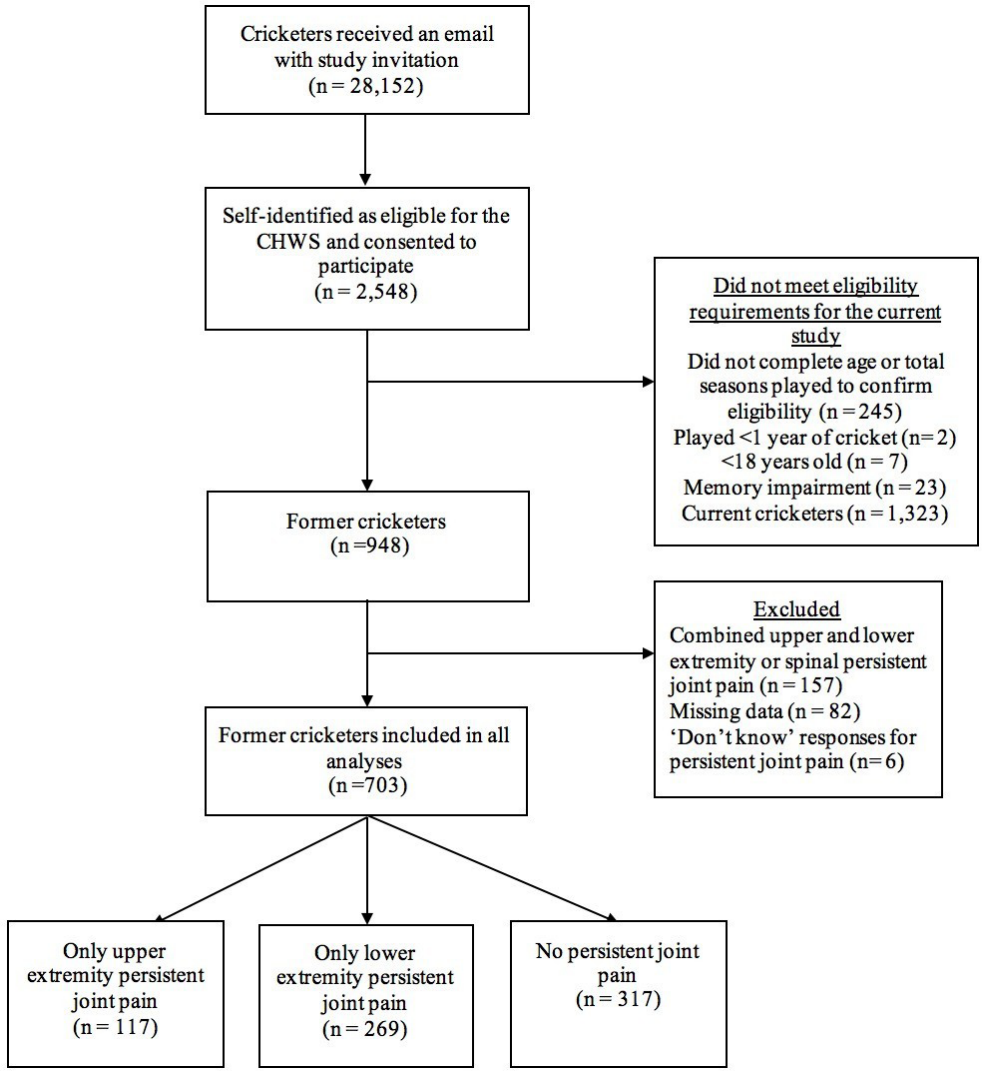
Study flow chart. CHWS, Cricket Health and Wellbeing Study.

In March 2017, 28 152 current and former cricketers of all playing standards, who were registered on a national database managed by the England and Wales Cricket Board, received an email inviting them to complete an electronic questionnaire. There were 2598 people who believed they met the eligibility requirements outlined in the email and consented to participate in the Cricket Health and Wellbeing Study (figure 1). Participants were eligible for inclusion in the Cricket Health and Wellbeing Study if they had played ≥1 cricket season and were aged ≥18 years. Due to the potential confounding relationship between acute injury and joint pain,^42^ only former cricketers were included in this study. The other eligibility requirement for inclusion in this study was reporting either (1) persistent joint pain only in the upper extremity, (2) persistent joint pain only in the lower extremity or (3) no persistent joint pain (individuals were excluded if they reported persistent back pain, or combined upper and lower extremity persistent joint pain). Participants were also excluded if they reported any memory impairment.

### Questionnaire design

The Cricket Health and Wellbeing Study questionnaire was developed in collaboration with public involvement, and four current/former cricketers piloted the questionnaire. The questionnaire was created to evaluate five aspects of health and well-being: (1) cricket-related injury; (2) joint pain and OA; (3) general health and disease prevalence; (4) PA; and (5) resilience, quality of life and flourishing. All participant data were managed and de-identified in a REDCap (Research Electronic Data Capture) software database. The REDCap database was managed by one investigator and pilot-tested by three researchers for errors and overall usability.^43 44^ The REDCap software^44^ used branching logic and allowed participants the option to save their current progress and complete at a later time. The questionnaire captured data including cricketers’ age, body mass index (BMI), number of cricket seasons played, playing standard, presence of comorbidities, persistent pain, medically diagnosed OA, PA and HRQoL.

### Outcomes

#### Physical activity

The International Physical Activity Questionnaire-Short Form (IPAQ-SF) was used to assess PA.^45^ The IPAQ-SF has been used as a population surveillance and evaluation PA tool.^46 47^ The IPAQ-SF has been observed to have fair agreement with accelerometer data (0.03–0.39)^48 49^ and good validity compared with other PA questionnaires and PA logs (r>0.50).^49 50^ Participants were asked to recall their PA in a usual week, as time spent in vigorous-intensity PA, moderate-intensity PA and walking.^45^ To reduce variability, a standardised approach outlined in the IPAQ-SF manual was used to clean and code the IPAQ-SF data.^45^ In line with recommendations, bouts of weekly PA less than 10 min were recoded as 0 minutes.^45^ Time spent in vigorous PA, moderate PA and walking was converted to metabolic equivalents (METS). One MET is 1 kcal/kg/hour, or the resting metabolic rate, during quiet sitting.^45^ Vigorous PA was calculated as 8 METS per minute, moderate PA was calculated as 4 METS per minute, and walking was calculated as 3.3 METS per minute.^45^ METS were truncated at 3 hours (180 min) per week, per vigorous, moderate and walking PA.^45^ This was performed in order to reduce participant overestimation effect.^45^ A total of 117 (17%) participants had PA reports truncated. METS were then combined for vigorous PA, moderate PA and walking, and reported as total weekly METS.

#### Health-related quality of life

The Short-Form 8 (SF-8) was used to assess HRQoL.^51^ The SF-8 is a short version of the RAND 36-Item Health Survey (SF-36) V.1.0,^52^ and is scaled and measured on the same point scale (0–100) as the SF-36, with 0 representing maximum disability and 100 representing no disability.^53^ The SF-8 is an eight-item, self-reported HRQoL questionnaire comprising eight domains (general health perceptions, physical function, bodily pain, physical role function, emotional role function, social function, vitality and mental health)^53^ and two component summary scores (physical component score (PCS) and mental component score (MCS)). PCS and MCS have been found to have high reliability (0.88 and 0.82, respectively) in the general US population.^54^ PCS and MCS are calculated using a computerised algorithm and weighted to the US population mean.^54^ A score of 50 is considered the population norm, and a score below 47 is considered below the average range of the general population.^54^ The minimum detectable difference for PCS in patients with lower extremity OA is 2 points.^55^ The minimally clinically important difference is estimated to be 3–5 points in the US general population.^56^

### Explanatory variables

#### Persistent joint pain

Persistent joint pain was assessed with the following question: *‘Do you currently experience pain, discomfort, or have any problems in any of your joints?’* If yes, participants were then asked *‘Have you had pain in your [left/right] hip/groin, knee, ankle, shoulder, hand/finger, spine/back, other joint on most days of the last month?’*. All participants who recorded persistent pain in the ‘other joint’ category were hand-searched for references of elbow, wrist, foot or toe persistent joint pain. These data were then recorded into separate categories of elbow/wrist and foot/toes persistent joint pain. All persistent joint pain data were then separated into upper extremity persistent joint pain only (shoulder, elbow, wrist, hand and/or fingers), lower extremity persistent joint pain only (hip, knee, ankle, foot and/or toes) and no persistent joint pain. If participants recorded spinal persistent pain or upper and lower extremity persistent joint pain, subjects were excluded from the analyses.

#### Standard of play

Standard of play was assessed with the following question: *‘* *what* *was the highest standard of cricket that you played for at least one* *season?* *’* Response options included international, county/premier league, academy or county age group, university, school, village or social, and don’t know. Participants were stratified into recreational (university, school, village or social) and elite (international or county/premier league, academy or county age group). Participants who reported a playing standard as *‘Don’t know’* were excluded from all analyses.

#### Confounders

Potential confounders were identified through clinical reasoning and a review of the literature. Confounders included age, BMI and presence of a comorbidity. Participants reported all medical problems that they had been diagnosed with. For the current study, diabetes, stroke and cancer were included as confounders due to a large potential to impact HRQoL and PA levels.^57–61^ Presence of a comorbidity was defined as no comorbidity versus one or more comorbidity.

### Statistical analyses

All data were assessed for missingness prior to analyses. Missing data were calculated as total and percentage of total data (online supplementary appendix 1). Due to the low percentage of missing data (MCS: 8.6%, PCS: 8.4%, IPAQ-SF: 5.9%, persistent joint pain: 1.1%), a complete case analysis was performed. ‘Don’t know’ responses were excluded from all analyses. The Kruskal-Wallis test was used to compare PA and HRQoL between pain groups. To assess specific group to group differences, a series of Dunn’s post-hoc analyses were performed (p<0.05).

10.1136/bmjopen-2019-032606.supp1 Supplementary data

Continuous data were not assumed to act linearly on the outcomes. Thus, to evaluate the relationship between persistent joint pain, PA levels and HRQoL, multivariable linear regressions with fractional polynomials were used (to account for the non-linearity of the covariates).^62^ This analysis was repeated in recreational and elite cricketer subgroups to address the second aim of this study. Unadjusted and adjusted coefficients and 95% CI were calculated. Coefficients were adjusted for the continuous variables age and BMI and the presence of ≥1 comorbidity (yes vs no). All assumptions for fractional polynomial regression were evaluated and satisfied.^62^ Transformations included taking the square root of METS; PCS and MCS were not transformed due to regression residuals. Possible interactions were assessed for inclusion in the model. Interactions explored were between age and BMI, age and comorbidity, BMI and comorbidity, age and persistent pain, BMI and persistent pain, and comorbidity and persistent pain. All analyses were performed in R V.3.5.1 (R Core Team (2013); R: A language and environment for statistical computing, R Foundation for Statistical Computing, Vienna, Austria, URL http://www.R-project.org/), using the dplyr package^63^ for cleaning and coding, the naniar package for missingness assessment,^64^ and the mfp package for fractional polynomial regression.^65^

## Results

A total of 703 former cricketers (aged mean 58.7, SD 12.9, played an average of 30 (IQR 20–40) seasons, 38% of whom had played at an elite level) were included in analyses (table 1). BMI in people with upper extremity persistent joint pain was 27.9 (SD 5.0), lower extremity persistent joint pain was 29.1 (SD 5.5) and in people with no persistent joint pain was 27.8 (SD 5.4).

**Table 1:** Participant characteristics

	All former cricketers (n=703)	Upper extremity persistent joint pain (n=117)	Lower extremity persistent joint pain (n=269)	No persistent joint pain (n=317)
Age (years)	58.7 (SD 12.9)	59.9 (SD 12.6)	59.8 (SD 11.8)	57.8 (SD 13.8)
Sex				
Male	683 (97%)	113 (97%)	263 (98%)	307 (97%)
Female	15 (3%)	3 (3%)	4 (2%)	8 (3%)
NR	5 (<1%)	1 (<1%)	2 (<1%)	2 (<1%)
BMI (kg/m^2^)	28.3 (SD 5.3)	27.9 (SD 5.0)	29.1 (SD 5.5)	27.8 (SD 5.4)
Seasons played	30 (IQR 20–40)	35 (IQR 27–44)	32 (IQR 23–42)	30 (IQR 18–48)
Years since last cricket match	7.4 (IQR 0.4–14.4)	8.1 (IQR 0.8–15.4)	6.9 (IQR 0.1–13.7)	6.7 (IQR 0.1–13.3)
Playing standard				
Elite	264 (38%)	46 (39%)	109 (41%)	109 (34%)
Recreational	428 (61%)	68 (58%)	156 (58%)	204 (65%)
NR	11 (1%)	3 (3%)	4 (1%)	4 (1%)
Comorbidity				
No	497 (73%)	81 (74%)	186 (71%)	230 (75%)
Yes	155 (23%)	23 (21%)	64 (25%)	68 (22%)
NR	27 (4%)	6 (5%)	10 (4%)	11 (4%)

Participants reported the highest standard they had played for at least one season and then were stratified into recreational (university, school, village or social) and elite (international or county/premier league, academy or county age group).

BMI, body mass index; NR, no response.

### A comparison of PA and HRQoL between former cricketers with upper extremity persistent joint pain, lower extremity persistent joint pain and no persistent joint pain

**Figure 2:**
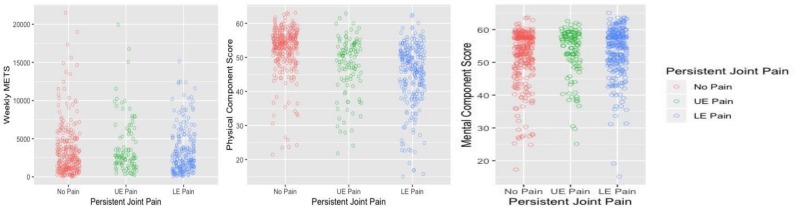
Weekly physical activity levels and health-related quality of life in former cricketers with upper extremity persistent joint pain, lower extremity persistent joint pain or no persistent joint pain. LE, lower extremity; METS, metabolic equivalents; UE, upper extremity.

For a visual representation of the PA data, please refer to figure 2. Kruskal-Wallis test indicated that there was no difference in PA levels between former cricketers with upper extremity persistent joint pain (median (IQR): 2560 (722–4398) METS), lower extremity persistent joint pain (2215 (527–3903) METS) or no persistent joint pain (2449 (695–4203) METS) (χ^2^: 1.91, p=0.39). There were significant PCS differences between pain groups (p<0.001). Post-hoc tests demonstrated that former cricketers reported worse PCS if they had persistent joint pain in the upper extremity (PCS: 49.8 (44.9–54.8)) or lower extremity (PCS: 46.7 (41.0–51.9)), compared with former cricketers with no persistent joint pain (PCS: 54.2 (51.5–56.9)) (χ^2^: 121.2, p<0.001). Former cricketers with lower extremity persistent joint pain reported worse PCS compared with former cricketers with upper extremity persistent joint pain (median difference: 1.8, p=0.04). MCS were similar between individuals with upper extremity persistent joint pain (56.0 (52.1–60.0)), lower extremity persistent joint pain (55.2 (51.1–59.4)) and no persistent joint pain (54.7 (50.7–58.7)) (χ^2^: 1.92, p=0.38).

### The relationship between persistent joint pain, PA and HRQoL

Multivariable linear regressions found that persistent joint pain was not related to PA levels (upper extremity: adjusted effect (95% CI): 28.1 METS (−1.1 to 135.7); lower extremity: 4.6 METS (−7.5 to 49.4); see table 2). Former cricketers with upper extremity persistent joint pain reported an estimated −5.5 (−7.4 to −3.5) points lower (worse) PCS than cricketers without persistent joint pain. Former cricketers with lower extremity persistent joint pain reported an estimated −6.6 (−8.1 to −5.2) points lower PCS compared with those with no persistent joint pain (table 2). Persistent joint pain was not associated with MCS (upper extremity: −0.1 (−0.2 to 0.1); lower extremity: −0.1 (−0.2 to 0.1)).

**Table 2:** Relationship between persistent joint pain, physical activity and health-related quality of life

	Weekly METS¶		PCS**		MCS**	
	Unadjusted effect* (95% CI)	Adjusted† effect* (95% CI)	Unadjusted effect‡ (95% CI)	Adjusted† effect‡ (95% CI)	Unadjusted effect‡ (95% CI)	Adjusted† effect‡ (95% CI)
Upper extremity persistent joint pain§	20.3 (−3.2 to 117), p=0.83	28.1 (−1.1 to 136), p=0.10	−5.4 (−7.3 to 3.6), p<0.001	−5.5 (−7.4 to 3.5), p<0.001	−0.1 (−0.2 to 0.1), p=0.35	−0.1 (−0.2 to 0.1), p=0.26
Lower extremity persistent joint pain§	0.3 (−12.5 to 21.0), p=0.16	4.6 (−7.5 to 49.4), p=0.39	−6.9 (−8.4 to 5.5), p<0.001	−6.6 (−8.1 to 5.2), p<0.001	0.09 (−0.2 to 0.1), p=0.08	−0.1 (−0.2 to 0.1), p=0.06
No persistent joint pain	Reference group		Reference group		Reference group	

*Participants with memory impairments were excluded from the analyses.

†Estimates are adjusted for age, body mass index and comorbidities.

‡Comorbidities were defined as none present (0) and presence of at least one comorbidity (1). Comorbidities included were diabetes, stroke, skin cancer and other cancer.

§Upper extremity (shoulder, elbow, wrist or hand) and lower extremity (hip, knee or ankle) persistent joint pain were assessed by asking individuals if they had joint-specific pain on ‘most days of the last month’.

¶Short-form questionnaire (International Physical Activity Questionnaire-Short Form). Physical activity was calculated as METS per week; METS were transformed prior to analysis by taking the square root and then retransformed by squaring after analysis.

**Short-Form 8 Health Survey. PCS and MCS were calculated using norm-based scoring (population norm 50, SD 10, high scorer=better health-related quality of life).

MCS, mental component score; METS, metabolic equivalents; PCS, physical component score.

**Table 3:** Relationship between persistent joint pain, physical activity and health-related quality of life in former recreational and elite cricketer subgroups

	Weekly METS		PCS		MCS	
	Unadjusted effect* (95% CI)	Adjusted† effect* (95% CI)	Unadjusted effect‡ (95% CI)	Adjusted† effect‡ (95% CI)	Unadjusted effect‡ (95% CI)	Adjusted† effect‡ (95% CI)
Former elite cricketers (n=280)						
Upper extremity persistent joint pain	70 (−1.6 to 324), p=0.09	88 (−0.1 to 356), p=0.05	−5.3 (−8.6 to 2.0), p=0.001	−5.3 (−8.8 to 1.9), p=0.002	0.055 (−2.3 to 3.4), p=0.70	5.3 (−1.1 to 11.7), p=0.10
Lower extremity persistent joint pain	5.2 (−28 to 97), p=0.56	8.2 (−21 to 106), p=0.45	−7.5 (−10.1 to 4.9), p<0.001	−7.0 (−9.7 to 4.3), p<0.001	0.1 (−1.7 to 2.7), p=0.64	2.2 (−2.7 to 7.0), p=0.39
No persistent joint pain	Reference group		Reference group		Reference group	
Former recreational cricketers (n=423)						
Upper extremity persistent joint pain	5.6 (−37 to 117), p=0.58	10.5 (−29 to 149), p=0.47	−5.6 (−7.9 to 3.3), p<0.001	−5.8 (−8.1 to 3.4), p<0.001	0.8 (−1.5 to 3.0), p=0.50	1.1 (−1.1 to 3.2), p=0.35
Lower extremity persistent joint pain	−0.7 (−49 to 29), p=0.83	1.8 (−26 to 61), p=0.68	−6.6 (−8.3 to 4.9), p<0.001	−6.2 (−8.0 to 4.5), p<0.001	−0.8 (−1.1 to 2.7), p=0.33	1.2 (−0.5 to 2.8), p=0.17
No persistent joint pain	Reference group		Reference group		Reference group	

*Participants with memory impairments were excluded from the analyses.

†Estimates are adjusted for age, body mass index and comorbidities.

‡Comorbidities were defined as not present (0) and presence of at least one comorbidity (1). Comorbidities included were diabetes, stroke, skin cancer and other cancer.

§Upper extremity (shoulder, elbow, wrist or hand) and lower extremity (hip, knee or ankle) persistent joint pain were assessed by asking individuals if they had joint-specific pain on ‘most days of the last month’.

¶Short-form questionnaire (International Physical Activity Questionnaire-Short Form). Physical activity was calculated as METS per week; METS were transformed prior to analysis by taking the square root and then retransformed by squaring after analysis.

**Short-Form 8 Health Survey. PCS were calculated using norm-based scoring (population norm 50, SD 10, high scorer=better health-related quality of life). MCS were calculated using norm-based scoring (population norm 50, SD 10, high scorer=better health-related quality of life).

MCS, mental component score; METS, metabolic equivalents; PCS, physical component score.

### Elite versus recreational cricketer subgroup analysis

Multivariable linear regressions found that findings were similar between recreational and elite cricketer subgroups (table 3). Persistent joint pain was not associated with PA and MCS, and PCS were impaired in both former elite and recreational cricketers with upper extremity (adjusted effect (95% CI) elite: −5.3 (−8.8 to –1.9); recreational: −5.8 (−8.1 to –3.4)) or lower extremity (elite: −7.0 (−9.7 to –4.3); recreational: −6.2 (−8.0 to –4.5)) persistent joint pain, compared with their counterparts with no persistent joint pain (table 3).

## Discussion

Surprisingly, despite impaired PCS, there was no difference in weekly PA or MCS between former cricketers with upper or lower extremity persistent joint pain and those with no persistent joint pain. Additionally, former cricketers with lower extremity persistent joint pain reported worse physical components of HRQoL than those with upper extremity persistent joint pain. We also found that the relationship between joint pain, PA and HRQoL was similar among former elite and recreational cricketer subgroups.

Former cricketers with persistent joint pain did not report reduced weekly PA compared with former cricketers without persistent joint pain. In the general population, people with OA have reduced PA levels compared with matched controls without OA.^66^ In this study, former cricketers with and without persistent joint pain had higher PA levels compared with the general population.^67–69^ Former cricketers reported median daily PA levels of 120 METS per day; this is in comparison with an average range of 41–50 METS per day for European men.^67–69^ Former cricketers may find alternative strategies to exercise, despite persistent joint pain.^41 70^ In a qualitative study, former cricketers with pain and physical impairment found alternative strategies to perform PA and shared specific psychological strengths that may enable them to effectively cope with joint pain.^41^ Intrinsic motivation,^71^ higher levels of resilience^32 40^ and effective coping strategies^40 41^ are all characteristics common among successful athletes. Such psychological strengths may be more common in former sport participants compared with the general population.^29–32^ Additionally, sport participants may be encouraged to play through pain^72^ and are often rewarded for doing so.^73^ Such pain behaviours could influence how former sport participants perceive and cope with joint pain. Pain behaviours and psychological strengths common among sport participants could provide a potential explanation for the high PA levels in former cricketers living with joint pain. Further research is required to understand the potential interplay between persistent pain, psychological characteristics and PA in former athletes.

The psychological strengths that are common among former sports participants, including resilience,^32 40^ mental toughness^74 75^ and pain coping strategies,^40 75^ could also provide a potential explanation for why MCS were not impaired in former cricketers with persistent joint pain, despite impaired PCS. In a recent meta-analysis, former athletes with impaired PCS reported greater MCS compared with general population norms.^76^ In a typical OA population, both PCS and MCS are impaired,^77^ suggesting HRQoL in former sport participants with OA may differ from the typical OA population. Furthermore, the high PA levels in former cricketers in our study could also contribute to the high MCS. PA has been shown to have a protective effect against depression and a positive impact on the mental components of HRQoL in the general population, older adults and in former elite athletes.^78–81^ Irrespective of joint pain, former cricketers reported MCS of 5–6 points better than the population normative average, and this difference is likely to be clinically meaningful.^82^

Former cricketers with persistent joint pain had impaired physical components of HRQoL compared with cricketers without persistent pain. This supports previous literature which found impaired PCS in former collegiate^83–85^ and professional athletes^86^ compared with non-athlete controls. Former cricketers with persistent pain reported PCS of 5–7 points lower than former cricketers without persistent pain, demonstrating a clinically important difference between persistent pain and no pain groups. Impaired physical components of HRQoL in former athletes have been attributed to poor joint health,^83–85^ which may be due to previous musculoskeletal injury.^87–89^ Former athletes with a history of musculoskeletal injury report worse physical components of HRQoL than former athletes without a history of injury^87^ and the general population.^88 89^ In our study, former cricketers without persistent pain reported a median PCS of 4 points above the population normative mean of 50, suggesting that former cricket participants without joint pain have greater levels of physical functioning compared with the general population. Importantly, despite impaired PCS in former cricketers with persistent pain, MCS were not impaired. This highlights the importance of selecting an HRQoL measure that differentiates assessment of pain and physical function from assessment of mental health and well-being when evaluating HRQoL in former sport participants.

Cricketers with persistent lower extremity pain reported 3.1 points worse PCS compared with cricketers with upper extremity pain, and this difference is likely to be clinically meaningful.^82^ Worse PCS in former cricketers with lower extremity pain may be due to greater difficulties performing usual PAs (such as difficulties with ambulation and stair navigation^90 91^) or experiencing more severe bodily pain than those who report upper extremity pain. For example, former collegiate athletes with lower extremity persistent pain were 2.5 times more likely to be limited in physical function, such as stair navigation, and have greater impaired HRQoL, compared with non-athletes.^84^ While there is research investigating the impacts of lower extremity pain on function in former athletes, our understanding of the impact of upper extremity pain on function in former athletes is poor. Further research could inform strategies to improve physical components of HRQoL among former athletes with persistent upper extremity pain.

The relationship between persistent joint pain, PA and HRQoL was similar between elite and recreational former cricketers. Few studies have investigated PA or HRQoL in former athletes from different standards of play. Higher standards of sport competition require elevated levels of resilience and psychological hardiness due to the increased levels of training and competition.^29–32 92^ Despite the observed psychological strengths associated with elite cricket participation, any level of sport participation is associated with favourable psychological strengths such as resilience.^30 32 92^ Thus, it is possible that all standards of cricket participation are associated with psychological benefits that may explain the high PA levels and mental components of HRQoL in former cricketers living with persistent pain; further research is needed to explore this possibility.

### Strengths and potential limitations

This study took into account the non-linear relationships between different continuous variables (ie, by using fractional polynomials), where most studies assume linearity.^93^ Missing data were low, decreasing the risk of bias in a complete case analysis. PA was truncated above 180 min, which may introduce a ceiling effect. Further, PA recall may not be as precise as PA monitors, which may introduce measurement error, and social desirability may cause PA overestimation.^94 95^ Persistent joint pain was assessed by asking cricketers if they had joint pain on most days of the last month, which is based on the National Health and Nutrition Examination Survey. However, joint pain may occur intermittently and still affect PA and HRQoL. As a result, this study may have missed participants with significant pain. Participants with persistent pain in both the upper and lower extremities and participants with back pain were excluded from the analyses. This limits the scope of this study to participants with only upper or lower extremity persistent pain, or no persistent pain, decreasing the generalisability of these findings. The literature on SF-8 minimal detectable difference and minimal clinically important difference is sparse and does not evaluate athletic populations. This decreases the interpretability of these data. There is also the possibility of bias due to unmeasured confounding. Potential confounders that were not measured in this study include socioeconomic status and other sports played, which both have been observed to be related to HRQoL.^76 96 97^ These discrepancies may decrease the generalisability of these findings. Potential participants were informed of the inclusion and exclusion criteria during recruitment and were able to self-select eligibility to participate. Due to this recruitment strategy, it is not possible to determine the questionnaire response rate nor responder bias. This recruitment strategy may decrease the generalisability of these findings.

## Conclusions

Physical components of HRQoL were impaired in former cricketers with persistent joint pain compared with those without joint pain, and PCS were more impaired in those with lower extremity pain compared with those with upper extremity pain. However, former cricketers with persistent joint pain did not have reduced PA levels or mental components of HRQoL, compared with those with no persistent joint pain. These relationships were similar among former elite and recreational cricketers. These results highlight the need to select an HRQoL measure that differentiates assessment of pain and physical functional from assessment of mental health and well-being when evaluating HRQoL in former sport participants. The high average PA levels among former cricketers with persistent joint pain could explain the high MCS in this group. In contrast, former cricketers with joint pain who are physically inactive may benefit from targeted strategies to increase activity levels, with potential to positively impact quality of life. Further research is needed to determine whether the psychological benefits of sport participation explain why former cricketers had high PA levels and mental components of HRQoL, despite living with persistent joint pain.

## Supplementary Material

Reviewer comments

Author's manuscript
