# Supplementary material for: Physical activity and health-related quality of life in former elite and recreational cricketers from the UK with upper extremity or lower extremity persistent joint pain: a cross-sectional study
Source: BMJ Open. 2019 Nov 11;9(11):e032606. doi: 10.1136/bmjopen-2019-032606 (PMC6858171; doi:10.1136/bmjopen-2019-032606)
Supplement: Supplementary data [file bmjopen-2019-032606supp001.pdf]

**Appendix 1.** Missing Data Responses in Former Cricketers

| Variable | Count | Percentage |
|---|---|---|
| Physical Component Score | 82 | 8.6% |
| Mental Component Score | 80 | 8.4% |
| Body mass Index | 61 | 6.3% |
| IPAQ-SF | 56 | 5.9% |
| Cricket Seasons | 12 | 1.7% |
| Persistent Pain | 10 | 1.4% |
| Osteoarthritis | 10 | 1.4% |
| Other Cancer | 10 | 1.4% |
| Skin Cancer | 8 | 1.1% |
| Stroke | 8 | 1.1% |
| Diabetes | 1 | 0.1% |
| Age | 0 | 0% |
