# Supplementary material for: Physical activity and health-related quality of life in former elite and recreational cricketers from the UK with upper extremity or lower extremity persistent joint pain: a cross-sectional study
Source: BMJ Open. 2019 Nov 11;9(11):e032606. doi: 10.1136/bmjopen-2019-032606 (PMC6858171; doi:10.1136/bmjopen-2019-032606)
Supplement: Author's manuscript [file bmjopen-2019-032606.draft_revisions.pdf]

BMJ Open

BMJ Open is committed to open peer review. As part of this commitment we make the peer review history of every article we publish publicly available.

When an article is published we post the peer reviewers' comments and the authors' responses online. We also post the versions of the paper that were used during peer review. These are the versions that the peer review comments apply to.

The versions of the paper that follow are the versions that were submitted during the peer review process. They are not the versions of record or the final published versions. They should not be cited or distributed as the published version of this manuscript.

BMJ Open is an open access journal and the full, final, typeset and author-corrected version of record of the manuscript is available on our site with no access controls, subscription charges or pay-per-view fees (http://bmjopen.bmj.com).

If you have any questions on BMJ Open's open peer review process please email info.bmjopen@bmj.com

BMJ Open

Physical activity and health related quality of life in former elite and recreational cricketers with upper extremity or lower extremity persistent joint pain

Journal:	*BMJ Open*
Manuscript ID	bmjopen-2019-032606
Article Type:	Research
Date Submitted by the Author:	26-Jun-2019
Complete List of Authors:	Bullock, Garrett; University of Oxford, Nuffield Department of Orthopaedics, Rheumatology, and Musculoskeletal Sciences<br>Collins, Gary; University of Oxford, Centre for Statistics in Medicine<br>Peirce, Nicholas; Nottingham University Hospitals Trust, Centre For Sports Medicine; England and Wales Cricket Board, National Cricket Performance Centre<br>Arden, Nigel; Nuffield Department of Orthopaedics, ;<br>Filbay, Stephanie; University of Oxford, Arthritis Research UK Centre for Sport, Exercise & Osteoarthritis, Nuffield Department of Orthopaedics, Rheumatology & Musculoskeletal Sciences
Keywords:	Shoulder < ORTHOPAEDIC & TRAUMA SURGERY, Knee < ORTHOPAEDIC & TRAUMA SURGERY, ankle, Hip < ORTHOPAEDIC & TRAUMA SURGERY, hand, retired athlete

SCHOLARONE™
Manuscripts

Physical activity and health related quality of life in former elite and recreational cricketers with upper extremity or lower extremity persistent joint pain

Garrett S. Bullock PT, DPT,[1, 2] Gary S. Collins Bsc (Hons), PhD,[3,4] Peirce N., BMBS, FRIS,[5, 6] Nigel K. Arden, MD, FRCP,[1, 2] Stephanie R. Filbay, B.Phty (Hons), PhD[1, 2]

1. Centre for Sport, Exercise and Osteoarthritis Research *Versus Arthritis,* University of Oxford, United Kingdom
2. Nuffield Department of Orthopaedics, Rheumatology, and Musculoskeletal Sciences, University of Oxford, Oxford, United Kingdom
3. Centre for Statistics in Medicine, Nuffield Department of Orthopaedics, Rheumatology, and Musculoskeletal Sciences, University of Oxford, Oxford United Kingdom
4. Oxford University Hospitals NHS Foundation Trust, Oxford, United Kingdom
5. England and Wales Cricket Board, National Cricket Performance Centre, Loughborough University, LE11 3TU, United Kingdom
6. National Centre for Sport and Exercise Medicine, Loughborough University, LE11 3TU, United Kingdom

Corresponding Author Information

Garrett S. Bullock PT, DPT
Nuffield Department of Orthopaedics, Rheumatology, and Musculoskeletal Sciences
University of Oxford
B4495
Oxford, United Kingdom
OX3 7LD
(865) 227-374
garrett.bullock@ndorms.ox.ac.uk

Key Words: Shoulder, Knee, Ankle, Hip, Hand, Sport, Retired Athletes

Word Count: 3518

Conflict of Interest: Dr. Nick Peirce is affiliated with the England and Wales Cricket Board. All other authors affirm that they have no involvement with any commercial organization that has a direct financial interest in any matter included in this manuscript.

For peer review only - http://bmjopen.bmj.com/site/about/guidelines.xhtml

Abstract

Objectives: Evaluate and compare physical activity (PA) and health-related quality-of-life (HRQoL) in former elite and recreational cricketers with upper extremity (UE), lower extremity (LE), or no joint pain.

Study Design: Cross-sectional cohort

Setting: Despite the high prevalence of joint pain in former athletes, the impact of UE-pain and LE-pain on PA and HRQoL, and potential differences between former recreational and elite athletes, is poorly understood.

Participants: 703 former cricketers aged ≥18 years (aged mean 58.7 SD 12.9, played an average 30 IQR 20-40 seasons, 72% had played at a recreational level) were recruited through the Cricket Health and Wellbeing Study and met eligibility requirements (UE-pain, LE-pain or no joint pain (defined as pain on most days of the past month)).

Primary and Secondary Outcomes: A questionnaire collected IPAQ-SF (weekly metabolic equivalents (METS)) and SF-8 (physical (PCS) and mental (MCS) component scores). Kruskall-Wallis tests with Dunn's post-hoc and multivariable linear regressions were performed.

Results: Weekly METS were similar in former cricketers with UE-pain (median(IQR) 2560(722-4398)), LE-pain (2215(527-3903)) and no pain (2449(695-4203), p=0.39). MCS scores were similar between groups (UE-pain 56.0(52.1-60.0); LE-pain 55.2(51.1-59.4); no pain 54.7(50.7-58.7), p=0.38). PCS scores were more impaired in former cricketers with UE-pain (49.8(44.9-54.8)) or LE-pain (46.7(41.0-51.9)) compared to no pain (54.2(51.5-56.9), p<0.0001). Former cricketers with LE-pain reported worse PCS scores than those with UE-pain (p=0.04). Similar relationships were observed in former elite and recreational cricketers.

Conclusion: Despite impaired physical-components of HRQoL in former cricketers with UE-pain or LE-pain, pain was not related to PA levels or mental-components of HRQoL. Physical-components of HRQoL was most impaired in those with LE-pain, and findings were similar amongst former elite and recreational cricketers.

Key Words: Shoulder, Knee, Ankle, Hip, Hand, Sport, Retired Athletes

Strengths and Limitations:

- This study took into account the non-linear relationships between different continuous variables (i.e. by using fractional polynomials), where most studies assume linearity.
- Missing data was low, allowing for low bias in a complete case analysis.
- Participants with persistent pain in both the upper and lower extremities, and participants with back pain were excluded from the analyses, decreasing the generalizability of these findings.
- The SF-8 minimal detectable difference and minimal clinical important difference literature is sparse, and does not evaluate athletic populations. This decreases the interpretability of these data.

For peer review only - http://bmjopen.bmj.com/site/about/guidelines.xhtml

INTRODUCTION

Upper and lower extremity osteoarthritis (OA) has a high incidence[1 2] and a substantial personal and societal burden.[3 4] Post-traumatic OA occurs at a younger age, with a longer period of disability and less effective treatments than idiopathic OA.[5-7] Former sport participants have increased post-traumatic OA prevalence compared to the general population,[5 8] which is associated with persistent joint pain.[9] Persistent joint pain is the major cause of patients seeking medical attention for OA,[9] and has been shown to influence a person's ability to participate in desired forms of activity[10 11] which may result in reduced physical activity levels and negatively impact health-related quality of life (HRQoL).[10-13]

To date, most research investigating OA and pain has focused on the lower extremity.[10-14] Former professional soccer players were found to have a high prevalence of lower extremity OA and pain compared to controls,[10 11] while former female recreational athletes were found to have 2 to 3 times increased hip and knee OA risk compared to population matched controls.[14] Lower extremity OA and pain in former sport participants is associated with impaired function and mobility, increased levels of anxiety and depression, and reduced HRQoL compared to the general population.[10 12-14]

Upper extremity pain can have different barriers to PA participation compared to lower extremity pain.[15-18] For example, lower extremity pain may have a greater effect on jumping, stair use, walking and running.[19 20] In contrast, upper extremity pain can have more effect on specific functional activities such as dressing and eating[15] or work related tasks such as carrying and handling objects[16] with potential negative impacts on HRQoL.[15] Upper extremity pain has been related to reduced PA and function, impaired HRQoL, stress and disability in workers.[21-25] Despite a high prevelance of upper extremity pain in former

For peer review only - http://bmjopen.bmj.com/site/about/guidelines.xhtml

throwing-sport participants[26-28]the impact of upper extremity pain on PA levels and HRQoL in former athletes is poorly understood.

Psychological characteristics associated with successful high level athletic performance include resilience, mental toughtness and advanced coping skills.[29-32] Greater resilience and coping strategies can have positive impact on PA levels[33-35] and HRQoL.[34 36-38] Thus, former elite athletes may possess psychological characteristics that enable them to better cope with chronic pain compared to former recreational sport participants, and this could positiviely impact PA levels and HRQoL. However, this has not yet been investigated, highlighting the need for further research. It is also not clear if PA levels and HRQoL differ in former sport participants with and without perisistent joint pain. The purpose of this was to:

1) evaluate the relationship between persistent joint pain (upper extremity, lower extremity, or no persistent joint pain), PA levels and HRQoL in former cricketers;
2) compare these relationships in former elite and recreational cricketer subgroups.

METHODS

Study Design

This cross-sectional study was approved by the [redacted]

Participants and recruitment

In March 2017, 28,152 current and former cricketers of all playing standards, who were registered on a national database managed by the England and Wales Cricket Board, received an email inviting them to complete an electronic questionnaire. 2,598 people believed they met the eligibility requirements outlined in the email, and consented to participate in the

For peer review only

Cricket Health and Wellbeing Study (Figure 1). Participants were eligible for inclusion in the Cricket Health and Wellbeing Study if they had played ≥1 cricket season and were aged ≥18 years. Due to the potential confounding relationship between acute injury and joint pain,[39] only former cricketers were included in this study. The other eligibility requirement for inclusion in this study, was reporting either i) persistent joint pain in only the upper extremity, ii) persistent joint pain in only the lower extremity, or iii) no persistent joint pain (individuals were excluded if they reported persistent back pain, or combined upper and lower extremity persistent joint pain). Participants were also excluded if they reported any memory impairment.

Questionnaire design

The Cricket Health and Wellbeing Study questionnaire was developed in collaboration with public involvement and four current/former cricketers piloted the questionnaire. The questionnaire was created to evaluate five aspects of health and wellbeing (i. cricket-related injury; ii. joint pain and osteoarthritis; iii. general health and disease prevalence; iv. physical activity; v. resilience, quality of life and flourishing). All participant data was managed and de-identified in a RedCap® (Research Electronic Data Capture) software database. The RedCap® database was managed by one investigator, and pilot tested by three researchers for errors and overall userbility.[40 41] The RedCap® software[41] utilized branching logic and allowed participants the option to save their current progress and complete at a later time. The questionnaire captured data including cricketers' age, body mass index (BMI), number of cricket seasons played, playing standard, presence of comorbidities, persistent pain, medically diagnosed OA, physical activity and HRQoL.

<Figure 1>

For peer review only

For peer review only - http://bmjopen.bmj.com/site/about/guidelines.xhtml

Outcomes

Physical activity

The International Physical Activity Questionnaire-Short Form (IPAQ-SF) was utilized to assess physical activity.[42] The IPAQ-SF has been used as a population surveillance and evaluation PA tool.[43 44] The IPAQ-SF has been observed to have fair agreement with accelerometer data (0.03-0.39)[45 46] and good validity compared to other PA questionnaires and PA logs (r>0.50).[46 47] Participants were asked to recall their PA in a usual week, as time spent in vigorous-intensity PA, moderate-intensity PA, and walking.[42] To reduce variability, a standardized approach outlined in the IPAQ-SF manual was utilized to clean and code the IPAQ-SF data.[42] In line with recommendations, bouts of weekly PA less than 10 minutes were recoded as zero minutes.[42] Time spent in vigorous PA, moderate PA, and walking was converted to Metabolic Equivalents (METS). One MET is 1 kcal/kg/h, or the resting metabolic rate, during quiet sitting.[42] Vigorous PA was calculated as 8 METS per min, moderate PA was calculated as 4 METS per min, and walking was calculated as 3.3 METS per min.[42] Metabolic equivalents were truncated at three hours (180 minutes) per week, per vigorous, moderate, and walking PA.[42] This was performed in order to reduce participant overestimation effect.[42] METS were then combined for vigorous PA, moderate PA, and walking, and reported as total weekly METS.

Health related quality of life

The Short-Form 8 (SF-8) was used to assess HRQoL.[48] The SF-8 is a short version of the RAND 36-Item Health Survey (SF-36) 1.0,[49] the SF-8 is scaled and measured on the same point scale (0-100) as the SF-36, with 0 representing maximum disability and 100 representing no disability.[50] The SF-8 is an 8-item, self-reported HRQoL questionnaire comprising 8 domains (general health perceptions, physical function, bodily pain, physical

For peer review only - http://bmjopen.bmj.com/site/about/guidelines.xhtml

role function, emotional role function, social function, vitality, and mental health)[50] and two component summary scores (the Physical Component Score (PCS) and Mental Component Score (MCS)). The PCS and MCS scores have been found to have high reliability (0.88 and 0.82 respectively) in the general United States population.[51] The PCS and MCS scores are calculated using a computerized algorithm, and weighted to the United States population mean.[51] A score of 50 is considered the population norm, and a score below 47 is considered below the average range of the general population.[51] The minimum detectable difference for the PCS in lower extremity OA patients is two points.[52] The minimally clinically important difference is estimated to be three to five points in the United States general population.[53]

Explanatory Variables

Persistent joint pain

Persistent joint pain was assessed with the following question, *'Do you currently experience pain, discomfort, or have any problems in any of your joints?'* If yes, participants were then asked *'Have you had pain in your [left/right] hip/groin, knee, ankle, shoulder, hand/finger, spine/back, other joint on most days of the last month?'*. All participants that recorded persistent pain in the "other joint" category were hand searched for references of elbow, wrist, foot, or toe persistent joint pain. These data were then recorded into separate categories of elbow/wrist, and foot/toes persistent joint pain. All persistent joint pain data was then separated into only upper extremity persistent joint pain (shoulder, elbow, wrist, hand and/or fingers), only lower extremity persistent joint pain (hip, knee, ankle, foot, and/or toes), and no persistent joint pain. If participants recorded spinal persistent pain, or upper and lower extremity persistent joint pain, subjects were excluded from the analyses.

Standard of play

For peer review only

Standard-of-play was assessed with the following question, '*what was the highest standard of cricket that you played for at least one season*?' Response options included: international; county/premier league; academy or county age group; university; school; village or social; don't know. Participants were stratified into recreational (university, school, village or social) and elite (international or county/premier league, academy or county age group). Participants that reported a playing standard as *'Don't know'* were excluded from all analyses.

Confounders

Potential confounders were identified through clinical reasoning and a review of the literature. Confounders included age, body mass index (BMI), and presence of a comorbidity. Particpants reported all medical problems that they had been diagnosed with. For the current study, diabetes, stroke and cancer were included as confounders due to a large potential to impact HRQoL and PA levels.[54-58] Presence of a comorbidity was defined as no comorbidity vs. one or more comorbidity.

Statistical Analyses

All data were assessed for missingness prior to analyses. Missing data were calculated as total and percentage of total data (Appendix 1). Due to the low percentage of missing data (MCS: 8.6%, PCS: 8.4%, IPAQ-SF: 5.9%, persistent joint pain: 1.1%), a complete case analysis was performed. 'Don't know' responses were excluded from all analyses. The Kruskall Wallis test was used to compare PA and HRQoL, between pain groups. To assess specific group to group differences, a series of Dunn's post hoc analyses were performed ($p<0.05$).

For peer review only

Continuous data were not assumed to act linearly on the outcomes. Thus, to evaluate the relationship between persistent joint pain, PA levels and HRQoL, multivariable linear regressions with fractional polynomials were used (to account for the non-linearity of the covariates).[59] This analysis was repeated in recreational and elite cricketer subgroups to address the second aim of this study. Unadjusted and adjusted coefficients and 95% confidence intervals (95% CI) were calculated. Coefficients were adjusted for the continuous variables age and BMI, and the presence of ≥1 comorbidity (yes vs. no). All assumptions for fractional polynomial regression were evaluated and satisfied.[59] Transformations included taking the square root of METS, PCS and MCS scores were not transformed due to regression residuals. Possible interactions were assessed for inclusion in the model. Interactions explored were between age and BMI, age and comorbidity, BMI and comorbidity, age and persistent pain, BMI and persistent pain, and comorbidity and persistent pain. All analyses were performed in R version 3.5.1 (R Core Team (2013). R: A language and environment for statistical computing. R Foundation for Statistical Computing, Vienna, Austria. URL http://www.R-project.org/), using the dplyr package[60] for cleaning and coding, the naniar package for missingness assessment,[61] and the mfp package for fractional polynomial regression.[62]

RESULTS

A total of 703 former cricketers (aged mean 58.7 SD 12.9, played an average 30 IQR 20-40 seasons, 38% had played at an elite level) were included in analyses (Table 1). Body mass index in people with upper extremity persistent joint pain was 27.9 (SD 5.0), lower extremity persistent joint pain 29.1 (SD 5.5), and in people with no persistent joint pain was 27.8 (SD 5.4).

<Table 1>

For peer review only

A comparison of physical activity and HRQoL between former cricketers with upper extremity persistent joint pain, lower extremity persistent joint pain, and no persistent joint pain

There was no difference in physical activity levels between former cricketers with upper extremity persistent joint pain (Median (IQR): 2560 (722-4398) METS), lower extremity persistent joint pain (2215 (527-3903) METS), or no persistent joint pain (2449 (695-4203) METS) (Chi-square: 1.91, p=0.39). Former cricketers reported worse PCS scores if they had persistent joint pain in the upper extremity (PCS: 49.8 (44.9-54.8)) or lower extremity (PCS: 46.7 (41.0-51.9)), compared to former cricketers with no persistant joint pain (PCS: 54.2 (51.5-56.9); Chi-square:121.2, p<0.0001). Former cricketers with lower extremity persistent joint pain reported worse PCS scores compared to former cricketers with upper extremity persistent joint pain (Median Difference: 1.8, p=0.04). MCS scores were similar between individuals with upper extremity persistent joint pain (56.0 (52.1-60.0)), lower extremity persistent joint pain (55.2 (51.1-59.4)), and no persistent joint pain (54.7 (50.7-58.7)); Chi-squared: 1.92, p=0.38) (Figure 2).

<Figure 2>

The relationship between persistent joint pain, physical activity and health related quality of life

Persistent joint pain was not related to physical activity levels (Upper Extremity: Adjusted effect (95% CI): 28.1 METS (-1.1 to 135.7); Lower Extremity: 20.3 METS (-3.2 to 116.6)), see Table 2. Former cricketers with upper extremity persistent joint pain reported an estimated -5.5 (-7.4 to -3.5) points lower (worse) PCS scores than cricketers without persistent joint pain. Former cricketers with lower extremity persistent joint pain reported an estimated -6.6 (-8.1 to -5.2) points lower PCS scores compared to those with no persistent

For peer review only - http://bmjopen.bmj.com/site/about/guidelines.xhtml

joint pain (Table 2). Persistent joint pain was not associated with MCS scores (Upper Extremity: -0.1 (-0.2 to 0.1); Lower Extremity: -0.1 (-0.2 to 0.1)).

<Table 2>

Elite vs. recreational cricketer subgroup analysis

Findings were similar between recreational and elite cricketer subgroups (Table 3). Persistent joint pain was not associated with physical activity and MCS scores, and PCS scores were impaired in both former elite and recreational cricketers with upper extremity (Adjusted effect (95% CI) Elite: -5.3 (-8.8, -1.9); Recreational: -5.8 (-8.1, -3.4)) or lower extremity (Elite: -7.0 (-9.7, -4.3); Recreational: -6.2 (-8.0, -4.5)) persistent joint pain, compared to their counterparts with no persistent joint pain (Table 3).

<Table 3>

For peer review only

DISCUSSION

Suprisingly, despite impaired PCS scores, there was no difference in weekly PA or MCS scores, between former cricketers with upper or lower extremity persistent joint pain, and those with no persistent joint pain. Additionally, former cricketers with lower extremity persistent joint pain reported worse physical-components of HRQoL than those with upper extremity persistent joint pain. We also found that the relationship between joint pain, PA and HRQoL were similar amougnst former elite and recreational cricketer subgroups.

Former cricketers with persistent joint pain did not report reduced weekly PA compared to former cricketers without persistent joint pain. In the general population, people with OA have reduced PA levels compared to matched controls without OA.[63] In this study, former cricketers with and without persistent joint pain had higher PA levels compared to the general population.[64-66] Former cricketers reported median daily PA levels of 120 METS per day, this is in comparison to an average range of 41 to 50 METS per day for European men.[64-66] Former cricketers may find alternative strategies to exercise, despite persistent joint pain.[67,68] In a qualitative study, former cricketers with pain and physical impairment found alternative strategies to perform PA, and shared specific psychological strengths that may enable them to effectively cope with joint pain.[68] Intrinsic motivation,[69] higher levels of resilience,[32,70] and effective coping strategies[68,70] are all characteristics common amongst successful athletes. Such psychological strengths may be more common in former sport participants compared to the general population.[29-32] Additionally, sport participants may be encouraged to play through pain[71] and are often rewarded for doing so.[72] Such pain behaviours could influence how former sport participants perceive and cope with joint pain. Pain behaviours and psychological strengths common amougnst sport participants could provide a potential explanation for the high physical activity levels in former cricketers living with joint pain.

For peer review only - http://bmjopen.bmj.com/site/about/guidelines.xhtml

Further research is required to understand the potential interplay between persistent pain, psychological characteristics, and PA in former athletes.

The psychological strengths that are common amoungst former sports participants including resilience,[32 70] mental toughness,[73 74] and pain coping strategies,[70 74] could also provide a potential explanation for why MCS scores were not impaired in former cricketers with persistent joint pain, despite impaired PCS scores. In a recent meta-analysis, former athletes with impaired PCS scores reported greater MCS scores compared to general population norms.[75] In a typical OA population, both PCS and MCS scores are impaired,[76] suggesting HRQoL in former sport participants with OA may differ from the typical OA population. Furthermore, the high PA levels in former cricketers in our study could also contribute to the high MCS scores. Physical activity has been shown to have a protective effect against depression and a positive impact on mental-components of HRQoL in the general population, older adults, and in former elite athletes.[77-80] Irrespective of joint pain, former cricketers reported MCS scores 5 to 6 points better than the population normative average, and this difference is likely to be clinically meaningful.[81]

Former cricketers with persistent joint pain had impaired physical-components of HRQoL compared to cricketers without persistent pain. This supports previous literature which found impaired PCS scores in former collegiate[82-84] and professional athletes[85] compared to non-athlete controls. Former cricketers with persistent pain reported PCS scores 5 to 7 points lower than former cricketers without persistent pain, demonstrating a clinical important difference between persistent pain and no pain groups. Impaired physical-components of HRQoL in former athletes has been attributed to poor joint health,[82-84] which may be due to previous musculoskeletal injury.[86-88] Former athletes with a history of musculoskeletal injury

For peer review only - http://bmjopen.bmj.com/site/about/guidelines.xhtml

report worse physical-components of HRQoL, than former athletes without a history of injury[86] and the general population.[87 88] In our study, former cricketers without persistent pain reported a median PCS score 4 points above the population normative mean of 50, suggesting that former cricket participants without joint pain have greater levels of physical functioning compared to the general population. Importantly, despite impaired PCS scores in former cricketers with persistent pain, MCS scores were not impaired. This highlights the importance of selecting a HRQoL measure that differentiates assessment of pain and physical function, from assessment of mental health and wellbeing, when evaluating HRQoL in former sport participants.

Cricketers with persistent lower extremity pain reported 3.1 points worse PCS scores compared to cricketers with upper extremity pain, and this difference is likely to be clinically meaningful.[81] Worse PCS scores in former cricketers with lower extremity pain may be due to greater difficulties performing usual physical activities (such as difficulties with ambulation and stair navigation[89 90]) or experiencing more severe bodily pain than those who report upper extremity pain. For example, former collegiate athletes with lower extremity persistent pain were 2.5 times more likely to be limited in physical function, such as stair navigation, and have greater impaired HRQoL, compared to non-athletes.[83] While there is research investigating the impacts of lower extremity pain on function in former athletes, our understanding of the impact of upper extremity pain on function in former athletes is poor. Further research could inform strategies to improve physical-components of HRQoL amongst former athletes with persistent upper extremity pain.

The relationship between persistent joint pain, PA, and HRQoL was similar between elite and recreational former cricketers. Few studies have investigated PA or HRQoL in former

For peer review only

For peer review only - http://bmjopen.bmj.com/site/about/guidelines.xhtml

athletes from different standards-of-play. Higher standards of sport competition requires elevated levels of resilience and psychological hardiness due to the increased levels of training and competition.[29-32 91] Despite the observed psychological strengths associated with elite cricket participation, any level of sport participation is associated with favourable psychological strengths such as resilience.[30 32 91] Thus, it is possible that all standards of cricket participation is associated with psychological benefits that may explain the high PA levels and mental-components of HRQoL in former cricketers living with persistant pain; further research is needed to explore this possibility.

Strengths and potential limitations

This study took into account the non-linear relationships between different continuous variables (i.e. by using fractional polynomials), where most studies assume linearity.[92] Missing data was low, allowing for low bias in a complete case analysis. Persistent joint pain was assessed by asking cricketers if they had joint pain on most days of the last month, which is based on the National health and Nutrition Examination Survey (NHANES). However, joint pain may occur intermittently, and still effect PA and HRQoL. As a result, this study may have missed participants with significant pain. Participants with persistent pain in both the upper and lower extremities, and participants with back pain were excluded from the analyses. This limits the scope of this study to participants with only upper or lower extremity persistent pain, or no persistent pain, decreasing the generalizability of these findings. The SF-8 minimal detectable difference and minimal clinical important difference literature is sparse, and does not evaluate athletic populations. This decreases the interpretability of these data.

Conclusions

For peer review only

Physical components of HRQoL were impaired in former cricketers with persistent joint pain compared to those without joint pain, and PCS scores were more impaired in those with lower extremity pain compared to those with upper extremity pain. However, former cricketers with persistent joint pain did not have reduced PA levels or mental components of HRQoL, compared to those with no persistent joint pain. These relationships were similar amongst former elite and recreational cricketers. These results highlight the need to select a HRQoL measure that differentiates assessment of pain and physical functional, from assessment of mental health and wellbeing, when evaluating HRQoL in former sport participants. Further research is needed to determine whether the psychological benefits of sport participation explain why former cricketers had high PA levels and mental-components of HRQoL, despite living with persistant joint-pain.

Author's Contributions

All authors were involved in planning, methodology design, and editing the manuscript. Garrett Bullock and Stephanie Filbay wrote the first draft of the manuscript. Garrett Bullock, Stephanie Filbay, and Gary Collins also contributed to statistical analyses.

Funding: This study was funded by Centre for Sport, Exercise and Osteoarthritis Research Versus Arthritis (grant reference 21595). Gary Collins was supported by the NIHR Biomedical Research Centre, Oxford.

For peer review only - http://bmjopen.bmj.com/site/about/guidelines.xhtml

References

1. Plotnikoff R, Karunamuni N, Lytvyak E, et al. Osteoarthritis prevalence and modifiable factors: a population study. *BMC public health* 2015;15:1195. doi: 10.1186/s12889-015-2529-0 [published Online First: 2015/12/02]
2. van Schoor NM, Zambon S, Castell MV, et al. Impact of clinical osteoarthritis of the hip, knee and hand on self-rated health in six European countries: the European Project on OSteoArthritis. *Quality of life research : an international journal of quality of life aspects of treatment, care and rehabilitation* 2016;25(6):1423-32. doi: 10.1007/s11136-015-1171-8 [published Online First: 2015/11/09]
3. Palazzo C, Nguyen C, Lefevre-Colau MM, et al. Risk factors and burden of osteoarthritis. *Annals of physical and rehabilitation medicine* 2016;59(3):134-38. doi: 10.1016/j.rehab.2016.01.006 [published Online First: 2016/02/26]
4. Cross M, Smith E, Hoy D, et al. The global burden of hip and knee osteoarthritis: estimates from the global burden of disease 2010 study. *Annals of the rheumatic diseases* 2014;73(7):1323-30. doi: 10.1136/annrheumdis-2013-204763 [published Online First: 2014/02/21]
5. Carbone A, Rodeo S. Review of current understanding of post-traumatic osteoarthritis resulting from sports injuries. *Journal of orthopaedic research : official publication of the Orthopaedic Research Society* 2017;35(3):397-405. doi: 10.1002/jor.23341 [published Online First: 2016/06/17]
6. Brown TD, Johnston RC, Saltzman CL, et al. Posttraumatic osteoarthritis: a first estimate of incidence, prevalence, and burden of disease. *Journal of orthopaedic trauma* 2006;20(10):739-44. doi: 10.1097/01.bot.0000246468.80635.ef [published Online First: 2006/11/16]
7. Vannini F, Spalding T, Andriolo L, et al. Sport and early osteoarthritis: the role of sport in aetiology, progression and treatment of knee osteoarthritis. *Knee surgery, sports traumatology, arthroscopy : official journal of the ESSKA* 2016;24(6):1786-96. doi: 10.1007/s00167-016-4090-5 [published Online First: 2016/04/05]
8. Whittaker JL, Woodhouse LJ, Nettel-Aguirre A, et al. Outcomes associated with early post-traumatic osteoarthritis and other negative health consequences 3-10 years following knee joint injury in youth sport. *Osteoarthritis and cartilage* 2015;23(7):1122-9. doi: 10.1016/j.joca.2015.02.021 [published Online First: 2015/03/01]
9. Neogi T. The epidemiology and impact of pain in osteoarthritis. *Osteoarthritis and cartilage* 2013;21(9):1145-53. doi: 10.1016/j.joca.2013.03.018 [published Online First: 2013/08/27]
10. Arliani GG, Astur DC, Yamada RK, et al. Early osteoarthritis and reduced quality of life after retirement in former professional soccer players. *Clinics (Sao Paulo, Brazil)* 2014;69(9):589-94. [published Online First: 2014/10/16]
11. Turner AP, Barlow JH, Heathcote-Elliott C. Long term health impact of playing professional football in the United Kingdom. *British journal of sports medicine* 2000;34(5):332-6. [published Online First: 2000/10/26]
12. Jones ME, Davies MAM, Leyland KM, et al. Osteoarthritis and other long-term health conditions in former elite cricketers. *Journal of science and medicine in sport* 2018;21(6):558-63. doi: 10.1016/j.jsams.2017.10.013 [published Online First: 2017/11/02]
13. Davies MAM, A DJ, Delmestri A, et al. Health amongst former rugby union players: A cross-sectional study of morbidity and health-related quality of life. *Scientific reports*

For peer review only - http://bmjopen.bmj.com/site/about/guidelines.xhtml

2017;7(1):11786. doi: 10.1038/s41598-017-12130-y [published Online First: 2017/09/30]

14. Spector TD, Harris PA, Hart DJ, et al. Risk of osteoarthritis associated with long-term weight-bearing sports: a radiologic survey of the hips and knees in female ex-athletes and population controls. *Arthritis and rheumatism* 1996;39(6):988-95. [published Online First: 1996/06/01]
15. Lang CE, Bland MD, Bailey RR, et al. Assessment of upper extremity impairment, function, and activity after stroke: foundations for clinical decision making. *Journal of hand therapy : official journal of the American Society of Hand Therapists* 2013;26(2):104-14;quiz 15. doi: 10.1016/j.jht.2012.06.005 [published Online First: 2012/09/15]
16. Shaw WS, Feuerstein M, Lincoln AE, et al. Ergonomic and psychosocial factors affect daily function in workers' compensation claimants with persistent upper extremity disorders. *Journal of occupational and environmental medicine* 2002;44(7):606-15. [published Online First: 2002/07/24]
17. Peters SE, Johnston V, Ross M, et al. Expert consensus on facilitators and barriers to return-to-work following surgery for non-traumatic upper extremity conditions: a Delphi study. *The Journal of hand surgery, European volume* 2017;42(2):127-36. doi: 10.1177/1753193416669263 [published Online First: 2016/09/23]
18. Hundza S, Quartly C, Kim JM, et al. Similar barriers and facilitators to physical activity across different clinical groups experiencing lower limb spasticity. *Disability and rehabilitation* 2016;38(14):1370-81. doi: 10.3109/09638288.2015.1101789 [published Online First: 2016/01/05]
19. Barnes KR, McGuigan MR, Kilding AE. Lower-body determinants of running economy in male and female distance runners. *Journal of strength and conditioning research* 2014;28(5):1289-97. doi: 10.1519/jsc.0000000000000267 [published Online First: 2013/10/16]
20. Vogt MT, Lauerman WC, Chirumbole M, et al. A community-based study of postmenopausal white women with back and leg pain: health status and limitations in physical activity. *The journals of gerontology Series A, Biological sciences and medical sciences* 2002;57(8):M544-50. [published Online First: 2002/07/30]
21. Montero A, Mulero JF, Tornero C, et al. Pain, disability and health-related quality of life in osteoarthritis-joint matters: an observational, multi-specialty trans-national follow-up study. *Clin Rheumatol* 2016;35(9):2293-305. doi: 10.1007/s10067-016-3248-3 [published Online First: 2016/04/14]
22. Verma C, Parikh R, Nadkar M, et al. Correlation of Functional Ability of the Hand with Upper Limb Function and Quality of Life in Patients with Rheumatoid Arthritis. *J Assoc Physicians India* 2017;65(3):20-24. [published Online First: 2017/05/04]
23. Rincon-Hurtado AM, Rocha-Buelvas A, Lopez-Cardona A, et al. Health-related quality of life of patients with rotator cuff injuries, Cofee Triangle, Colombia, 2013. *Rev Bras Ortop* 2018;53(3):364-72. doi: 10.1016/j.rboe.2018.03.018 [published Online First: 2018/06/13]
24. Cassou B, Derriennic F, Monfort C, et al. Chronic neck and shoulder pain, age, and working conditions: longitudinal results from a large random sample in France. *Occup Environ Med* 2002;59(8):537-44. [published Online First: 2002/08/02]
25. Grzywacz JG, Arcury TA, Mora D, et al. Work organization and musculoskeletal health: clinical findings from immigrant Latino poultry processing and other manual workers. *J Occup Environ Med* 2012;54(8):995-1001. doi: 10.1097/JOM.0b013e318254640d [published Online First: 2012/07/24]

For peer review only

For peer review only - http://bmjopen.bmj.com/site/about/guidelines.xhtml

26. Sauers EL, Dykstra DL, Bay RC, et al. Upper extremity injury history, current pain rating, and health-related quality of life in female softball pitchers. *Journal of sport rehabilitation* 2011;20(1):100-14. [published Online First: 2011/03/18]
27. Soomro N, Strasiotto L, Sawdagar T, et al. Cricket Injury Epidemiology in the Twenty-First Century: What is the Burden? *Sports medicine (Auckland, NZ)* 2018;48(10):2301-16. doi: 10.1007/s40279-018-0960-y [published Online First: 2018/07/19]
28. Posner M, Cameron KL, Wolf JM, et al. Epidemiology of Major League Baseball injuries. *The American journal of sports medicine* 2011;39(8):1676-80. doi: 10.1177/0363546511411700 [published Online First: 2011/06/29]
29. Cowden RG, Fuller DK, Anshel MH. Psychological predictors of mental toughness in elite tennis: an exploratory study in learned resourcefulness and competitive trait anxiety. *Perceptual and motor skills* 2014;119(3):661-78. doi: 10.2466/30.PMS.119c27z0 [published Online First: 2014/11/12]
30. Secades XG, Molinero O, Salguero A, et al. Relationship Between Resilience and Coping Strategies in Competitive Sport. *Perceptual and motor skills* 2016;122(1):336-49. doi: 10.1177/0031512516631056 [published Online First: 2016/07/16]
31. Shields MR, Brooks MA, Koltyn KF, et al. Cognitive Resilience and Psychological Responses across a Collegiate Rowing Season. *Medicine and science in sports and exercise* 2017;49(11):2276-85. doi: 10.1249/mss.0000000000001363 [published Online First: 2017/07/07]
32. Sarkar M, Fletcher D. Psychological resilience in sport performers: a review of stressors and protective factors. *Journal of sports sciences* 2014;32(15):1419-34. doi: 10.1080/02640414.2014.901551 [published Online First: 2014/04/11]
33. Wermelinger Avila MP, Correa JC, Lucchetti ALG, et al. The Role of Physical Activity in the Association Between Resilience and Mental Health in Older Adults. *Journal of aging and physical activity* 2018;26(2):248-53. doi: 10.1123/japa.2016-0332 [published Online First: 2017/07/18]
34. Taylor K, Scruggs PW, Balemba OB, et al. Associations between physical activity, resilience, and quality of life in people with inflammatory bowel disease. *European journal of applied physiology* 2018;118(4):829-36. doi: 10.1007/s00421-018-3817-z [published Online First: 2018/02/08]
35. Seaton CL, Bottorff JL, Jones-Bricker M, et al. The Role of Positive Emotion and Ego-Resilience in Determining Men's Physical Activity Following a Workplace Health Intervention. *American journal of men's health* 2018;12(6):1916-28. doi: 10.1177/1557988318803744 [published Online First: 2018/10/20]
36. Paffenbarger RS, Jr., Lee IM, Leung R. Physical activity and personal characteristics associated with depression and suicide in American college men. *Acta psychiatrica Scandinavica Supplementum* 1994;377:16-22. [published Online First: 1994/01/01]
37. Camacho TC, Roberts RE, Lazarus NB, et al. Physical activity and depression: evidence from the Alameda County Study. *American journal of epidemiology* 1991;134(2):220-31. [published Online First: 1991/07/15]
38. Farmer ME, Locke BZ, Moscicki EK, et al. Physical activity and depressive symptoms: the NHANES I Epidemiologic Follow-up Study. *American journal of epidemiology* 1988;128(6):1340-51. [published Online First: 1988/12/01]
39. Igolnikov I, Gallagher RM, Hainline B. Sport-related injury and pain classification. *Handbook of clinical neurology* 2018;158:423-30. doi: 10.1016/b978-0-444-63954-7.00039-2 [published Online First: 2018/11/30]
40. Harris PA, Taylor R, Thielke R, et al. Research electronic data capture (REDCap)--a metadata-driven methodology and workflow process for providing translational

For peer review only

For peer review only - http://bmjopen.bmj.com/site/about/guidelines.xhtml

research informatics support. *Journal of biomedical informatics* 2009;42(2):377-81. doi: 10.1016/j.jbi.2008.08.010 [published Online First: 2008/10/22]

41. Lyon JA, Garcia-Milian R, Norton HF, et al. The use of Research Electronic Data Capture (REDCap) software to create a database of librarian-mediated literature searches. *Medical reference services quarterly* 2014;33(3):241-52. doi: 10.1080/02763869.2014.925379 [published Online First: 2014/07/16]
42. Committee IR. Guidelines for data processing and analysis of the International Physical Activity Questionnaire (IPAQ)-short and long forms. *http://www ipaq ki se/scoring pdf* 2005
43. Rosemann T, Kuehlein T, Laux G, et al. Osteoarthritis of the knee and hip: a comparison of factors associated with physical activity. *Clinical rheumatology* 2007;26(11):1811-7. doi: 10.1007/s10067-007-0579-0 [published Online First: 2007/03/03]
44. Hultenheim Klintberg I, Karlsson J, Svantesson U. Health-related quality of life, patient satisfaction, and physical activity 8-11 years after arthroscopic subacromial decompression. *Journal of shoulder and elbow surgery* 2011;20(4):598-608. doi: 10.1016/j.jse.2010.08.021 [published Online First: 2010/12/15]
45. Ekelund U, Sepp H, Brage S, et al. Criterion-related validity of the last 7-day, short form of the International Physical Activity Questionnaire in Swedish adults. *Public health nutrition* 2006;9(2):258-65.
46. Mader U, Martin BW, Schutz Y, et al. Validity of four short physical activity questionnaires in middle-aged persons. *Medicine and science in sports and exercise* 2006;38(7):1255-66. doi: 10.1249/01.mss.0000227310.18902.28 [published Online First: 2006/07/11]
47. Macfarlane DJ, Lee CC, Ho EY, et al. Convergent validity of six methods to assess physical activity in daily life. *Journal of applied physiology (Bethesda, Md : 1985)* 2006;101(5):1328-34. doi: 10.1152/japplphysiol.00336.2006 [published Online First: 2006/07/11]
48. Turner-Bowker DM, Bayliss MS, Ware JE, Jr., et al. Usefulness of the SF-8 Health Survey for comparing the impact of migraine and other conditions. *Quality of life research : an international journal of quality of life aspects of treatment, care and rehabilitation* 2003;12(8):1003-12. [published Online First: 2003/12/04]
49. Hays RD, Sherbourne CD, Mazel RM. The RAND 36-Item Health Survey 1.0. *Health economics* 1993;2(3):217-27. [published Online First: 1993/10/01]
50. Ware JE, Jr., Sherbourne CD. The MOS 36-item short-form health survey (SF-36). I. Conceptual framework and item selection. *Medical care* 1992;30(6):473-83. [published Online First: 1992/06/11]
51. Ware JE, Kosinski M, Dewey JE, et al. How to score and interpret single-item health status measures: a manual for users of the SF-8 health survey. *Lincoln, RI: QualityMetric Incorporated* 2001;15(10):5.
52. Angst F, Aeschlimann A, Stucki G. Smallest detectable and minimal clinically important differences of rehabilitation intervention with their implications for required sample sizes using WOMAC and SF-36 quality of life measurement instruments in patients with osteoarthritis of the lower extremities. *Arthritis Care & Research: Official Journal of the American College of Rheumatology* 2001;45(4):384-91.
53. Samsa G, Edelman D, Rothman ML, et al. Determining clinically important differences in health status measures. *Pharmacoeconomics* 1999;15(2):141-55.
54. Mols F, Vingerhoets AJ, Coebergh JW, et al. Quality of life among long-term breast cancer survivors: a systematic review. *European journal of cancer* 2005;41(17):2613-19.

For peer review only

For peer review only - http://bmjopen.bmj.com/site/about/guidelines.xhtml

55. Doyle C, Kushi LH, Byers T, et al. Nutrition and physical activity during and after cancer treatment: an American Cancer Society guide for informed choices. *CA: a cancer journal for clinicians* 2006;56(6):323-53.
56. Manson JE, Stampfer M, Colditz G, et al. Physical activity and incidence of non-insulin-dependent diabetes mellitus in women. *The Lancet* 1991;338(8770):774-78.
57. Goldney RD, Phillips PJ, Fisher LJ, et al. Diabetes, depression, and quality of life: a population study. *Diabetes care* 2004;27(5):1066-70.
58. Moore SA, Hallsworth K, Plötz T, et al. Physical activity, sedentary behaviour and metabolic control following stroke: a cross-sectional and longitudinal study. *PloS one* 2013;8(1):e55263.
59. Royston P, Altman DG. Regression using fractional polynomials of continuous covariates: parsimonious parametric modelling. *Journal of the Royal Statistical Society: Series C (Applied Statistics)* 1994;43(3):429-53.
60. Hadley Wickham RF, Lionel Henry, Kirill Muller. dplyr: A Grammar of Data Manipulation 2018 [R package version 0.7.6.:[Available from: https://CRAN.R-project.org/package=dplyr.
61. Nicholas Tierney DC, Miles McBain, Colin Fay, Mitchell O'Hara-Wild, Jim Hester, Luke Smith. naniar 2018 [R Package version: 0.4.1:[Available from: https://cran.r-project.org/web/packages/naniar/index.html.
62. Gareth Ambler AB. mfp. R package 1.5.2 ed2015.
63. Wallis JA, Webster KE, Levinger P, et al. What proportion of people with hip and knee osteoarthritis meet physical activity guidelines? A systematic review and meta-analysis. *Osteoarthritis and cartilage* 2013;21(11):1648-59. doi: 10.1016/j.joca.2013.08.003 [published Online First: 2013/08/21]
64. Aadahl M, Kjaer M, Jorgensen T. Associations between overall physical activity level and cardiovascular risk factors in an adult population. *European journal of epidemiology* 2007;22(6):369-78. doi: 10.1007/s10654-006-9100-3 [published Online First: 2007/03/03]
65. Schaller N, Seiler H, Himmerich S, et al. Estimated physical activity in Bavaria, Germany, and its implications for obesity risk: results from the BVS-II Study. *International Journal of Behavioral Nutrition and Physical Activity* 2005;2(1):6.
66. Norman A, Bellocco R, Bergström A, et al. Validity and reproducibility of self-reported total physical activity—differences by relative weight. *International journal of obesity* 2001;25(5):682.
67. Holden MA, Nicholls EE, Young J, et al. Exercise and physical activity in older adults with knee pain: a mixed methods study. *Rheumatology (Oxford, England)* 2015;54(3):413-23. doi: 10.1093/rheumatology/keu333 [published Online First: 2014/09/05]
68. Filbay SR, Bishop FL, Peirce N, et al. Physical activity in former elite cricketers and strategies for promoting physical activity after retirement from cricket: a qualitative study. *BMJ open* 2017;7(11):e017785. doi: 10.1136/bmjopen-2017-017785 [published Online First: 2017/11/21]
69. Teixeira PJ, Carraça EV, Markland D, et al. Exercise, physical activity, and self-determination theory: a systematic review. *International journal of behavioral nutrition and physical activity* 2012;9(1):78.
70. Filbay SR, Bishop F, Peirce N, et al. Common attributes in retired professional cricketers that may enhance or hinder quality of life after retirement: a qualitative study. *BMJ open* 2017;7(7):e016541. doi: 10.1136/bmjopen-2017-016541 [published Online First: 2017/07/29]

For peer review only

For peer review only - http://bmjopen.bmj.com/site/about/guidelines.xhtml

71. Hammond LE, Lilley JM, Pope GD, et al. 'We've just learnt to put up with it': an exploration of attitudes and decision-making surrounding playing with injury in English professional football. *Qualitative Research in Sport, Exercise and Health* 2014;6(2):161-81.
72. Roderick M, Waddington I, Parker G. Playing hurt: Managing injuries in English professional football. *International review for the sociology of sport* 2000;35(2):165-80.
73. Meggs J, Ditzfeld C, Golby J. Self-concept organisation and mental toughness in sport. *Journal of sports sciences* 2014;32(2):101-9. doi: 10.1080/02640414.2013.812230 [published Online First: 2013/08/24]
74. Nicholls AR, Polman RCJ, Levy AR, et al. Mental toughness, optimism, pessimism, and coping among athletes. *Personality and Individual Differences* 2008;44(5):1182-92. doi: https://doi.org/10.1016/j.paid.2007.11.011
75. FIlbay SR PT, Thomas B, McKay C, Adams J, Arden NK. Quality of life and life satisfaction in former athletes: a systematic review and meta-analysis

. *Sports Medicine* 2019
76. Kobayashi S, Pappas E, Fransen M, et al. The prevalence of patellofemoral osteoarthritis: a systematic review and meta-analysis. *Osteoarthritis and cartilage* 2016;24(10):1697-707. doi: 10.1016/j.joca.2016.05.011 [published Online First: 2016/05/18]
77. Harvey SB, Overland S, Hatch SL, et al. Exercise and the Prevention of Depression: Results of the HUNT Cohort Study. *The American journal of psychiatry* 2018;175(1):28-36. doi: 10.1176/appi.ajp.2017.16111223 [published Online First: 2017/10/04]
78. Salmon P. Effects of physical exercise on anxiety, depression, and sensitivity to stress: a unifying theory. *Clinical psychology review* 2001;21(1):33-61. [published Online First: 2001/01/10]
79. Vasiliadis HM, Belanger MF. The prospective and concurrent effect of exercise on health related quality of life in older adults over a 3 year period. *Health and quality of life outcomes* 2018;16(1):15. doi: 10.1186/s12955-018-0843-9 [published Online First: 2018/01/18]
80. Bäckmand H, Kaprio J, Kujala U, et al. Influence of physical activity on depression and anxiety of former elite athletes. *International journal of sports medicine* 2003;24(08):609-19.
81. Hays RD, Morales LS. The RAND-36 measure of health-related quality of life. *Annals of medicine* 2001;33(5):350-57.
82. Simon JE, Docherty CL. Current Health-Related Quality of Life in Former National Collegiate Athletic Association Division I Collision Athletes Compared With Contact and Limited-Contact Athletes. *Journal of athletic training* 2016;51(3):205-12. doi: 10.4085/1062-6050-51.4.05 [published Online First: 2016/03/10]
83. Simon JE, Docherty CL. Current health-related quality of life is lower in former Division I collegiate athletes than in non-collegiate athletes. *The American journal of sports medicine* 2014;42(2):423-9. doi: 10.1177/0363546513510393 [published Online First: 2013/12/10]
84. Sorenson SC, Romano R, Scholefield RM, et al. Holistic life-span health outcomes among elite intercollegiate student-athletes. *Journal of athletic training* 2014;49(5):684-95. doi: 10.4085/1062-6050-49.3.18 [published Online First: 2014/08/15]

For peer review only - http://bmjopen.bmj.com/site/about/guidelines.xhtml

85. Gouttebarge V, Aoki H, Kerkhoffs G. Lower extremity osteoarthritis is associated with lower health-related quality of life among retired professional footballers. *The Physician and sportsmedicine* 2018;46(4):471-76. doi: 10.1080/00913847.2018.1451718 [published Online First: 2018/03/13]
86. Cameron KL, Thompson BS, Peck KY, et al. Normative values for the KOOS and WOMAC in a young athletic population: history of knee ligament injury is associated with lower scores. *The American journal of sports medicine* 2013;41(3):582-9. doi: 10.1177/0363546512472330 [published Online First: 2013/01/19]
87. McAllister DR, Motamedi AR, Hame SL, et al. Quality of life assessment in elite collegiate athletes. *The American journal of sports medicine* 2001;29(6):806-10. doi: 10.1177/03635465010290062201 [published Online First: 2001/12/06]
88. Valovich McLeod TC, Decoster LC, Loud KJ, et al. National Athletic Trainers' Association position statement: prevention of pediatric overuse injuries. *Journal of athletic training* 2011;46(2):206-20. doi: 10.4085/1062-6050-46.2.206 [published Online First: 2011/03/12]
89. Gardner AW, Montgomery PS, Wang M, et al. Predictors of health-related quality of life in patients with symptomatic peripheral artery disease. *Journal of vascular surgery* 2018;68(4):1126-34. doi: 10.1016/j.jvs.2017.12.074 [published Online First: 2018/04/05]
90. Zhao Y, Chung PK. Neighborhood environment walkability and health-related quality of life among older adults in Hong Kong. *Archives of gerontology and geriatrics* 2017;73:182-86. doi: 10.1016/j.archger.2017.08.003 [published Online First: 2017/08/22]
91. Ninkovic S, Avramov S, Harhaji V, et al. INFLUENCE OF DIFFERENT LEVELS OF SPORTS ACTIVITIES ON THE QUALITY OF LIFE AFTER THE RECONSTRUCTION OF ANTERIOR CRUCIATE LIGAMENT. *Medicinski pregled* 2015;68(3-4):116-21. [published Online First: 2015/07/29]
92. Royston P, Sauerbrei W. Building multivariable regression models with continuous covariates in clinical epidemiology. *Methods of information in medicine* 2005;44(04):561-71.

For peer review only

For peer review only - http://bmjopen.bmj.com/site/about/guidelines.xhtml

FIGURE 1. Study Flow Chart

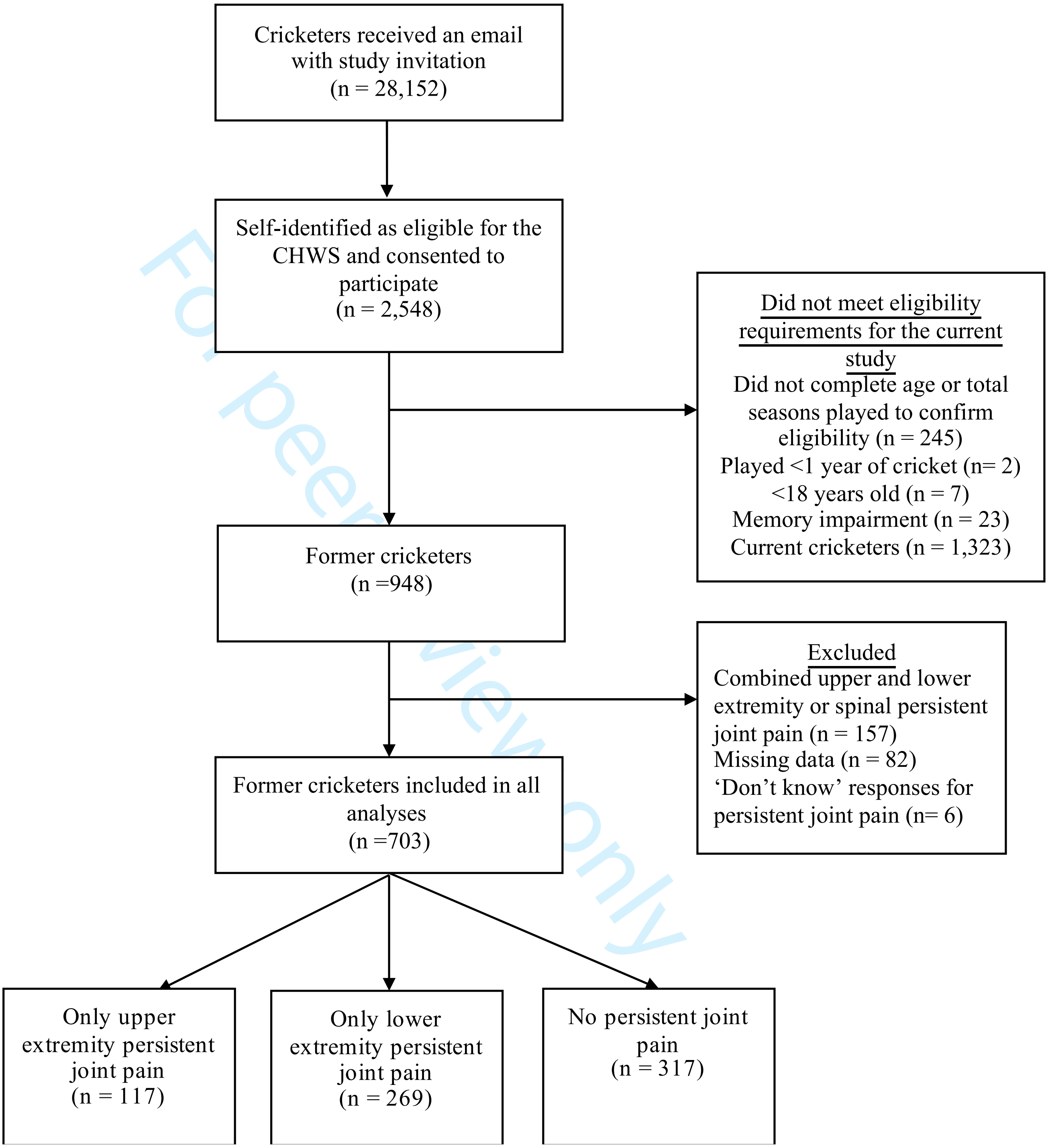

For peer review only

Figure 2: Weekly physical activity levels and health related quality of life in former cricketers with upper extremity persistent joint pain, lower extremity persistent joint pain, or no persistent joint pain

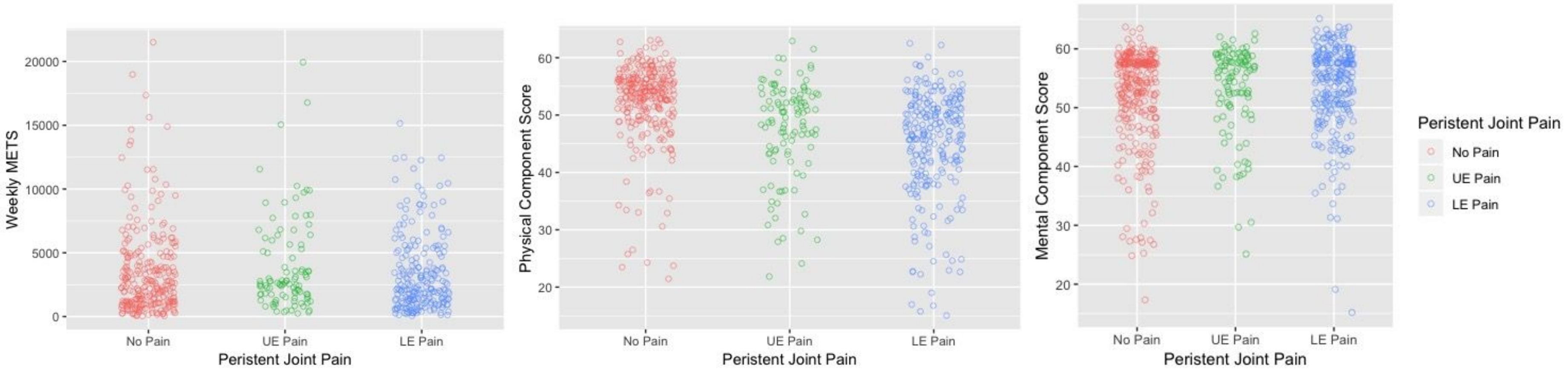

METS = Metabolic Equivalents, PCS = Physical Component Score, MCS = Mental Component Score, UE= Upper Extremity, LE=Lower Extremity

TABLE 1. Participant characteristics

	All former cricketers (n = 703)	Upper extremity persistent joint pain (n = 117)	Lower extremity persistent joint pain (n = 269)	No persistent joint pain (n = 317)
Age (years)	58.7 (SD 12.9)	59.9 (SD 12.6)	59.8 (SD 11.8)	57.8 (SD 13.8)
Sex				
Male	683 (97%)	113 (97%)	263 (98%)	307 (97%)
Female	15 (3%)	3 (3%)	4 (2%)	8 (3%)
NR	5 (<1%)	1 (<1%)	2 (<1%)	2 (<1%)
BMI (kg/m2)	28.3 (SD 5.3)	27.9 (SD 5.0)	29.1 (SD 5.5)	27.8 (SD 5.4)
Seasons Played	30 (IQR 20-40)	35 (IQR 27-44)	32 (IQR 23-42)	30 (IQR 18-48)
Years since last cricket match	7.4 (IQR 0.4-14.4)	8.1 (IQR 0.8-15.4)	6.9 (IQR 0.1-13.7)	6.7 (IQR 0.1-13.3)
Playing standard				
Elite	264 (38%)	46 (39%)	109 (41%)	109 (34%)
Recreational	428 (61%)	68 (58%)	156 (58%)	204 (65%)
NR	11 (1%)	3 (3%)	4 (1%)	4 (1%)
Comorbidity				
No	497 (73%)	81 (74%)	186 (71%)	230 (75%)
Yes	155 (23%)	23 (21%)	64 (25%)	68 (22%)
NR	27 (4%)	6 (5%)	10 (4%)	11 (4%)

SD = Standard Deviation, IQR = Inter Quartile Range, NR= No Response, BMI = Body Mass Index, [a]Participants reported the highest standard they had played for at least one season and then were stratified into recreational (university, school, village or social) and elite (international or county/premier league, academy or county age group)

For peer review only

TABLE 2. The relationship between persistent joint pain, physical activity and health related quality of life

	Weekly METS		PCS score		MCS score	
	Unadjusted Effect[b] (95% CI)	Adjusted[a] Effect[b] (95% CI)	Unadjusted Effect[c] (95% CI)	Adjusted[a] Effect[c] (95% CI)	Unadjusted Effect[c] (95% CI)	Adjusted[a] Effect[c] (95% CI)
Upper extremity persistent joint pain	20.3 (-3.2, 117), p=0.83	28.1 (-1.1, 136), p=0.10	-5.4 (-7.3, -3.6), p<0.0001	-5.5 (-7.4, -3.5), p<0.0001	-0.1 (-0.2, 0.1), p=0.35	-0.1 (-0.2, 0.1), p=0.26
Lower extremity persistent joint pain	0.3 (-12.5, 21.0), p=0.16	4.6 (-7.5, 49.4), p=0.39	-6.9 (-8.4, -5.5), p<0.0001	-6.6 (-8.1, -5.2), p<0.0001	0.09 (-0.2, 0.1), p=0.08	-0.1 (-0.2, 0.1), p=0.06
No persistent joint pain	*Reference group*		*Reference group*		*Reference group*	

[a] Estimates are adjusted for age, body mass index, and comorbidities
[b] Participants with memory impairments were excluded from the analyses
[c] Comorbidities were defined as none present (0) and presence of at least one comorbidity (1). Comorbidities included were diabetes, stroke, skin cancer, and other cancer
[d] Upper extremity (shoulder, elbow, wrist, hand) and lower extremity (hip, knee, ankle) persistent joint pain were assessed by asking individuals if they had joint-specific pain on 'most days of the last month.'
[e] Questionnaire Short-Form (IPAQ-SF); Physical activity was calculated as metabolic equivalents (METS) per week; METS were transformed prior to analysis by taking the square root and then retransformed by squaring after analysis
[f] SF-8: Short-Form 8 Health Survey; Physical Component Scores (PCS) and Mental Component Scores (MCS) were calculated using norm based scoring (population norm 50 SD 10, high scorer = better health-related quality of life)

For peer review only

TABLE 3. The relationship between persistent joint pain, physical activity and health related quality of life, in former recreational and elite cricketer subgroups

		Weekly METS		PCS score		MCS score	
		Unadjusted Effect[b] (95% CI)	Adjusted[a] Effect[b] (95% CI)	Unadjusted Effect[c] (95% CI)	Adjusted[a] Effect[c] (95% CI)	Unadjusted Effect[c] (95% CI)	Adjusted[a] Effect[c] (95% CI)
Former elite cricketers (n=280)	Upper extremity persistent joint pain	70 (-1.6, 324), p=0.09	88 (-0.1, 356), p=0.05	-5.3 (-8.6, -2.0), p=0.001	-5.3 (-8.8, -1.9), p=0.002	0.055 (-2.3, 3.4), p=0.70	5.3 (-1.1, 11.7), p=0.10
	Lower extremity persistent joint pain	5.2 (-28, 97), p=0.56	8.2 (-21, 106), p=0.45	-7.5 (-10.1, -4.9), p<0.0001	-7.0 (-9.7, -4.3), p<0.0001	0.1 (-1.7, 2.7), p=0.64	2.2 (-2.7, 7.0), p=0.39
	No persistent joint pain	*Reference group*		*Reference group*		*Reference group*	
Former recreational cricketers (n=423)		Unadjusted Effect[b] (95% CI)	Adjusted[a] Effect[b] (95% CI)	Unadjusted Effect[c] (95% CI)	Adjusted[a] Effect[c] (95% CI)	Unadjusted Effect[c] (95% CI)	Adjusted[a] Effect[c] (95% CI)
	Upper extremity persistent joint pain	5.6 (-37, 117), p=0.58	10.5 (-29, 149), p=0.47	-5.6 (-7.9, -3.3), p<0.0001	-5.8 (-8.1, -3.4), p<0.0001	0.8 (-1.5, 3.0), p=0.50	1.1 (-1.1, 3.2), p=0.35
	Lower extremity persistent joint pain	-0.7 (-49, 29), p=0.83	1.8 (-26, 61), p=0.68	-6.6 (-8.3, -4.9), p<0.0001	-6.2 (-8.0, -4.5), p<0.0001	-0.8 (-1.1, 2.7), p=0.33	1.2 (-0.5, 2.8), p=0.17

For peer review only

No persistent joint pain	*Reference group*	*Reference group*	*Reference group*

[a] Estimates are adjusted for age, body mass index, and comorbidities
[b] Participants with memory impairments were excluded from the analyses
[c] Comorbidities were defined as not present (0) and presence of at least one comorbidity (1). Comorbidities included were diabetes, stroke, skin cancer, and other cancer
[e] Upper extremity (shoulder, elbow, wrist, or hand) and lower extremity (hip, knee, or ankle) persistent joint pain were assessed by asking individuals if they had joint-specific pain on 'most days of the last month.'
[f] Questionnaire Short-Form (IPAQ-SF); Physical activity was calculated as metabolic equivalents (METS) per week; METS were transformed prior to analysis by taking the square root and then retransformed by squaring after analysis
[g] SF-8: Short-Form 8 Health Survey; Physical Component Scores (PCS) were calculated using norm based scoring (population norm 50 SD 10, high scorer = better health-related quality of life); Mental Component Scores (MCS) were calculated using norm based scoring (population norm 50 SD 10, high scorer = better health-related quality of life)

For peer review only

Appendix 1. Missing Data Responses in Former Cricketers

Variable	Count	Percentage
Physical Component Score	82	8.6%
Mental Component Score	80	8.4%
Body mass Index	61	6.3%
IPAQ-SF	56	5.9%
Cricket Seasons	12	1.7%
Persistent Pain	10	1.4%
Osteoarthritis	10	1.4%
Other Cancer	10	1.4%
Skin Cancer	8	1.1%
Stroke	8	1.1%
Diabetes	1	0.1%
Age	0	0%

For peer review only

STROBE Statement—checklist of items that should be included in reports of observational studies

	Item No	Recommendation
**Title and abstract**	1	(*a*) Indicate the study's design with a commonly used term in the title or the abstract **Page 1 and 3**
		(*b*) Provide in the abstract an informative and balanced summary of what was done and what was found **page 3**
**Introduction**		
Background/rationale	2	Explain the scientific background and rationale for the investigation being reported **Page 4**
Objectives	3	State specific objectives, including any prespecified hypotheses **page 5**
**Methods**		
Study design	4	Present key elements of study design early in the paper **page 5-6**
Setting	5	Describe the setting, locations, and relevant dates, including periods of recruitment, exposure, follow-up, and data collection **page 6**
Participants	6	(*a*) *Cohort study*—Give the eligibility criteria, and the sources and methods of selection of participants. Describe methods of follow-up<br>*Case-control study*—Give the eligibility criteria, and the sources and methods of case ascertainment and control selection. Give the rationale for the choice of cases and controls<br>*Cross-sectional study*—Give the eligibility criteria, and the sources and methods of selection of participants **page 5**
		(*b*) *Cohort study*—For matched studies, give matching criteria and number of exposed and unexposed<br>*Case-control study*—For matched studies, give matching criteria and the number of controls per case
Variables	7	Clearly define all outcomes, exposures, predictors, potential confounders, and effect modifiers. Give diagnostic criteria, if applicable **page 6-8**
Data sources/ measurement	8*	For each variable of interest, give sources of data and details of methods of assessment (measurement). Describe comparability of assessment methods if there is more than one group **page 7-8**
Bias	9	Describe any efforts to address potential sources of bias **page 8**
Study size	10	Explain how the study size was arrived at **figure 1**
Quantitative variables	11	Explain how quantitative variables were handled in the analyses. If applicable, describe which groupings were chosen and why **page 8**
Statistical methods	12	(*a*) Describe all statistical methods, including those used to control for confounding **page 8**
		(*b*) Describe any methods used to examine subgroups and interactions **page 8**
		(*c*) Explain how missing data were addressed **page 8-9**
		(*d*) *Cohort study*—If applicable, explain how loss to follow-up was addressed<br>*Case-control study*—If applicable, explain how matching of cases and controls was addressed<br>*Cross-sectional study*—If applicable, describe analytical methods taking account of sampling strategy **page 4-5**
		(*e*) Describe any sensitivity analyses 8-9

Continued on next page

For peer review only - http://bmjopen.bmj.com/site/about/guidelines.xhtml

**Results**		
Participants	13*	(a) Report numbers of individuals at each stage of study—eg numbers potentially eligible, examined for eligibility, confirmed eligible, included in the study, completing follow-up, and analysed **Figure 1**
		(b) Give reasons for non-participation at each stage **Figure 1**
		(c) Consider use of a flow diagram **Figure 1**
Descriptive data	14*	(a) Give characteristics of study participants (eg demographic, clinical, social) and information on exposures and potential confounders **Table 1**
		(b) Indicate number of participants with missing data for each variable of interest **Appendix 1**
		(c) *Cohort study*—Summarise follow-up time (eg, average and total amount)
Outcome data	15*	*Cohort study*—Report numbers of outcome events or summary measures over time
		Case-control study—Report numbers in each exposure category, or summary measures of exposure
		Cross-sectional study—Report numbers of outcome events or summary measures **page 10-11, Table 1**
Main results	16	(*a*) Give unadjusted estimates and, if applicable, confounder-adjusted estimates and their precision (eg, 95% confidence interval). Make clear which confounders were adjusted for and why they were included **page 10-11, Table 2-3 and figure 2**
		(*b*) Report category boundaries when continuous variables were categorized **page 10-11, Table 2, 3**
		(*c*) If relevant, consider translating estimates of relative risk into absolute risk for a meaningful time period **NA for these analyses**
Other analyses	17	Report other analyses done—eg analyses of subgroups and interactions, and sensitivity analyses 9-11
**Discussion**		
Key results	18	Summarise key results with reference to study objectives **page 12**
Limitations	19	Discuss limitations of the study, taking into account sources of potential bias or imprecision. Discuss both direction and magnitude of any potential bias **page 15**
Interpretation	20	Give a cautious overall interpretation of results considering objectives, limitations, multiplicity of analyses, results from similar studies, and other relevant evidence **page 16**
Generalisability	21	Discuss the generalisability (external validity) of the study results **page 15-16**
**Other information**		
Funding	22	Give the source of funding and the role of the funders for the present study and, if applicable, for the original study on which the present article is based **page 16**

*Give information separately for cases and controls in case-control studies and, if applicable, for exposed and unexposed groups in cohort and cross-sectional studies.

Note: An Explanation and Elaboration article discusses each checklist item and gives methodological background and published examples of transparent reporting. The STROBE checklist is best used in conjunction with this article (freely available on the Web sites of PLoS Medicine at http://www.plosmedicine.org/, Annals of Internal Medicine at http://www.annals.org/, and Epidemiology at http://www.epidem.com/). Information on the STROBE Initiative is available at www.strobe-statement.org.

For peer review only - http://bmjopen.bmj.com/site/about/guidelines.xhtml

Physical activity and health related quality of life in former elite and recreational cricketers with upper extremity or lower extremity persistent joint pain

Journal:	*BMJ Open*
Manuscript ID	bmjopen-2019-032606.R1
Article Type:	Original research
Date Submitted by the Author:	05-Sep-2019
Complete List of Authors:	Bullock, Garrett; University of Oxford, Nuffield Department of Orthopaedics, Rheumatology, and Musculoskeletal Sciences<br>Collins, Gary; University of Oxford, Centre for Statistics in Medicine<br>Peirce, Nicholas; Nottingham University Hospitals Trust, Centre For Sports Medicine; England and Wales Cricket Board, National Cricket Performance Centre<br>Arden, Nigel; Nuffield Department of Orthopaedics, ;<br>Filbay, Stephanie; University of Oxford, Arthritis Research UK Centre for Sport, Exercise & Osteoarthritis, Nuffield Department of Orthopaedics, Rheumatology & Musculoskeletal Sciences
**Primary Subject Heading**:	Sports and exercise medicine
Secondary Subject Heading:	Mental health, Public health
Keywords:	Shoulder < ORTHOPAEDIC & TRAUMA SURGERY, Knee < ORTHOPAEDIC & TRAUMA SURGERY, ankle, Hip < ORTHOPAEDIC & TRAUMA SURGERY, hand, retired athlete

Physical activity and health related quality of life in former elite and recreational cricketers with upper extremity or lower extremity persistent joint pain

Garrett S. Bullock PT, DPT,[1, 2] Gary S. Collins Bsc (Hons), PhD,[3,4] Peirce N., BMBS, FRIS,[5, 6] Nigel K. Arden, MD, FRCP,[1, 2] Stephanie R. Filbay, B.Phty (Hons), PhD[1, 2]

1. Centre for Sport, Exercise and Osteoarthritis Research *Versus Arthritis,* University of Oxford, United Kingdom
2. Nuffield Department of Orthopaedics, Rheumatology, and Musculoskeletal Sciences, University of Oxford, Oxford, United Kingdom
3. Centre for Statistics in Medicine, Nuffield Department of Orthopaedics, Rheumatology, and Musculoskeletal Sciences, University of Oxford, Oxford United Kingdom
4. Oxford University Hospitals NHS Foundation Trust, Oxford, United Kingdom
5. England and Wales Cricket Board, National Cricket Performance Centre, Loughborough University, LE11 3TU, United Kingdom
6. National Centre for Sport and Exercise Medicine, Loughborough University, LE11 3TU, United Kingdom

Corresponding Author Information

Garrett S. Bullock PT, DPT
Nuffield Department of Orthopaedics, Rheumatology, and Musculoskeletal Sciences
University of Oxford
B4495
Oxford, United Kingdom
OX3 7LD
(865) 227-374
garrett.bullock@wolfson.ox.ac.uk

Key Words: Shoulder, Knee, Ankle, Hip, Hand, Sport, Retired Athletes

Word Count: 3518

Conflict of Interest: Dr. Nick Peirce is affiliated with the England and Wales Cricket Board. All other authors affirm that they have no involvement with any commercial organization that has a direct financial interest in any matter included in this manuscript.

For peer review only - http://bmjopen.bmj.com/site/about/guidelines.xhtml

Abstract

Objectives: Evaluate and compare physical activity (PA) and health-related quality-of-life (HRQoL) in former elite and recreational cricketers with upper extremity (UE), lower extremity (LE), or no joint pain.

Study Design: Cross-sectional cohort

Setting: Despite the high prevalence of joint pain in former athletes, the impact of UE-pain and LE-pain on PA and HRQoL, and potential differences between former recreational and elite athletes, is poorly understood.

Participants: 703 former cricketers aged ≥18 years (aged mean 58.7 SD 12.9, played an average 30 IQR 20-40 seasons, 72% had played at a recreational level) were recruited through the Cricket Health and Wellbeing Study and met eligibility requirements (UE-pain, LE-pain or no joint pain (defined as pain on most days of the past month)).

Primary and Secondary Outcomes: A questionnaire collected IPAQ-SF (weekly metabolic equivalents (METS)) and SF-8 (physical (PCS) and mental (MCS) component scores). Kruskall-Wallis tests with Dunn's post-hoc and multivariable linear regressions were performed.

Results: Weekly METS were similar in former cricketers with UE-pain (median(IQR) 2560(722-4398)), LE-pain (2215(527-3903)) and no pain (2449(695-4203), p=0.39). MCS scores were similar between groups (UE-pain 56.0(52.1-60.0); LE-pain 55.2(51.1-59.4); no pain 54.7(50.7-58.7), p=0.38). PCS scores were more impaired in former cricketers with UE-pain (49.8(44.9-54.8)) or LE-pain (46.7(41.0-51.9)) compared to no pain (54.2(51.5-56.9), p<0.0001). Former cricketers with LE-pain reported worse PCS scores than those with UE-pain (p=0.04). Similar relationships were observed in former elite and recreational cricketers.

Conclusion: Despite impaired physical-components of HRQoL in former cricketers with UE-pain or LE-pain, pain was not related to PA levels or mental-components of HRQoL. Physical-components of HRQoL was most impaired in those with LE-pain, and findings were similar amongst former elite and recreational cricketers.

Key Words: Shoulder, Knee, Ankle, Hip, Hand, Sport, Retired Athletes

Strengths and Limitations:

- This study took into account the non-linear relationships between different continuous variables (i.e. by using fractional polynomials), where most studies assume linearity.
- Missing data was low, allowing for low bias in a complete case analysis.
- Participants with persistent pain in both the upper and lower extremities, and participants with back pain were excluded from the analyses, decreasing the generalizability of these findings.
- The SF-8 minimal detectable difference and minimal clinical important difference literature is sparse, and does not evaluate athletic populations. This decreases the interpretability of these data.

For peer review only

INTRODUCTION

Upper and lower extremity osteoarthritis (OA) has a high incidence[1 2] and a substantial personal and societal burden.[3 4] Post-traumatic OA occurs at a younger age, with a longer period of disability and less effective treatments than idiopathic OA.[5-7] Former sport participants have increased post-traumatic OA prevalence compared to the general population,[5 8] which is associated with persistent joint pain.[9] Persistent joint pain is the major cause of patients seeking medical attention for OA,[9] and has been shown to influence a person's ability to participate in desired forms of activity[10 11] which may result in reduced physical activity levels and negatively impact health-related quality of life (HRQoL).[10-13]

To date, most research investigating OA and pain has focused on the lower extremity.[10-14] Former professional soccer players were found to have a high prevalence of lower extremity OA and pain compared to controls,[10 11] while former female recreational athletes were found to have 2 to 3 times increased hip and knee OA risk compared to population matched controls.[14] Lower extremity OA and pain in former sport participants is associated with impaired function and mobility, increased levels of anxiety and depression, and reduced HRQoL compared to the general population.[10 12-14]

Upper extremity pain can have different barriers to PA participation compared to lower extremity pain.[15-18] For example, lower extremity pain may have a greater effect on jumping, stair use, walking and running.[19 20] In contrast, upper extremity pain can have more effect on specific functional activities such as dressing and eating[15] or work related tasks such as carrying and handling objects[16] with potential negative impacts on HRQoL.[15] Upper extremity pain has been related to reduced PA and function, impaired HRQoL, stress and disability in workers.[21-25] Despite a high prevelance of upper extremity pain in former

For peer review only

For peer review only - http://bmjopen.bmj.com/site/about/guidelines.xhtml

throwing-sport participants,[26-28] the impact of upper extremity pain on PA levels and HRQoL in former athletes is poorly understood.

Psychological characteristics associated with successful high level athletic performance include resilience, mental toughness and advanced coping skills.[29-32] Greater resilience and coping strategies can have positive impact on PA levels[33-35] and HRQoL.[34 36-38] Thus, former elite athletes may possess psychological characteristics that enable them to better cope with chronic pain compared to former recreational sport participants, and this could positiviely impact PA levels and HRQoLHowever, this has not yet been investigated, highlighting the need for further research. It is also not clear if PA levels and HRQoL differ in former sport participants with and without perisistent joint pain. The rationale for investigating these relationships in former cricketers was largely due to the high prevalence of upper and lower extremity osteoarthritis and persistent pain amongst former cricketers,[12 39] and our previous qualitative work highlighting that a subgroup of former cricketers experience high quality of life and maintain physically active lifestyles despite persistent joint pain.[40 41] Therefore, the purpose of this study was to:

1) evaluate the relationship between persistent joint pain (upper extremity, lower extremity, or no persistent joint pain), PA levels and HRQoL in former cricketers;

2) compare these relationships in former elite and recreational cricketer subgroups.

METHODS

Study Design

This cross-sectional study was approved by the NHS Health Research Authority (NRES), London Stanmore Research Ethics Committee (REC 15/LO/1274).

For peer review only - http://bmjopen.bmj.com/site/about/guidelines.xhtml

Patient and public involvement

Findings from two qualitative studies investigating the relationship of physical activity and quality of life in former elite cricketers,[40 41] highlighted a need for further research investigating the relationship between cricket participation and health. In collaboration with stakeholders, including governing bodies and current and former cricketers, a questionnaire was developed and refined. This information was disseminated through presentations to stakeholders.

Participants and recruitment

In March 2017, 28,152 current and former cricketers of all playing standards, who were registered on a national database managed by the England and Wales Cricket Board, received an email inviting them to complete an electronic questionnaire. 2,598 people believed they met the eligibility requirements outlined in the email, and consented to participate in the Cricket Health and Wellbeing Study (Figure 1). Participants were eligible for inclusion in the Cricket Health and Wellbeing Study if they had played ≥1 cricket season and were aged ≥18 years. Due to the potential confounding relationship between acute injury and joint pain,[42] only former cricketers were included in this study. The other eligibility requirement for inclusion in this study, was reporting either i) persistent joint pain in only the upper extremity, ii) persistent joint pain in only the lower extremity, or iii) no persistent joint pain (individuals were excluded if they reported persistent back pain, or combined upper and lower extremity persistent joint pain). Participants were also excluded if they reported any memory impairment.

Questionnaire design

For peer review only

The Cricket Health and Wellbeing Study questionnaire was developed in collaboration with public involvement and four current/former cricketers piloted the questionnaire. The questionnaire was created to evaluate five aspects of health and wellbeing (i. cricket-related injury; ii. joint pain and osteoarthritis; iii. general health and disease prevalence; iv. physical activity; v. resilience, quality of life and flourishing). All participant data was managed and de-identified in a RedCap® (Research Electronic Data Capture) software database. The RedCap® database was managed by one investigator, and pilot tested by three researchers for errors and overall userbility.[43 44] The RedCap® software[44] utilized branching logic and allowed participants the option to save their current progress and complete at a later time. The questionnaire captured data including cricketers' age, body mass index (BMI), number of cricket seasons played, playing standard, presence of comorbidities, persistent pain, medically diagnosed OA, physical activity and HRQoL.

<Figure 1>

Outcomes

Physical activity

The International Physical Activity Questionnaire-Short Form (IPAQ-SF) was utilized to assess physical activity.[45] The IPAQ-SF has been used as a population surveillance and evaluation PA tool.[46 47] The IPAQ-SF has been observed to have fair agreement with accelerometer data (0.03-0.39)[48 49] and good validity compared to other PA questionnaires and PA logs (r>0.50).[49 50] Participants were asked to recall their PA in a usual week, as time spent in vigorous-intensity PA, moderate-intensity PA, and walking.[45] To reduce variability, a standardized approach outlined in the IPAQ-SF manual was utilized to clean and code the IPAQ-SF data.[45] In line with recommendations, bouts of weekly PA less than 10 minutes were recoded as zero minutes.[45] Time spent in vigorous PA, moderate PA, and walking was

For peer review only

For peer review only - http://bmjopen.bmj.com/site/about/guidelines.xhtml

converted to Metabolic Equivalents (METS). One MET is 1 kcal/kg/h, or the resting metabolic rate, during quiet sitting.[45] Vigorous PA was calculated as 8 METS per min, moderate PA was calculated as 4 METS per min, and walking was calculated as 3.3 METS per min.[45] Metabolic equivalents were truncated at three hours (180 minutes) per week, per vigorous, moderate, and walking PA.[45] This was performed in order to reduce participant overestimation effect.[45] A total of 117 (17%) of participants had PA reports truncated. METS were then combined for vigorous PA, moderate PA, and walking, and reported as total weekly METS.

Health related quality of life

The Short-Form 8 (SF-8) was used to assess HRQoL.[51] The SF-8 is a short version of the RAND 36-Item Health Survey (SF-36) 1.0,[52] the SF-8 is scaled and measured on the same point scale (0-100) as the SF-36, with 0 representing maximum disability and 100 representing no disability.[53] The SF-8 is an 8-item, self-reported HRQoL questionnaire comprising 8 domains (general health perceptions, physical function, bodily pain, physical role function, emotional role function, social function, vitality, and mental health)[53] and two component summary scores (the Physical Component Score (PCS) and Mental Component Score (MCS)). The PCS and MCS scores have been found to have high reliability (0.88 and 0.82 respectively) in the general United States population.[54] The PCS and MCS scores are calculated using a computerized algorithm, and weighted to the United States population mean.[54] A score of 50 is considered the population norm, and a score below 47 is considered below the average range of the general population.[54] The minimum detectable difference for the PCS in lower extremity OA patients is two points.[55] The minimally clinically important difference is estimated to be three to five points in the United States general population.[56]

For peer review only

For peer review only - http://bmjopen.bmj.com/site/about/guidelines.xhtml

Explanatory Variables

Persistent joint pain

Persistent joint pain was assessed with the following question, *'Do you currently experience pain, discomfort, or have any problems in any of your joints?'* If yes, participants were then asked *'Have you had pain in your [left/right] hip/groin, knee, ankle, shoulder, hand/finger, spine/back, other joint on most days of the last month?'*. All participants that recorded persistent pain in the "other joint" category were hand searched for references of elbow, wrist, foot, or toe persistent joint pain. These data were then recorded into separate categories of elbow/wrist, and foot/toes persistent joint pain. All persistent joint pain data was then separated into only upper extremity persistent joint pain (shoulder, elbow, wrist, hand and/or fingers), only lower extremity persistent joint pain (hip, knee, ankle, foot, and/or toes), and no persistent joint pain. If participants recorded spinal persistent pain, or upper and lower extremity persistent joint pain, subjects were excluded from the analyses.

Standard of play

Standard-of-play was assessed with the following question, '*what was the highest standard of cricket that you played for at least one season*?' Response options included: international; county/premier league; academy or county age group; university; school; village or social; don't know. Participants were stratified into recreational (university, school, village or social) and elite (international or county/premier league, academy or county age group). Participants that reported a playing standard as *'Don't know'* were excluded from all analyses.

Confounders

Potential confounders were identified through clinical reasoning and a review of the literature. Confounders included age, body mass index (BMI), and presence of a comorbidity.

For peer review only - http://bmjopen.bmj.com/site/about/guidelines.xhtml

For peer review only

Participants reported all medical problems that they had been diagnosed with. For the current study, diabetes, stroke and cancer were included as confounders due to a large potential to impact HRQoL and PA levels.[57-61] Presence of a comorbidity was defined as no comorbidity vs. one or more comorbidity.

Statistical Analyses

All data were assessed for missingness prior to analyses. Missing data were calculated as total and percentage of total data (Appendix 1). Due to the low percentage of missing data (MCS: 8.6%, PCS: 8.4%, IPAQ-SF: 5.9%, persistent joint pain: 1.1%), a complete case analysis was performed. 'Don't know' responses were excluded from all analyses. The Kruskall Wallis test was used to compare PA and HRQoL, between pain groups. To assess specific group to group differences, a series of Dunn's post hoc analyses were performed ($p<0.05$).

Continuous data were not assumed to act linearly on the outcomes. Thus, to evaluate the relationship between persistent joint pain, PA levels and HRQoL, multivariable linear regressions with fractional polynomials were used (to account for the non-linearity of the covariates).[62] This analysis was repeated in recreational and elite cricketer subgroups to address the second aim of this study. Unadjusted and adjusted coefficients and 95% confidence intervals (95% CI) were calculated. Coefficients were adjusted for the continuous variables age and BMI, and the presence of ≥1 comorbidity (yes vs. no). All assumptions for fractional polynomial regression were evaluated and satisfied.[62] Transformations included taking the square root of METS, PCS and MCS scores were not transformed due to regression residuals. Possible interactions were assessed for inclusion in the model. Interactions explored were between age and BMI, age and comorbidity, BMI and

For peer review only

comorbidity, age and persistent pain, BMI and persistent pain, and comorbidity and persistent pain. All analyses were performed in R version 3.5.1 (R Core Team (2013). R: A language and environment for statistical computing. R Foundation for Statistical Computing, Vienna, Austria. URL http://www.R-project.org/), using the dplyr package[63] for cleaning and coding, the naniar package for missingness assessment,[64] and the mfp package for fractional polynomial regression.[65]

RESULTS

A total of 703 former cricketers (aged mean 58.7 SD 12.9, played an average 30 IQR 20-40 seasons, 38% had played at an elite level) were included in analyses (Table 1). Body mass index in people with upper extremity persistent joint pain was 27.9 (SD 5.0), lower extremity persistent joint pain 29.1 (SD 5.5), and in people with no persistent joint pain was 27.8 (SD 5.4).

TABLE 1. Participant characteristics

	All former cricketers (n = 703)	Upper extremity persistent joint pain (n = 117)	Lower extremity persistent joint pain (n = 269)	No persistent joint pain (n = 317)
Age (years)	58.7 (SD 12.9)	59.9 (SD 12.6)	59.8 (SD 11.8)	57.8 (SD 13.8)
Sex				
Male	683 (97%)	113 (97%)	263 (98%)	307 (97%)
Female	15 (3%)	3 (3%)	4 (2%)	8 (3%)
NR	5 (<1%)	1 (<1%)	2 (<1%)	2 (<1%)
BMI (kg/m2)	28.3 (SD 5.3)	27.9 (SD 5.0)	29.1 (SD 5.5)	27.8 (SD 5.4)
Seasons Played	30 (IQR 20-40)	35 (IQR 27-44)	32 (IQR 23-42)	30 (IQR 18-48)
Years since last cricket match	7.4 (IQR 0.4-14.4)	8.1 (IQR 0.8-15.4)	6.9 (IQR 0.1-13.7)	6.7 (IQR 0.1-13.3)
Playing standard				
Elite	264 (38%)	46 (39%)	109 (41%)	109 (34%)
Recreational	428 (61%)	68 (58%)	156 (58%)	204 (65%)
NR	11 (1%)	3 (3%)	4 (1%)	4 (1%)
Comorbidity				
No	497 (73%)	81 (74%)	186 (71%)	230 (75%)
Yes	155 (23%)	23 (21%)	64 (25%)	68 (22%)
NR	27 (4%)	6 (5%)	10 (4%)	11 (4%)

For peer review only - http://bmjopen.bmj.com/site/about/guidelines.xhtml

SD = Standard Deviation, IQR = Inter Quartile Range, NR= No Response, BMI = Body Mass Index, [a]Participants reported the highest standard they had played for at least one season and then were stratified into recreational (university, school, village or social) and elite (international or county/premier league, academy or county age group)

A comparison of physical activity and HRQoL between former cricketers with upper extremity persistent joint pain, lower extremity persistent joint pain, and no persistent joint pain

For a visual representation of the PA data, please refer to Figure 2. Kruskall Wallis test indicated that there was no difference in physical activity levels between former cricketers with upper extremity persistent joint pain (Median (IQR): 2560 (722-4398) METS), lower extremity persistent joint pain (2215 (527-3903) METS), or no persistent joint pain (2449 (695-4203) METS) (Chi-square: 1.91, p=0.39). There were significant PCS differences between pain groups (p<0.001). Post hoc tests demonstrated that former cricketers reported worse PCS scores if they had persistent joint pain in the upper extremity (PCS: 49.8 (44.9-54.8)) or lower extremity (PCS: 46.7 (41.0-51.9)), compared to former cricketers with no persistant joint pain (PCS: 54.2 (51.5-56.9); Chi-square:121.2, p<0.001). Former cricketers with lower extremity persistent joint pain reported worse PCS scores compared to former cricketers with upper extremity persistent joint pain (Median Difference: 1.8, p=0.04). MCS scores were similar between individuals with upper extremity persistent joint pain (56.0 (52.1-60.0)), lower extremity persistent joint pain (55.2 (51.1-59.4)), and no persistent joint pain (54.7 (50.7-58.7)); Chi-squared: 1.92, p=0.38).

<Figure 2>

The relationship between persistent joint pain, physical activity and health related quality of life

For peer review only - http://bmjopen.bmj.com/site/about/guidelines.xhtml

Multivariable linear regressions found that persistent joint pain was not related to physical activity levels (Upper Extremity: Adjusted effect (95% CI): 28.1 METS (-1.1 to 135.7); Lower Extremity: 4.6 METS (-7.5 to 49.4)), see Table 2. Former cricketers with upper extremity persistent joint pain reported an estimated -5.5 (-7.4 to -3.5) points lower (worse) PCS scores than cricketers without persistent joint pain. Former cricketers with lower extremity persistent joint pain reported an estimated -6.6 (-8.1 to -5.2) points lower PCS scores compared to those with no persistent joint pain (Table 2). Persistent joint pain was not associated with MCS scores (Upper Extremity: -0.1 (-0.2 to 0.1); Lower Extremity: -0.1 (-0.2 to 0.1)).

For peer review only

For peer review only - http://bmjopen.bmj.com/site/about/guidelines.xhtml

TABLE 2. The relationship between persistent joint pain, physical activity and health related quality of life

	Weekly METS		PCS score		MCS score	
	Unadjusted Effect[b] (95% CI)	Adjusted[a] Effect[b] (95% CI)	Unadjusted Effect[c] (95% CI)	Adjusted[a] Effect[c] (95% CI)	Unadjusted Effect[c] (95% CI)	Adjusted[a] Effect[c] (95% CI)
Upper extremity persistent joint pain	20.3 (-3.2, 117), p=0.83	28.1 (-1.1, 136), p=0.10	-5.4 (-7.3, -3.6), p<0.001	-5.5 (-7.4, -3.5), p<0.001	-0.1 (-0.2, 0.1), p=0.35	-0.1 (-0.2, 0.1), p=0.26
Lower extremity persistent joint pain	0.3 (-12.5, 21.0), p=0.16	4.6 (-7.5, 49.4), p=0.39	-6.9 (-8.4, -5.5), p<0.001	-6.6 (-8.1, -5.2), p<0.001	0.09 (-0.2, 0.1), p=0.08	-0.1 (-0.2, 0.1), p=0.06
No persistent joint pain	*Reference group*		*Reference group*		*Reference group*	

[a] Estimates are adjusted for age, body mass index, and comorbidities
[b] Participants with memory impairments were excluded from the analyses
[c] Comorbidities were defined as none present (0) and presence of at least one comorbidity (1). Comorbidities included were diabetes, stroke, skin cancer, and other cancer
[d] Upper extremity (shoulder, elbow, wrist, hand) and lower extremity (hip, knee, ankle) persistent joint pain were assessed by asking individuals if they had joint-specific pain on 'most days of the last month.'
[e] Questionnaire Short-Form (IPAQ-SF); Physical activity was calculated as metabolic equivalents (METS) per week; METS were transformed prior to analysis by taking the square root and then retransformed by squaring after analysis
[f] SF-8: Short-Form 8 Health Survey; Physical Component Scores (PCS) and Mental Component Scores (MCS) were calculated using norm based scoring (population norm 50 SD 10, high scorer = better health-related quality of life)

Elite vs. recreational cricketer subgroup analysis

Multivariable linear regressions found that findings were similar between recreational and elite cricketer subgroups (Table 3). Persistent joint pain was not associated with physical activity and MCS scores, and PCS scores were impaired in both former elite and recreational cricketers with upper extremity (Adjusted effect (95% CI) Elite: -5.3 (-8.8, -1.9); Recreational: -5.8 (-8.1, -3.4)) or lower extremity (Elite: -7.0 (-9.7, -4.3); Recreational: -6.2 (-8.0, -4.5)) persistent joint pain, compared to their counterparts with no persistent joint pain (Table 3).

For peer review only - http://bmjopen.bmj.com/site/about/guidelines.xhtml

TABLE 3. The relationship between persistent joint pain, physical activity and health related quality of life, in former recreational and elite cricketer subgroups

		Weekly METS		PCS score		MCS score	
Former elite cricketers (n=280)		Unadjusted Effect[b] (95% CI)	Adjusted[a] Effect[b] (95% CI)	Unadjusted Effect[c] (95% CI)	Adjusted[a] Effect[c] (95% CI)	Unadjusted Effect[c] (95% CI)	Adjusted[a] Effect[c] (95% CI)
	Upper extremity persistent joint pain	70 (-1.6, 324), p=0.09	88 (-0.1, 356), p=0.05	-5.3 (-8.6, -2.0), p=0.001	-5.3 (-8.8, -1.9), p=0.002	0.055 (-2.3, 3.4), p=0.70	5.3 (-1.1, 11.7), p=0.10
	Lower extremity persistent joint pain	5.2 (-28, 97), p=0.56	8.2 (-21, 106), p=0.45	-7.5 (-10.1, -4.9), p<0.001	-7.0 (-9.7, -4.3), p<0.001	0.1 (-1.7, 2.7), p=0.64	2.2 (-2.7, 7.0), p=0.39
	No persistent joint pain	*Reference group*		*Reference group*		*Reference group*	
Former recreational cricketers (n=423)		Unadjusted Effect[b] (95% CI)	Adjusted[a] Effect[b] (95% CI)	Unadjusted Effect[c] (95% CI)	Adjusted[a] Effect[c] (95% CI)	Unadjusted Effect[c] (95% CI)	Adjusted[a] Effect[c] (95% CI)
	Upper extremity persistent joint pain	5.6 (-37, 117), p=0.58	10.5 (-29, 149), p=0.47	-5.6 (-7.9, -3.3), p<0.001	-5.8 (-8.1, -3.4), p<0.001	0.8 (-1.5, 3.0), p=0.50	1.1 (-1.1, 3.2), p=0.35
	Lower extremity persistent joint pain	-0.7 (-49, 29), p=0.83	1.8 (-26, 61), p=0.68	-6.6 (-8.3, -4.9), p<0.001	-6.2 (-8.0, -4.5), p<0.001	-0.8 (-1.1, 2.7), p=0.33	1.2 (-0.5, 2.8), p=0.17

For peer review only

For peer review only - http://bmjopen.bmj.com/site/about/guidelines.xhtml

No persistent joint pain	*Reference group*	*Reference group*	*Reference group*

[a] Estimates are adjusted for age, body mass index, and comorbidities
[b] Participants with memory impairments were excluded from the analyses
[c] Comorbidities were defined as not present (0) and presence of at least one comorbidity (1). Comorbidities included were diabetes, stroke, skin cancer, and other cancer
[e] Upper extremity (shoulder, elbow, wrist, or hand) and lower extremity (hip, knee, or ankle) persistent joint pain were assessed by asking individuals if they had joint-specific pain on 'most days of the last month.'
[f] Questionnaire Short-Form (IPAQ-SF); Physical activity was calculated as metabolic equivalents (METS) per week; METS were transformed prior to analysis by taking the square root and then retransformed by squaring after analysis
[g] SF-8: Short-Form 8 Health Survey; Physical Component Scores (PCS) were calculated using norm based scoring (population norm 50 SD 10, high scorer = better health-related quality of life); Mental Component Scores (MCS) were calculated using norm based scoring (population norm 50 SD 10, high scorer = better health-related quality of life)

For peer review only

DISCUSSION

Suprisingly, despite impaired PCS scores, there was no difference in weekly PA or MCS scores, between former cricketers with upper or lower extremity persistent joint pain, and those with no persistent joint pain. Additionally, former cricketers with lower extremity persistent joint pain reported worse physical-components of HRQoL than those with upper extremity persistent joint pain. We also found that the relationship between joint pain, PA and HRQoL were similar amougnst former elite and recreational cricketer subgroups.

Former cricketers with persistent joint pain did not report reduced weekly PA compared to former cricketers without persistent joint pain. In the general population, people with OA have reduced PA levels compared to matched controls without OA.[66] In this study, former cricketers with and without persistent joint pain had higher PA levels compared to the general population.[67-69] Former cricketers reported median daily PA levels of 120 METS per day, this is in comparison to an average range of 41 to 50 METS per day for European men.[67-69] Former cricketers may find alternative strategies to exercise, despite persistent joint pain.[41 70] In a qualitative study, former cricketers with pain and physical impairment found alternative strategies to perform PA, and shared specific psychological strengths that may enable them to effectively cope with joint pain.[41] Intrinsic motivation,[71] higher levels of resilience,[32 40] and effective coping strategies[40 41] are all characteristics common amongst successful athletes. Such psychological strengths may be more common in former sport participants compared to the general population.[29-32] Additionally, sport participants may be encouraged to play through pain[72] and are often rewarded for doing so.[73] Such pain behaviours could influence how former sport participants perceive and cope with joint pain. Pain behaviours and psychological strengths common amougnst sport participants could provide a potential explanation for the high physical activity levels in former cricketers living with joint pain.

For peer review only

Further research is required to understand the potential interplay between persistent pain, psychological characteristics, and PA in former athletes.

The psychological strengths that are common amoungst former sports participants including resilience,[32 40] mental toughness,[74 75] and pain coping strategies,[40 75] could also provide a potential explanation for why MCS scores were not impaired in former cricketers with persistent joint pain, despite impaired PCS scores. In a recent meta-analysis, former athletes with impaired PCS scores reported greater MCS scores compared to general population norms.[76] In a typical OA population, both PCS and MCS scores are impaired,[77] suggesting HRQoL in former sport participants with OA may differ from the typical OA population. Furthermore, the high PA levels in former cricketers in our study could also contribute to the high MCS scores. Physical activity has been shown to have a protective effect against depression and a positive impact on mental-components of HRQoL in the general population, older adults, and in former elite athletes.[78-81] Irrespective of joint pain, former cricketers reported MCS scores 5 to 6 points better than the population normative average, and this difference is likely to be clinically meaningful.[82]

Former cricketers with persistent joint pain had impaired physical-components of HRQoL compared to cricketers without persistent pain. This supports previous literature which found impaired PCS scores in former collegiate[83-85] and professional athletes[86] compared to non-athlete controls. Former cricketers with persistent pain reported PCS scores 5 to 7 points lower than former cricketers without persistent pain, demonstrating a clinical important difference between persistent pain and no pain groups. Impaired physical-components of HRQoL in former athletes has been attributed to poor joint health,[83-85] which may be due to previous musculoskeletal injury.[87-89] Former athletes with a history of musculoskeletal injury

For peer review only

For peer review only - http://bmjopen.bmj.com/site/about/guidelines.xhtml

report worse physical-components of HRQoL, than former athletes without a history of injury[87] and the general population.[88 89] In our study, former cricketers without persistent pain reported a median PCS score 4 points above the population normative mean of 50, suggesting that former cricket participants without joint pain have greater levels of physical functioning compared to the general population. Importantly, despite impaired PCS scores in former cricketers with persistent pain, MCS scores were not impaired. This highlights the importance of selecting a HRQoL measure that differentiates assessment of pain and physical function, from assessment of mental health and wellbeing, when evaluating HRQoL in former sport participants.

Cricketers with persistent lower extremity pain reported 3.1 points worse PCS scores compared to cricketers with upper extremity pain, and this difference is likely to be clinically meaningful.[82] Worse PCS scores in former cricketers with lower extremity pain may be due to greater difficulties performing usual physical activities (such as difficulties with ambulation and stair navigation[90 91]) or experiencing more severe bodily pain than those who report upper extremity pain. For example, former collegiate athletes with lower extremity persistent pain were 2.5 times more likely to be limited in physical function, such as stair navigation, and have greater impaired HRQoL, compared to non-athletes.[84] While there is research investigating the impacts of lower extremity pain on function in former athletes, our understanding of the impact of upper extremity pain on function in former athletes is poor. Further research could inform strategies to improve physical-components of HRQoL amongst former athletes with persistent upper extremity pain.

The relationship between persistent joint pain, PA, and HRQoL was similar between elite and recreational former cricketers. Few studies have investigated PA or HRQoL in former

For peer review only - http://bmjopen.bmj.com/site/about/guidelines.xhtml

athletes from different standards-of-play. Higher standards of sport competition requires elevated levels of resilience and psychological hardiness due to the increased levels of training and competition.[29-32 92] Despite the observed psychological strengths associated with elite cricket participation, any level of sport participation is associated with favourable psychological strengths such as resilience.[30 32 92] Thus, it is possible that all standards of cricket participation is associated with psychological benefits that may explain the high PA levels and mental-components of HRQoL in former cricketers living with persistant pain; further research is needed to explore this possibility.

Strengths and potential limitations

This study took into account the non-linear relationships between different continuous variables (i.e. by using fractional polynomials), where most studies assume linearity.[93] Missing data was low, decreasing the risk of bias in a complete case analysis. Physical activity was truncated above 180 minutes, which may introduce a ceiling effect. Further, PA recall may not be as prcise as PA monitors, which may introduce measurement error, and social desirability may cause PA overestimation.[94 95] Persistent joint pain was assessed by asking cricketers if they had joint pain on most days of the last month, which is based on the National health and Nutrition Examination Survey (NHANES). However, joint pain may occur intermittently, and still effect PA and HRQoL. As a result, this study may have missed participants with significant pain. Participants with persistent pain in both the upper and lower extremities, and participants with back pain were excluded from the analyses. This limits the scope of this study to participants with only upper or lower extremity persistent pain, or no persistent pain, decreasing the generalisability of these findings. The SF-8 minimal detectable difference and minimal clinical important difference literature is sparse, and does not evaluate athletic populations. This decreases the interpretability of these data.

For peer review only - http://bmjopen.bmj.com/site/about/guidelines.xhtml

There is also the possibility of bias due to unmeasured confounding. Potential confounders that were not measured in this study include socio-economic status and other sports played, which both have been observed to be related to HRQoL.[76 96 97] These discrepancies may decrease the generalisability of these findings. Potential participants were informed of the inclusion and exclusion criteria during recruitment, and were able to self-select eligibility to participate. Due to this recruitment strategy, it is not possible to determine the questionnaire response rate, nor responder bias. This recruitment strategy may decrease the generalisabilty of these findings.

Conclusions

Physical components of HRQoL were impaired in former cricketers with persistent joint pain compared to those without joint pain, and PCS scores were more impaired in those with lower extremity pain compared to those with upper extremity pain. However, former cricketers with persistent joint pain did not have reduced PA levels or mental components of HRQoL, compared to those with no persistent joint pain. These relationships were similar amongst former elite and recreational cricketers. These results highlight the need to select a HRQoL measure that differentiates assessment of pain and physical functional, from assessment of mental health and wellbeing, when evaluating HRQoL in former sport participants. The high average physical activity levels amongst former cricketers with persistent joint pain could explain the high MCS scores in this group. In contrast, former cricketers with joint pain who are physically inactive may benefit from targeted strategies to increase activity levels, with potential to positively impact QOL. Further research is needed to determine whether the psychological benefits of sport participation explain why former cricketers had high PA levels and mental-components of HRQoL, despite living with persistant joint-pain.

For peer review only - http://bmjopen.bmj.com/site/about/guidelines.xhtml

Author's Contributions

GSB, GSC, NP, NKA, SRF conceived the study idea. GSB, GSC, NP, NKA, SRF were involved in methodological design and planning. GSB and SRF wrote the first draft of the manuscript. GSB, GSC, SRF contributed to statistical analyses. GSB, GSC, NP, NKA, SRF all critically revised the manuscript. GSB, GSC, NP, NKA, SRF all approved the final version of the manuscript.

Funding: This study was funded by Centre for Sport, Exercise and Osteoarthritis Research Versus Arthritis (grant reference 21595). Gary Collins was supported by the NIHR Biomedical Research Centre, Oxford.

Data Availability

Data is available upon request. Please contact the authors for further inquiry.

Figure and Table Captions

Figure 1. Study Flow Chart

Table 1. Participant Characteristics

Figure 2. Weekly physical activity levels and health related quality of life in former cricketers with upper extremity persistent joint pain, lower extremity persistent joint pain, or no persistent joint pain

Table 2. The relationship between persistent joint pain, physical activity and health related quality of life

Table 3. The relationship between persistent joint pain, physical activity and health related quality of life, in former recreational and elite cricketer subgroups

For peer review only

For peer review only

References

1. Plotnikoff R, Karunamuni N, Lytvyak E, et al. Osteoarthritis prevalence and modifiable factors: a population study. *BMC public health* 2015;15:1195. doi: 10.1186/s12889-015-2529-0 [published Online First: 2015/12/02]
2. van Schoor NM, Zambon S, Castell MV, et al. Impact of clinical osteoarthritis of the hip, knee and hand on self-rated health in six European countries: the European Project on OSteoArthritis. *Quality of life research : an international journal of quality of life aspects of treatment, care and rehabilitation* 2016;25(6):1423-32. doi: 10.1007/s11136-015-1171-8 [published Online First: 2015/11/09]
3. Palazzo C, Nguyen C, Lefevre-Colau MM, et al. Risk factors and burden of osteoarthritis. *Annals of physical and rehabilitation medicine* 2016;59(3):134-38. doi: 10.1016/j.rehab.2016.01.006 [published Online First: 2016/02/26]
4. Cross M, Smith E, Hoy D, et al. The global burden of hip and knee osteoarthritis: estimates from the global burden of disease 2010 study. *Annals of the rheumatic diseases* 2014;73(7):1323-30. doi: 10.1136/annrheumdis-2013-204763 [published Online First: 2014/02/21]
5. Carbone A, Rodeo S. Review of current understanding of post-traumatic osteoarthritis resulting from sports injuries. *Journal of orthopaedic research : official publication of the Orthopaedic Research Society* 2017;35(3):397-405. doi: 10.1002/jor.23341 [published Online First: 2016/06/17]
6. Brown TD, Johnston RC, Saltzman CL, et al. Posttraumatic osteoarthritis: a first estimate of incidence, prevalence, and burden of disease. *Journal of orthopaedic trauma* 2006;20(10):739-44. doi: 10.1097/01.bot.0000246468.80635.ef [published Online First: 2006/11/16]
7. Vannini F, Spalding T, Andriolo L, et al. Sport and early osteoarthritis: the role of sport in aetiology, progression and treatment of knee osteoarthritis. *Knee surgery, sports traumatology, arthroscopy : official journal of the ESSKA* 2016;24(6):1786-96. doi: 10.1007/s00167-016-4090-5 [published Online First: 2016/04/05]
8. Whittaker JL, Woodhouse LJ, Nettel-Aguirre A, et al. Outcomes associated with early post-traumatic osteoarthritis and other negative health consequences 3-10 years following knee joint injury in youth sport. *Osteoarthritis and cartilage* 2015;23(7):1122-9. doi: 10.1016/j.joca.2015.02.021 [published Online First: 2015/03/01]
9. Neogi T. The epidemiology and impact of pain in osteoarthritis. *Osteoarthritis and cartilage* 2013;21(9):1145-53. doi: 10.1016/j.joca.2013.03.018 [published Online First: 2013/08/27]
10. Arliani GG, Astur DC, Yamada RK, et al. Early osteoarthritis and reduced quality of life after retirement in former professional soccer players. *Clinics (Sao Paulo, Brazil)* 2014;69(9):589-94. [published Online First: 2014/10/16]
11. Turner AP, Barlow JH, Heathcote-Elliott C. Long term health impact of playing professional football in the United Kingdom. *British journal of sports medicine* 2000;34(5):332-6. [published Online First: 2000/10/26]
12. Jones ME, Davies MAM, Leyland KM, et al. Osteoarthritis and other long-term health conditions in former elite cricketers. *Journal of science and medicine in sport* 2018;21(6):558-63. doi: 10.1016/j.jsams.2017.10.013 [published Online First: 2017/11/02]
13. Davies MAM, A DJ, Delmestri A, et al. Health amongst former rugby union players: A cross-sectional study of morbidity and health-related quality of life. *Scientific reports* 2017;7(1):11786. doi: 10.1038/s41598-017-12130-y [published Online First: 2017/09/30]
14. Spector TD, Harris PA, Hart DJ, et al. Risk of osteoarthritis associated with long-term weight-bearing sports: a radiologic survey of the hips and knees in female ex-athletes and population controls. *Arthritis and rheumatism* 1996;39(6):988-95. [published Online First: 1996/06/01]

For peer review only

15. Lang CE, Bland MD, Bailey RR, et al. Assessment of upper extremity impairment, function, and activity after stroke: foundations for clinical decision making. *Journal of hand therapy : official journal of the American Society of Hand Therapists* 2013;26(2):104-14;quiz 15. doi: 10.1016/j.jht.2012.06.005 [published Online First: 2012/09/15]

16. Shaw WS, Feuerstein M, Lincoln AE, et al. Ergonomic and psychosocial factors affect daily function in workers' compensation claimants with persistent upper extremity disorders. *Journal of occupational and environmental medicine* 2002;44(7):606-15. [published Online First: 2002/07/24]

17. Peters SE, Johnston V, Ross M, et al. Expert consensus on facilitators and barriers to return-to-work following surgery for non-traumatic upper extremity conditions: a Delphi study. *The Journal of hand surgery, European volume* 2017;42(2):127-36. doi: 10.1177/1753193416669263 [published Online First: 2016/09/23]

18. Hundza S, Quartly C, Kim JM, et al. Similar barriers and facilitators to physical activity across different clinical groups experiencing lower limb spasticity. *Disability and rehabilitation* 2016;38(14):1370-81. doi: 10.3109/09638288.2015.1101789 [published Online First: 2016/01/05]

19. Barnes KR, McGuigan MR, Kilding AE. Lower-body determinants of running economy in male and female distance runners. *Journal of strength and conditioning research* 2014;28(5):1289-97. doi: 10.1519/jsc.0000000000000267 [published Online First: 2013/10/16]

20. Vogt MT, Lauerman WC, Chirumbole M, et al. A community-based study of postmenopausal white women with back and leg pain: health status and limitations in physical activity. *The journals of gerontology Series A, Biological sciences and medical sciences* 2002;57(8):M544-50. [published Online First: 2002/07/30]

21. Montero A, Mulero JF, Tornero C, et al. Pain, disability and health-related quality of life in osteoarthritis-joint matters: an observational, multi-specialty trans-national follow-up study. *Clin Rheumatol* 2016;35(9):2293-305. doi: 10.1007/s10067-016-3248-3 [published Online First: 2016/04/14]

22. Verma C, Parikh R, Nadkar M, et al. Correlation of Functional Ability of the Hand with Upper Limb Function and Quality of Life in Patients with Rheumatoid Arthritis. *J Assoc Physicians India* 2017;65(3):20-24. [published Online First: 2017/05/04]

23. Rincon-Hurtado AM, Rocha-Buelvas A, Lopez-Cardona A, et al. Health-related quality of life of patients with rotator cuff injuries, Cofee Triangle, Colombia, 2013. *Rev Bras Ortop* 2018;53(3):364-72. doi: 10.1016/j.rboe.2018.03.018 [published Online First: 2018/06/13]

24. Cassou B, Derriennic F, Monfort C, et al. Chronic neck and shoulder pain, age, and working conditions: longitudinal results from a large random sample in France. *Occup Environ Med* 2002;59(8):537-44. [published Online First: 2002/08/02]

25. Grzywacz JG, Arcury TA, Mora D, et al. Work organization and musculoskeletal health: clinical findings from immigrant Latino poultry processing and other manual workers. *J Occup Environ Med* 2012;54(8):995-1001. doi: 10.1097/JOM.0b013e318254640d [published Online First: 2012/07/24]

26. Sauers EL, Dykstra DL, Bay RC, et al. Upper extremity injury history, current pain rating, and health-related quality of life in female softball pitchers. *Journal of sport rehabilitation* 2011;20(1):100-14. [published Online First: 2011/03/18]

27. Soomro N, Strasiotto L, Sawdagar T, et al. Cricket Injury Epidemiology in the Twenty-First Century: What is the Burden? *Sports medicine (Auckland, NZ)* 2018;48(10):2301-16. doi: 10.1007/s40279-018-0960-y [published Online First: 2018/07/19]

28. Posner M, Cameron KL, Wolf JM, et al. Epidemiology of Major League Baseball injuries. *The American journal of sports medicine* 2011;39(8):1676-80. doi: 10.1177/0363546511411700 [published Online First: 2011/06/29]

29. Cowden RG, Fuller DK, Anshel MH. Psychological predictors of mental toughness in elite tennis: an exploratory study in learned resourcefulness and competitive trait anxiety. *Perceptual*

For peer review only

and motor skills 2014;119(3):661-78. doi: 10.2466/30.PMS.119c27z0 [published Online First: 2014/11/12]
30. Secades XG, Molinero O, Salguero A, et al. Relationship Between Resilience and Coping Strategies in Competitive Sport. *Perceptual and motor skills* 2016;122(1):336-49. doi: 10.1177/0031512516631056 [published Online First: 2016/07/16]
31. Shields MR, Brooks MA, Koltyn KF, et al. Cognitive Resilience and Psychological Responses across a Collegiate Rowing Season. *Medicine and science in sports and exercise* 2017;49(11):2276-85. doi: 10.1249/mss.0000000000001363 [published Online First: 2017/07/07]
32. Sarkar M, Fletcher D. Psychological resilience in sport performers: a review of stressors and protective factors. *Journal of sports sciences* 2014;32(15):1419-34. doi: 10.1080/02640414.2014.901551 [published Online First: 2014/04/11]
33. Wermelinger Avila MP, Correa JC, Lucchetti ALG, et al. The Role of Physical Activity in the Association Between Resilience and Mental Health in Older Adults. *Journal of aging and physical activity* 2018;26(2):248-53. doi: 10.1123/japa.2016-0332 [published Online First: 2017/07/18]
34. Taylor K, Scruggs PW, Balemba OB, et al. Associations between physical activity, resilience, and quality of life in people with inflammatory bowel disease. *European journal of applied physiology* 2018;118(4):829-36. doi: 10.1007/s00421-018-3817-z [published Online First: 2018/02/08]
35. Seaton CL, Bottorff JL, Jones-Bricker M, et al. The Role of Positive Emotion and Ego-Resilience in Determining Men's Physical Activity Following a Workplace Health Intervention. *American journal of men's health* 2018;12(6):1916-28. doi: 10.1177/1557988318803744 [published Online First: 2018/10/20]
36. Paffenbarger RS, Jr., Lee IM, Leung R. Physical activity and personal characteristics associated with depression and suicide in American college men. *Acta psychiatrica Scandinavica Supplementum* 1994;377:16-22. [published Online First: 1994/01/01]
37. Camacho TC, Roberts RE, Lazarus NB, et al. Physical activity and depression: evidence from the Alameda County Study. *American journal of epidemiology* 1991;134(2):220-31. [published Online First: 1991/07/15]
38. Farmer ME, Locke BZ, Moscicki EK, et al. Physical activity and depressive symptoms: the NHANES I Epidemiologic Follow-up Study. *American journal of epidemiology* 1988;128(6):1340-51. [published Online First: 1988/12/01]
39. Jones ME, Davies MA, Leyland KM, et al. The association of previous injury with joint pain, osteoarthritis, and joint replacement across all joints in former elite English cricketers. *Osteoarthritis and Cartilage* 2017;25 (Supplement 1):S200-S01.
40. Filbay SR, Bishop F, Peirce N, et al. Common attributes in retired professional cricketers that may enhance or hinder quality of life after retirement: a qualitative study. *BMJ open* 2017;7(7):e016541. doi: 10.1136/bmjopen-2017-016541 [published Online First: 2017/07/29]
41. Filbay SR, Bishop FL, Peirce N, et al. Physical activity in former elite cricketers and strategies for promoting physical activity after retirement from cricket: a qualitative study. *BMJ open* 2017;7(11):e017785. doi: 10.1136/bmjopen-2017-017785 [published Online First: 2017/11/21]
42. Igolnikov I, Gallagher RM, Hainline B. Sport-related injury and pain classification. *Handbook of clinical neurology* 2018;158:423-30. doi: 10.1016/b978-0-444-63954-7.00039-2 [published Online First: 2018/11/30]
43. Harris PA, Taylor R, Thielke R, et al. Research electronic data capture (REDCap)--a metadata-driven methodology and workflow process for providing translational research informatics support. *Journal of biomedical informatics* 2009;42(2):377-81. doi: 10.1016/j.jbi.2008.08.010 [published Online First: 2008/10/22]

For peer review only

44. Lyon JA, Garcia-Milian R, Norton HF, et al. The use of Research Electronic Data Capture (REDCap) software to create a database of librarian-mediated literature searches. *Medical reference services quarterly* 2014;33(3):241-52. doi: 10.1080/02763869.2014.925379 [published Online First: 2014/07/16]

45. Committee IR. Guidelines for data processing and analysis of the International Physical Activity Questionnaire (IPAQ)-short and long forms. *http://www ipaq ki se/scoring pdf* 2005

46. Rosemann T, Kuehlein T, Laux G, et al. Osteoarthritis of the knee and hip: a comparison of factors associated with physical activity. *Clinical rheumatology* 2007;26(11):1811-7. doi: 10.1007/s10067-007-0579-0 [published Online First: 2007/03/03]

47. Hultenheim Klintberg I, Karlsson J, Svantesson U. Health-related quality of life, patient satisfaction, and physical activity 8-11 years after arthroscopic subacromial decompression. *Journal of shoulder and elbow surgery* 2011;20(4):598-608. doi: 10.1016/j.jse.2010.08.021 [published Online First: 2010/12/15]

48. Ekelund U, Sepp H, Brage S, et al. Criterion-related validity of the last 7-day, short form of the International Physical Activity Questionnaire in Swedish adults. *Public health nutrition* 2006;9(2):258-65.

49. Mader U, Martin BW, Schutz Y, et al. Validity of four short physical activity questionnaires in middle-aged persons. *Medicine and science in sports and exercise* 2006;38(7):1255-66. doi: 10.1249/01.mss.0000227310.18902.28 [published Online First: 2006/07/11]

50. Macfarlane DJ, Lee CC, Ho EY, et al. Convergent validity of six methods to assess physical activity in daily life. *Journal of applied physiology (Bethesda, Md : 1985)* 2006;101(5):1328-34. doi: 10.1152/japplphysiol.00336.2006 [published Online First: 2006/07/11]

51. Turner-Bowker DM, Bayliss MS, Ware JE, Jr., et al. Usefulness of the SF-8 Health Survey for comparing the impact of migraine and other conditions. *Quality of life research : an international journal of quality of life aspects of treatment, care and rehabilitation* 2003;12(8):1003-12. [published Online First: 2003/12/04]

52. Hays RD, Sherbourne CD, Mazel RM. The RAND 36-Item Health Survey 1.0. *Health economics* 1993;2(3):217-27. [published Online First: 1993/10/01]

53. Ware JE, Jr., Sherbourne CD. The MOS 36-item short-form health survey (SF-36). I. Conceptual framework and item selection. *Medical care* 1992;30(6):473-83. [published Online First: 1992/06/11]

54. Ware JE, Kosinski M, Dewey JE, et al. How to score and interpret single-item health status measures: a manual for users of the SF-8 health survey. *Lincoln, RI: QualityMetric Incorporated* 2001;15(10):5.

55. Angst F, Aeschlimann A, Stucki G. Smallest detectable and minimal clinically important differences of rehabilitation intervention with their implications for required sample sizes using WOMAC and SF-36 quality of life measurement instruments in patients with osteoarthritis of the lower extremities. *Arthritis Care & Research: Official Journal of the American College of Rheumatology* 2001;45(4):384-91.

56. Samsa G, Edelman D, Rothman ML, et al. Determining clinically important differences in health status measures. *Pharmacoeconomics* 1999;15(2):141-55.

57. Mols F, Vingerhoets AJ, Coebergh JW, et al. Quality of life among long-term breast cancer survivors: a systematic review. *European journal of cancer* 2005;41(17):2613-19.

58. Doyle C, Kushi LH, Byers T, et al. Nutrition and physical activity during and after cancer treatment: an American Cancer Society guide for informed choices. *CA: a cancer journal for clinicians* 2006;56(6):323-53.

59. Manson JE, Stampfer M, Colditz G, et al. Physical activity and incidence of non-insulin-dependent diabetes mellitus in women. *The Lancet* 1991;338(8770):774-78.

60. Goldney RD, Phillips PJ, Fisher LJ, et al. Diabetes, depression, and quality of life: a population study. *Diabetes care* 2004;27(5):1066-70.

For peer review only - http://bmjopen.bmj.com/site/about/guidelines.xhtml

61. Moore SA, Hallsworth K, Plötz T, et al. Physical activity, sedentary behaviour and metabolic control following stroke: a cross-sectional and longitudinal study. *PloS one* 2013;8(1):e55263.
62. Royston P, Altman DG. Regression using fractional polynomials of continuous covariates: parsimonious parametric modelling. *Journal of the Royal Statistical Society: Series C (Applied Statistics)* 1994;43(3):429-53.
63. Hadley Wickham RF, Lionel Henry, Kirill Muller. dplyr: A Grammar of Data Manipulation 2018 [R package version

0.7.6.:[Available from: https://CRAN.R-project.org/package=dplyr.
64. Nicholas Tierney DC, Miles McBain, Colin Fay, Mitchell O'Hara-Wild, Jim Hester, Luke Smith. naniar 2018 [R Package version: 0.4.1:[Available from: https://cran.r-project.org/web/packages/naniar/index.html.
65. Gareth Ambler AB. mfp. R package 1.5.2 ed2015.
66. Wallis JA, Webster KE, Levinger P, et al. What proportion of people with hip and knee osteoarthritis meet physical activity guidelines? A systematic review and meta-analysis. *Osteoarthritis and cartilage* 2013;21(11):1648-59. doi: 10.1016/j.joca.2013.08.003 [published Online First: 2013/08/21]
67. Aadahl M, Kjaer M, Jorgensen T. Associations between overall physical activity level and cardiovascular risk factors in an adult population. *European journal of epidemiology* 2007;22(6):369-78. doi: 10.1007/s10654-006-9100-3 [published Online First: 2007/03/03]
68. Schaller N, Seiler H, Himmerich S, et al. Estimated physical activity in Bavaria, Germany, and its implications for obesity risk: results from the BVS-II Study. *International Journal of Behavioral Nutrition and Physical Activity* 2005;2(1):6.
69. Norman A, Bellocco R, Bergström A, et al. Validity and reproducibility of self-reported total physical activity—differences by relative weight. *International journal of obesity* 2001;25(5):682.
70. Holden MA, Nicholls EE, Young J, et al. Exercise and physical activity in older adults with knee pain: a mixed methods study. *Rheumatology (Oxford, England)* 2015;54(3):413-23. doi: 10.1093/rheumatology/keu333 [published Online First: 2014/09/05]
71. Teixeira PJ, Carraça EV, Markland D, et al. Exercise, physical activity, and self-determination theory: a systematic review. *International journal of behavioral nutrition and physical activity* 2012;9(1):78.
72. Hammond LE, Lilley JM, Pope GD, et al. 'We've just learnt to put up with it': an exploration of attitudes and decision-making surrounding playing with injury in English professional football. *Qualitative Research in Sport, Exercise and Health* 2014;6(2):161-81.
73. Roderick M, Waddington I, Parker G. Playing hurt: Managing injuries in English professional football. *International review for the sociology of sport* 2000;35(2):165-80.
74. Meggs J, Ditzfeld C, Golby J. Self-concept organisation and mental toughness in sport. *Journal of sports sciences* 2014;32(2):101-9. doi: 10.1080/02640414.2013.812230 [published Online First: 2013/08/24]
75. Nicholls AR, Polman RCJ, Levy AR, et al. Mental toughness, optimism, pessimism, and coping among athletes. *Personality and Individual Differences* 2008;44(5):1182-92. doi: https://doi.org/10.1016/j.paid.2007.11.011
76. FIlbay SR PT, Thomas B, McKay C, Adams J, Arden NK. Quality of life and life satisfaction in former athletes: a systematic review and meta-analysis

. *Sports Medicine* 2019
77. Kobayashi S, Pappas E, Fransen M, et al. The prevalence of patellofemoral osteoarthritis: a systematic review and meta-analysis. *Osteoarthritis and cartilage* 2016;24(10):1697-707. doi: 10.1016/j.joca.2016.05.011 [published Online First: 2016/05/18]

For peer review only - http://bmjopen.bmj.com/site/about/guidelines.xhtml

78. Harvey SB, Overland S, Hatch SL, et al. Exercise and the Prevention of Depression: Results of the HUNT Cohort Study. *The American journal of psychiatry* 2018;175(1):28-36. doi: 10.1176/appi.ajp.2017.16111223 [published Online First: 2017/10/04]

79. Salmon P. Effects of physical exercise on anxiety, depression, and sensitivity to stress: a unifying theory. *Clinical psychology review* 2001;21(1):33-61. [published Online First: 2001/01/10]

80. Vasiliadis HM, Belanger MF. The prospective and concurrent effect of exercise on health related quality of life in older adults over a 3 year period. *Health and quality of life outcomes* 2018;16(1):15. doi: 10.1186/s12955-018-0843-9 [published Online First: 2018/01/18]

81. Bäckmand H, Kaprio J, Kujala U, et al. Influence of physical activity on depression and anxiety of former elite athletes. *International journal of sports medicine* 2003;24(08):609-19.

82. Hays RD, Morales LS. The RAND-36 measure of health-related quality of life. *Annals of medicine* 2001;33(5):350-57.

83. Simon JE, Docherty CL. Current Health-Related Quality of Life in Former National Collegiate Athletic Association Division I Collision Athletes Compared With Contact and Limited-Contact Athletes. *Journal of athletic training* 2016;51(3):205-12. doi: 10.4085/1062-6050-51.4.05 [published Online First: 2016/03/10]

84. Simon JE, Docherty CL. Current health-related quality of life is lower in former Division I collegiate athletes than in non-collegiate athletes. *The American journal of sports medicine* 2014;42(2):423-9. doi: 10.1177/0363546513510393 [published Online First: 2013/12/10]

85. Sorenson SC, Romano R, Scholefield RM, et al. Holistic life-span health outcomes among elite intercollegiate student-athletes. *Journal of athletic training* 2014;49(5):684-95. doi: 10.4085/1062-6050-49.3.18 [published Online First: 2014/08/15]

86. Gouttebarge V, Aoki H, Kerkhoffs G. Lower extremity osteoarthritis is associated with lower health-related quality of life among retired professional footballers. *The Physician and sportsmedicine* 2018;46(4):471-76. doi: 10.1080/00913847.2018.1451718 [published Online First: 2018/03/13]

87. Cameron KL, Thompson BS, Peck KY, et al. Normative values for the KOOS and WOMAC in a young athletic population: history of knee ligament injury is associated with lower scores. *The American journal of sports medicine* 2013;41(3):582-9. doi: 10.1177/0363546512472330 [published Online First: 2013/01/19]

88. McAllister DR, Motamedi AR, Hame SL, et al. Quality of life assessment in elite collegiate athletes. *The American journal of sports medicine* 2001;29(6):806-10. doi: 10.1177/03635465010290062201 [published Online First: 2001/12/06]

89. Valovich McLeod TC, Decoster LC, Loud KJ, et al. National Athletic Trainers' Association position statement: prevention of pediatric overuse injuries. *Journal of athletic training* 2011;46(2):206-20. doi: 10.4085/1062-6050-46.2.206 [published Online First: 2011/03/12]

90. Gardner AW, Montgomery PS, Wang M, et al. Predictors of health-related quality of life in patients with symptomatic peripheral artery disease. *Journal of vascular surgery* 2018;68(4):1126-34. doi: 10.1016/j.jvs.2017.12.074 [published Online First: 2018/04/05]

91. Zhao Y, Chung PK. Neighborhood environment walkability and health-related quality of life among older adults in Hong Kong. *Archives of gerontology and geriatrics* 2017;73:182-86. doi: 10.1016/j.archger.2017.08.003 [published Online First: 2017/08/22]

92. Ninkovic S, Avramov S, Harhaji V, et al. INFLUENCE OF DIFFERENT LEVELS OF SPORTS ACTIVITIES ON THE QUALITY OF LIFE AFTER THE RECONSTRUCTION OF ANTERIOR CRUCIATE LIGAMENT. *Medicinski pregled* 2015;68(3-4):116-21. [published Online First: 2015/07/29]

93. Royston P, Sauerbrei W. Building multivariable regression models with continuous covariates in clinical epidemiology. *Methods of information in medicine* 2005;44(04):561-71.

94. Brenner PS, DeLamater JD. Social desirability bias in self-reports of physical activity: is an exercise identity the culprit? *Social Indicators Research* 2014;117(2):489-504.

For peer review only - http://bmjopen.bmj.com/site/about/guidelines.xhtml

95. Crutzen R, Göritz AS. Does social desirability compromise self-reports of physical activity in web-based research? *International Journal of Behavioral Nutrition and Physical Activity* 2011;8(1):31.

96. Sesso R, Rodrigues-Neto JF, Ferraz MB. Impact of socioeconomic status on the quality of life of ESRD patients. *American Journal of Kidney Diseases* 2003;41(1):186-95.

97. Penson DF, Stoddard ML, Pasta DJ, et al. The association between socioeconomic status, health insurance coverage, and quality of life in men with prostate cancer. *Journal of clinical epidemiology* 2001;54(4):350-58.

For peer review only

FIGURE 1. Study Flow Chart

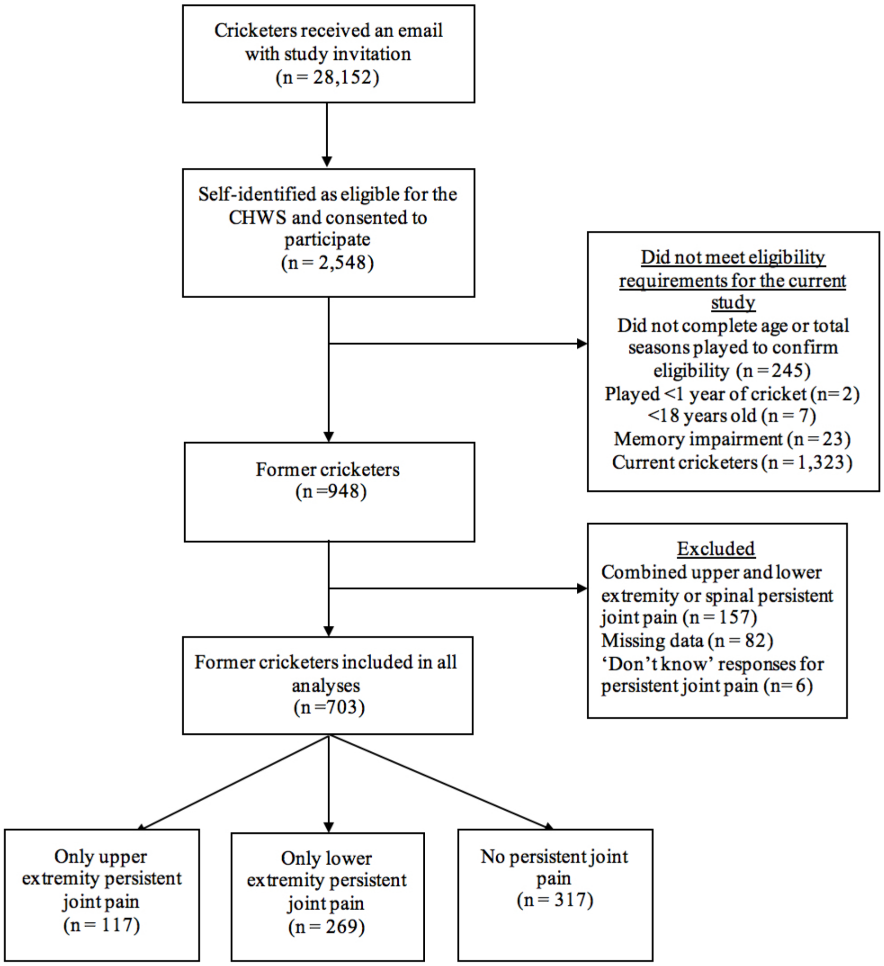

Figure 1. Study Flow Chart

196x210mm (144 x 144 DPI)

Figure 2: Weekly physical activity levels and health related quality of life in former cricketers with upper extremity persistent joint pain, lower extremity persistent joint pain, or no persistent joint pain

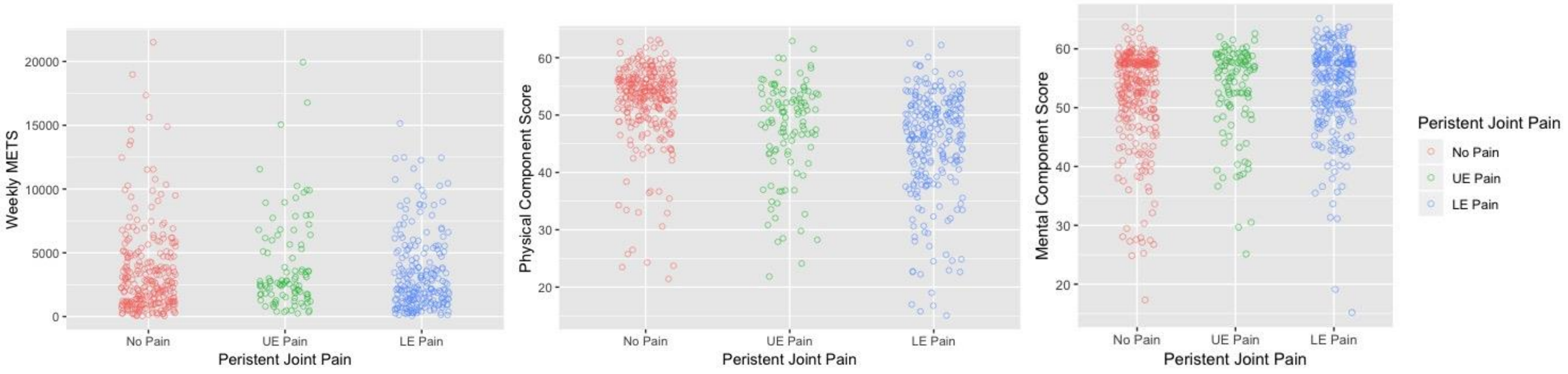

METS = Metabolic Equivalents, PCS = Physical Component Score, MCS = Mental Component Score, UE= Upper Extremity, LE=Lower Extremity

Appendix 1. Missing Data Responses in Former Cricketers

Variable	Count	Percentage
Physical Component Score	82	8.6%
Mental Component Score	80	8.4%
Body mass Index	61	6.3%
IPAQ-SF	56	5.9%
Cricket Seasons	12	1.7%
Persistent Pain	10	1.4%
Osteoarthritis	10	1.4%
Other Cancer	10	1.4%
Skin Cancer	8	1.1%
Stroke	8	1.1%
Diabetes	1	0.1%
Age	0	0%

For peer review only

STROBE Statement—checklist of items that should be included in reports of observational studies

	Item No	Recommendation
**Title and abstract**	1	(*a*) Indicate the study's design with a commonly used term in the title or the abstract **Page 1 and 3**
		(*b*) Provide in the abstract an informative and balanced summary of what was done and what was found **page 3**
**Introduction**		
Background/rationale	2	Explain the scientific background and rationale for the investigation being reported **Page 4**
Objectives	3	State specific objectives, including any prespecified hypotheses **page 5**
**Methods**		
Study design	4	Present key elements of study design early in the paper **page 5-6**
Setting	5	Describe the setting, locations, and relevant dates, including periods of recruitment, exposure, follow-up, and data collection **page 6**
Participants	6	(*a*) *Cohort study*—Give the eligibility criteria, and the sources and methods of selection of participants. Describe methods of follow-up<br>*Case-control study*—Give the eligibility criteria, and the sources and methods of case ascertainment and control selection. Give the rationale for the choice of cases and controls<br>*Cross-sectional study*—Give the eligibility criteria, and the sources and methods of selection of participants **page 5**
		(*b*) *Cohort study*—For matched studies, give matching criteria and number of exposed and unexposed<br>*Case-control study*—For matched studies, give matching criteria and the number of controls per case
Variables	7	Clearly define all outcomes, exposures, predictors, potential confounders, and effect modifiers. Give diagnostic criteria, if applicable **page 6-8**
Data sources/ measurement	8*	For each variable of interest, give sources of data and details of methods of assessment (measurement). Describe comparability of assessment methods if there is more than one group **page 7-8**
Bias	9	Describe any efforts to address potential sources of bias **page 8**
Study size	10	Explain how the study size was arrived at **figure 1**
Quantitative variables	11	Explain how quantitative variables were handled in the analyses. If applicable, describe which groupings were chosen and why **page 8**
Statistical methods	12	(*a*) Describe all statistical methods, including those used to control for confounding **page 8**
		(*b*) Describe any methods used to examine subgroups and interactions **page 8**
		(*c*) Explain how missing data were addressed **page 8-9**
		(*d*) *Cohort study*—If applicable, explain how loss to follow-up was addressed<br>*Case-control study*—If applicable, explain how matching of cases and controls was addressed<br>*Cross-sectional study*—If applicable, describe analytical methods taking account of sampling strategy **page 4-5**
		(*e*) Describe any sensitivity analyses 8-9

Continued on next page

For peer review only - http://bmjopen.bmj.com/site/about/guidelines.xhtml

**Results**		
Participants	13*	(a) Report numbers of individuals at each stage of study—eg numbers potentially eligible, examined for eligibility, confirmed eligible, included in the study, completing follow-up, and analysed **Figure 1**
		(b) Give reasons for non-participation at each stage **Figure 1**
		(c) Consider use of a flow diagram **Figure 1**
Descriptive data	14*	(a) Give characteristics of study participants (eg demographic, clinical, social) and information on exposures and potential confounders **Table 1**
		(b) Indicate number of participants with missing data for each variable of interest **Appendix 1**
		(c) *Cohort study*—Summarise follow-up time (eg, average and total amount)
Outcome data	15*	*Cohort study*—Report numbers of outcome events or summary measures over time
		Case-control study—Report numbers in each exposure category, or summary measures of exposure
		Cross-sectional study—Report numbers of outcome events or summary measures **page 10-11, Table 1**
Main results	16	(*a*) Give unadjusted estimates and, if applicable, confounder-adjusted estimates and their precision (eg, 95% confidence interval). Make clear which confounders were adjusted for and why they were included **page 10-11, Table 2-3 and figure 2**
		(*b*) Report category boundaries when continuous variables were categorized **page 10-11, Table 2, 3**
		(*c*) If relevant, consider translating estimates of relative risk into absolute risk for a meaningful time period **NA for these analyses**
Other analyses	17	Report other analyses done—eg analyses of subgroups and interactions, and sensitivity analyses 9-11
**Discussion**		
Key results	18	Summarise key results with reference to study objectives **page 12**
Limitations	19	Discuss limitations of the study, taking into account sources of potential bias or imprecision. Discuss both direction and magnitude of any potential bias **page 15**
Interpretation	20	Give a cautious overall interpretation of results considering objectives, limitations, multiplicity of analyses, results from similar studies, and other relevant evidence **page 16**
Generalisability	21	Discuss the generalisability (external validity) of the study results **page 15-16**
**Other information**		
Funding	22	Give the source of funding and the role of the funders for the present study and, if applicable, for the original study on which the present article is based **page 16**

*Give information separately for cases and controls in case-control studies and, if applicable, for exposed and unexposed groups in cohort and cross-sectional studies.

Note: An Explanation and Elaboration article discusses each checklist item and gives methodological background and published examples of transparent reporting. The STROBE checklist is best used in conjunction with this article (freely available on the Web sites of PLoS Medicine at http://www.plosmedicine.org/, Annals of Internal Medicine at http://www.annals.org/, and Epidemiology at http://www.epidem.com/). Information on the STROBE Initiative is available at www.strobe-statement.org.

For peer review only - http://bmjopen.bmj.com/site/about/guidelines.xhtml

BMJ Open

Physical activity and health related quality of life in former elite and recreational cricketers from the United Kingdom with upper extremity or lower extremity persistent joint pain: A cross-sectional study

Journal:	*BMJ Open*
Manuscript ID	bmjopen-2019-032606.R2
Article Type:	Original research
Date Submitted by the Author:	30-Sep-2019
Complete List of Authors:	Bullock, Garrett; University of Oxford, Nuffield Department of Orthopaedics, Rheumatology, and Musculoskeletal Sciences<br>Collins, Gary; University of Oxford, Centre for Statistics in Medicine<br>Peirce, Nicholas; Nottingham University Hospitals Trust, Centre For Sports Medicine; England and Wales Cricket Board, National Cricket Performance Centre<br>Arden, Nigel; Nuffield Department of Orthopaedics, ;<br>Filbay, Stephanie; University of Oxford, Arthritis Research UK Centre for Sport, Exercise & Osteoarthritis, Nuffield Department of Orthopaedics, Rheumatology & Musculoskeletal Sciences
**Primary Subject Heading**:	Sports and exercise medicine
Secondary Subject Heading:	Mental health, Public health
Keywords:	Shoulder < ORTHOPAEDIC & TRAUMA SURGERY, Knee < ORTHOPAEDIC & TRAUMA SURGERY, ankle, Hip < ORTHOPAEDIC & TRAUMA SURGERY, hand, retired athlete

SCHOLARONE™
Manuscripts

Physical activity and health related quality of life in former elite and recreational cricketers from the United Kingdom with upper extremity or lower extremity persistent joint pain: A cross-sectional study

Garrett S. Bullock PT, DPT,[1, 2] Gary S. Collins Bsc (Hons), PhD,[3,4] Peirce N., BMBS, FRIS,[5, 6] Nigel K. Arden, MD, FRCP,[1, 2] Stephanie R. Filbay, B.Phty (Hons), PhD[1, 2]

1. Centre for Sport, Exercise and Osteoarthritis Research *Versus Arthritis,* University of Oxford, United Kingdom
2. Nuffield Department of Orthopaedics, Rheumatology, and Musculoskeletal Sciences, University of Oxford, Oxford, United Kingdom
3. Centre for Statistics in Medicine, Nuffield Department of Orthopaedics, Rheumatology, and Musculoskeletal Sciences, University of Oxford, Oxford United Kingdom
4. Oxford University Hospitals NHS Foundation Trust, Oxford, United Kingdom
5. England and Wales Cricket Board, National Cricket Performance Centre, Loughborough University, LE11 3TU, United Kingdom
6. National Centre for Sport and Exercise Medicine, Loughborough University, LE11 3TU, United Kingdom

Corresponding Author Information

Garrett S. Bullock PT, DPT
Nuffield Department of Orthopaedics, Rheumatology, and Musculoskeletal Sciences
University of Oxford
B4495
Oxford, United Kingdom
OX3 7LD
(865) 227-374
garrett.bullock@wolfson.ox.ac.uk

Key Words: Shoulder, Knee, Ankle, Hip, Hand, Sport, Retired Athletes

Word Count: 3518

Conflict of Interest: Dr. Nick Peirce is affiliated with the England and Wales Cricket Board. All other authors affirm that they have no involvement with any commercial organization that has a direct financial interest in any matter included in this manuscript.

For peer review only

Abstract

Objectives: Evaluate and compare physical activity (PA) and health-related quality-of-life (HRQoL) in former elite and recreational cricketers with upper extremity (UE), lower extremity (LE), or no joint pain.

Study Design: Cross-sectional cohort

Setting: Despite the high prevalence of joint pain in former athletes, the impact of UE-pain and LE-pain on PA and HRQoL, and potential differences between former recreational and elite athletes, is poorly understood.

Participants: 703 former cricketers aged ≥18 years (aged mean 58.7 SD 12.9, played an average 30 IQR 20-40 seasons, 72% had played at a recreational level) were recruited through the Cricket Health and Wellbeing Study and met eligibility requirements (UE-pain, LE-pain or no joint pain (defined as pain on most days of the past month)).

Primary and Secondary Outcomes: A questionnaire collected IPAQ-SF (weekly metabolic equivalents (METS)) and SF-8 (physical (PCS) and mental (MCS) component scores). Kruskall-Wallis tests with Dunn's post-hoc and multivariable linear regressions were performed.

Results: Weekly METS were similar in former cricketers with UE-pain (median(IQR) 2560(722-4398)), LE-pain (2215(527-3903)) and no pain (2449(695-4203), p=0.39). MCS scores were similar between groups (UE-pain 56.0(52.1-60.0); LE-pain 55.2(51.1-59.4); no pain 54.7(50.7-58.7), p=0.38). PCS scores were more impaired in former cricketers with UE-pain (49.8(44.9-54.8)) or LE-pain (46.7(41.0-51.9)) compared to no pain (54.2(51.5-56.9), p<0.0001). Former cricketers with LE-pain reported worse PCS scores than those with UE-pain (p=0.04). Similar relationships were observed in former elite and recreational cricketers.

Conclusion: Despite impaired physical-components of HRQoL in former cricketers with UE-pain or LE-pain, pain was not related to PA levels or mental-components of HRQoL. Physical-components of HRQoL was most impaired in those with LE-pain, and findings were similar amongst former elite and recreational cricketers.

Key Words: Shoulder, Knee, Ankle, Hip, Hand, Sport, Retired Athletes

Strengths and Limitations:

- This study took into account the non-linear relationships between different continuous variables (i.e. by using fractional polynomials), where most studies assume linearity.
- Missing data was low, allowing for low bias in a complete case analysis.
- Participants with persistent pain in both the upper and lower extremities, and participants with back pain were excluded from the analyses, decreasing the generalizability of these findings.
- The SF-8 minimal detectable difference and minimal clinical important difference literature is sparse, and does not evaluate athletic populations. This decreases the interpretability of these data.

For peer review only - http://bmjopen.bmj.com/site/about/guidelines.xhtml

INTRODUCTION

Upper and lower extremity osteoarthritis (OA) has a high incidence[1 2] and a substantial personal and societal burden.[3 4] Post-traumatic OA occurs at a younger age, with a longer period of disability and less effective treatments than idiopathic OA.[5-7] Former sport participants have increased post-traumatic OA prevalence compared to the general population,[5 8] which is associated with persistent joint pain.[9] Persistent joint pain is the major cause of patients seeking medical attention for OA,[9] and has been shown to influence a person's ability to participate in desired forms of activity[10 11] which may result in reduced physical activity levels and negatively impact health-related quality of life (HRQoL).[10-13]

To date, most research investigating OA and pain has focused on the lower extremity.[10-14] Former professional soccer players were found to have a high prevalence of lower extremity OA and pain compared to controls,[10 11] while former female recreational athletes were found to have 2 to 3 times increased hip and knee OA risk compared to population matched controls.[14] Lower extremity OA and pain in former sport participants is associated with impaired function and mobility, increased levels of anxiety and depression, and reduced HRQoL compared to the general population.[10 12-14]

Upper extremity pain can have different barriers to PA participation compared to lower extremity pain.[15-18] For example, lower extremity pain may have a greater effect on jumping, stair use, walking and running.[19 20] In contrast, upper extremity pain can have more effect on specific functional activities such as dressing and eating[15] or work related tasks such as carrying and handling objects[16] with potential negative impacts on HRQoL.[15] Upper extremity pain has been related to reduced PA and function, impaired HRQoL, stress and disability in workers.[21-25] Despite a high prevelance of upper extremity pain in former

For peer review only

For peer review only - http://bmjopen.bmj.com/site/about/guidelines.xhtml

throwing-sport participants,[26-28] the impact of upper extremity pain on PA levels and HRQoL in former athletes is poorly understood.

Psychological characteristics associated with successful high level athletic performance include resilience, mental toughness and advanced coping skills.[29-32] Greater resilience and coping strategies can have positive impact on PA levels[33-35] and HRQoL.[34 36-38] Thus, former elite athletes may possess psychological characteristics that enable them to better cope with chronic pain compared to former recreational sport participants, and this could positiviely impact PA levels and HRQoLHowever, this has not yet been investigated, highlighting the need for further research. It is also not clear if PA levels and HRQoL differ in former sport participants with and without perisistent joint pain. The rationale for investigating these relationships in former cricketers was largely due to the high prevalence of upper and lower extremity osteoarthritis and persistent pain amongst former cricketers,[12 39] and our previous qualitative work highlighting that a subgroup of former cricketers experience high quality of life and maintain physically active lifestyles despite persistent joint pain.[40 41] Therefore, the purpose of this study was to:

1) evaluate the relationship between persistent joint pain (upper extremity, lower extremity, or no persistent joint pain), PA levels and HRQoL in former cricketers;

2) compare these relationships in former elite and recreational cricketer subgroups.

METHODS

Study Design

This cross-sectional study was approved by the NHS Health Research Authority (NRES), London Stanmore Research Ethics Committee (REC 15/LO/1274).

For peer review only

Patient and public involvement

Findings from two qualitative studies investigating the relationship of physical activity and quality of life in former elite cricketers,[40 41] highlighted a need for further research investigating the relationship between cricket participation and health. In collaboration with stakeholders, including governing bodies and current and former cricketers, a questionnaire was developed and refined. This information was disseminated through presentations to stakeholders.

Participants and recruitment

In March 2017, 28,152 current and former cricketers of all playing standards, who were registered on a national database managed by the England and Wales Cricket Board, received an email inviting them to complete an electronic questionnaire. 2,598 people believed they met the eligibility requirements outlined in the email, and consented to participate in the Cricket Health and Wellbeing Study (Figure 1). Participants were eligible for inclusion in the Cricket Health and Wellbeing Study if they had played ≥1 cricket season and were aged ≥18 years. Due to the potential confounding relationship between acute injury and joint pain,[42] only former cricketers were included in this study. The other eligibility requirement for inclusion in this study, was reporting either i) persistent joint pain in only the upper extremity, ii) persistent joint pain in only the lower extremity, or iii) no persistent joint pain (individuals were excluded if they reported persistent back pain, or combined upper and lower extremity persistent joint pain). Participants were also excluded if they reported any memory impairment.

Questionnaire design

For peer review only

The Cricket Health and Wellbeing Study questionnaire was developed in collaboration with public involvement and four current/former cricketers piloted the questionnaire. The questionnaire was created to evaluate five aspects of health and wellbeing (i. cricket-related injury; ii. joint pain and osteoarthritis; iii. general health and disease prevalence; iv. physical activity; v. resilience, quality of life and flourishing). All participant data was managed and de-identified in a RedCap® (Research Electronic Data Capture) software database. The RedCap® database was managed by one investigator, and pilot tested by three researchers for errors and overall userbility.[43 44] The RedCap® software[44] utilized branching logic and allowed participants the option to save their current progress and complete at a later time. The questionnaire captured data including cricketers' age, body mass index (BMI), number of cricket seasons played, playing standard, presence of comorbidities, persistent pain, medically diagnosed OA, physical activity and HRQoL.

<Figure 1>

Outcomes

Physical activity

The International Physical Activity Questionnaire-Short Form (IPAQ-SF) was utilized to assess physical activity.[45] The IPAQ-SF has been used as a population surveillance and evaluation PA tool.[46 47] The IPAQ-SF has been observed to have fair agreement with accelerometer data (0.03-0.39)[48 49] and good validity compared to other PA questionnaires and PA logs (r>0.50).[49 50] Participants were asked to recall their PA in a usual week, as time spent in vigorous-intensity PA, moderate-intensity PA, and walking.[45] To reduce variability, a standardized approach outlined in the IPAQ-SF manual was utilized to clean and code the IPAQ-SF data.[45] In line with recommendations, bouts of weekly PA less than 10 minutes were recoded as zero minutes.[45] Time spent in vigorous PA, moderate PA, and walking was

For peer review only - http://bmjopen.bmj.com/site/about/guidelines.xhtml

converted to Metabolic Equivalents (METS). One MET is 1 kcal/kg/h, or the resting metabolic rate, during quiet sitting.[45] Vigorous PA was calculated as 8 METS per min, moderate PA was calculated as 4 METS per min, and walking was calculated as 3.3 METS per min.[45] Metabolic equivalents were truncated at three hours (180 minutes) per week, per vigorous, moderate, and walking PA.[45] This was performed in order to reduce participant overestimation effect.[45] A total of 117 (17%) of participants had PA reports truncated. METS were then combined for vigorous PA, moderate PA, and walking, and reported as total weekly METS.

Health related quality of life

The Short-Form 8 (SF-8) was used to assess HRQoL.[51] The SF-8 is a short version of the RAND 36-Item Health Survey (SF-36) 1.0,[52] the SF-8 is scaled and measured on the same point scale (0-100) as the SF-36, with 0 representing maximum disability and 100 representing no disability.[53] The SF-8 is an 8-item, self-reported HRQoL questionnaire comprising 8 domains (general health perceptions, physical function, bodily pain, physical role function, emotional role function, social function, vitality, and mental health)[53] and two component summary scores (the Physical Component Score (PCS) and Mental Component Score (MCS)). The PCS and MCS scores have been found to have high reliability (0.88 and 0.82 respectively) in the general United States population.[54] The PCS and MCS scores are calculated using a computerized algorithm, and weighted to the United States population mean.[54] A score of 50 is considered the population norm, and a score below 47 is considered below the average range of the general population.[54] The minimum detectable difference for the PCS in lower extremity OA patients is two points.[55] The minimally clinically important difference is estimated to be three to five points in the United States general population.[56]

For peer review only - http://bmjopen.bmj.com/site/about/guidelines.xhtml

Explanatory Variables

Persistent joint pain

Persistent joint pain was assessed with the following question, *'Do you currently experience pain, discomfort, or have any problems in any of your joints?'* If yes, participants were then asked *'Have you had pain in your [left/right] hip/groin, knee, ankle, shoulder, hand/finger, spine/back, other joint on most days of the last month?'*. All participants that recorded persistent pain in the "other joint" category were hand searched for references of elbow, wrist, foot, or toe persistent joint pain. These data were then recorded into separate categories of elbow/wrist, and foot/toes persistent joint pain. All persistent joint pain data was then separated into only upper extremity persistent joint pain (shoulder, elbow, wrist, hand and/or fingers), only lower extremity persistent joint pain (hip, knee, ankle, foot, and/or toes), and no persistent joint pain. If participants recorded spinal persistent pain, or upper and lower extremity persistent joint pain, subjects were excluded from the analyses.

Standard of play

Standard-of-play was assessed with the following question, '*what was the highest standard of cricket that you played for at least one season*?' Response options included: international; county/premier league; academy or county age group; university; school; village or social; don't know. Participants were stratified into recreational (university, school, village or social) and elite (international or county/premier league, academy or county age group). Participants that reported a playing standard as *'Don't know'* were excluded from all analyses.

Confounders

Potential confounders were identified through clinical reasoning and a review of the literature. Confounders included age, body mass index (BMI), and presence of a comorbidity.

For peer review only - http://bmjopen.bmj.com/site/about/guidelines.xhtml

Participants reported all medical problems that they had been diagnosed with. For the current study, diabetes, stroke and cancer were included as confounders due to a large potential to impact HRQoL and PA levels.[57-61] Presence of a comorbidity was defined as no comorbidity vs. one or more comorbidity.

Statistical Analyses

All data were assessed for missingness prior to analyses. Missing data were calculated as total and percentage of total data (Appendix 1). Due to the low percentage of missing data (MCS: 8.6%, PCS: 8.4%, IPAQ-SF: 5.9%, persistent joint pain: 1.1%), a complete case analysis was performed. 'Don't know' responses were excluded from all analyses. The Kruskall Wallis test was used to compare PA and HRQoL, between pain groups. To assess specific group to group differences, a series of Dunn's post hoc analyses were performed ($p<0.05$).

Continuous data were not assumed to act linearly on the outcomes. Thus, to evaluate the relationship between persistent joint pain, PA levels and HRQoL, multivariable linear regressions with fractional polynomials were used (to account for the non-linearity of the covariates).[62] This analysis was repeated in recreational and elite cricketer subgroups to address the second aim of this study. Unadjusted and adjusted coefficients and 95% confidence intervals (95% CI) were calculated. Coefficients were adjusted for the continuous variables age and BMI, and the presence of ≥1 comorbidity (yes vs. no). All assumptions for fractional polynomial regression were evaluated and satisfied.[62] Transformations included taking the square root of METS, PCS and MCS scores were not transformed due to regression residuals. Possible interactions were assessed for inclusion in the model. Interactions explored were between age and BMI, age and comorbidity, BMI and

For peer review only

comorbidity, age and persistent pain, BMI and persistent pain, and comorbidity and persistent pain. All analyses were performed in R version 3.5.1 (R Core Team (2013). R: A language and environment for statistical computing. R Foundation for Statistical Computing, Vienna, Austria. URL http://www.R-project.org/), using the dplyr package[63] for cleaning and coding, the naniar package for missingness assessment,[64] and the mfp package for fractional polynomial regression.[65]

RESULTS

A total of 703 former cricketers (aged mean 58.7 SD 12.9, played an average 30 IQR 20-40 seasons, 38% had played at an elite level) were included in analyses (Table 1). Body mass index in people with upper extremity persistent joint pain was 27.9 (SD 5.0), lower extremity persistent joint pain 29.1 (SD 5.5), and in people with no persistent joint pain was 27.8 (SD 5.4).

TABLE 1. Participant characteristics

	All former cricketers (n = 703)	Upper extremity persistent joint pain (n = 117)	Lower extremity persistent joint pain (n = 269)	No persistent joint pain (n = 317)
Age (years)	58.7 (SD 12.9)	59.9 (SD 12.6)	59.8 (SD 11.8)	57.8 (SD 13.8)
Sex				
Male	683 (97%)	113 (97%)	263 (98%)	307 (97%)
Female	15 (3%)	3 (3%)	4 (2%)	8 (3%)
NR	5 (<1%)	1 (<1%)	2 (<1%)	2 (<1%)
BMI (kg/m2)	28.3 (SD 5.3)	27.9 (SD 5.0)	29.1 (SD 5.5)	27.8 (SD 5.4)
Seasons Played	30 (IQR 20-40)	35 (IQR 27-44)	32 (IQR 23-42)	30 (IQR 18-48)
Years since last cricket match	7.4 (IQR 0.4-14.4)	8.1 (IQR 0.8-15.4)	6.9 (IQR 0.1-13.7)	6.7 (IQR 0.1-13.3)
Playing standard				
Elite	264 (38%)	46 (39%)	109 (41%)	109 (34%)
Recreational	428 (61%)	68 (58%)	156 (58%)	204 (65%)
NR	11 (1%)	3 (3%)	4 (1%)	4 (1%)
Comorbidity				
No	497 (73%)	81 (74%)	186 (71%)	230 (75%)
Yes	155 (23%)	23 (21%)	64 (25%)	68 (22%)
NR	27 (4%)	6 (5%)	10 (4%)	11 (4%)

For peer review only - http://bmjopen.bmj.com/site/about/guidelines.xhtml

SD = Standard Deviation, IQR = Inter Quartile Range, NR= No Response, BMI = Body Mass Index, [a]Participants reported the highest standard they had played for at least one season and then were stratified into recreational (university, school, village or social) and elite (international or county/premier league, academy or county age group)

A comparison of physical activity and HRQoL between former cricketers with upper extremity persistent joint pain, lower extremity persistent joint pain, and no persistent joint pain

For a visual representation of the PA data, please refer to Figure 2. Kruskall Wallis test indicated that there was no difference in physical activity levels between former cricketers with upper extremity persistent joint pain (Median (IQR): 2560 (722-4398) METS), lower extremity persistent joint pain (2215 (527-3903) METS), or no persistent joint pain (2449 (695-4203) METS) (Chi-square: 1.91, p=0.39). There were significant PCS differences between pain groups (p<0.001). Post hoc tests demonstrated that former cricketers reported worse PCS scores if they had persistent joint pain in the upper extremity (PCS: 49.8 (44.9-54.8)) or lower extremity (PCS: 46.7 (41.0-51.9)), compared to former cricketers with no persistant joint pain (PCS: 54.2 (51.5-56.9); Chi-square:121.2, p<0.001). Former cricketers with lower extremity persistent joint pain reported worse PCS scores compared to former cricketers with upper extremity persistent joint pain (Median Difference: 1.8, p=0.04). MCS scores were similar between individuals with upper extremity persistent joint pain (56.0 (52.1-60.0)), lower extremity persistent joint pain (55.2 (51.1-59.4)), and no persistent joint pain (54.7 (50.7-58.7)); Chi-squared: 1.92, p=0.38).

<Figure 2>

The relationship between persistent joint pain, physical activity and health related quality of life

Multivariable linear regressions found that persistent joint pain was not related to physical activity levels (Upper Extremity: Adjusted effect (95% CI): 28.1 METS (-1.1 to 135.7); Lower Extremity: 4.6 METS (-7.5 to 49.4)), see Table 2. Former cricketers with upper extremity persistent joint pain reported an estimated -5.5 (-7.4 to -3.5) points lower (worse) PCS scores than cricketers without persistent joint pain. Former cricketers with lower extremity persistent joint pain reported an estimated -6.6 (-8.1 to -5.2) points lower PCS scores compared to those with no persistent joint pain (Table 2). Persistent joint pain was not associated with MCS scores (Upper Extremity: -0.1 (-0.2 to 0.1); Lower Extremity: -0.1 (-0.2 to 0.1)).

For peer review only

TABLE 2. The relationship between persistent joint pain, physical activity and health related quality of life

	Weekly METS		PCS score		MCS score	
	Unadjusted Effect[b] (95% CI)	Adjusted[a] Effect[b] (95% CI)	Unadjusted Effect[c] (95% CI)	Adjusted[a] Effect[c] (95% CI)	Unadjusted Effect[c] (95% CI)	Adjusted[a] Effect[c] (95% CI)
Upper extremity persistent joint pain	20.3 (-3.2, 117), p=0.83	28.1 (-1.1, 136), p=0.10	-5.4 (-7.3, -3.6), p<0.001	-5.5 (-7.4, -3.5), p<0.001	-0.1 (-0.2, 0.1), p=0.35	-0.1 (-0.2, 0.1), p=0.26
Lower extremity persistent joint pain	0.3 (-12.5, 21.0), p=0.16	4.6 (-7.5, 49.4), p=0.39	-6.9 (-8.4, -5.5), p<0.001	-6.6 (-8.1, -5.2), p<0.001	0.09 (-0.2, 0.1), p=0.08	-0.1 (-0.2, 0.1), p=0.06
No persistent joint pain	*Reference group*		*Reference group*		*Reference group*	

[a] Estimates are adjusted for age, body mass index, and comorbidities
[b] Participants with memory impairments were excluded from the analyses
[c] Comorbidities were defined as none present (0) and presence of at least one comorbidity (1). Comorbidities included were diabetes, stroke, skin cancer, and other cancer
[d] Upper extremity (shoulder, elbow, wrist, hand) and lower extremity (hip, knee, ankle) persistent joint pain were assessed by asking individuals if they had joint-specific pain on 'most days of the last month.'
[e] Questionnaire Short-Form (IPAQ-SF); Physical activity was calculated as metabolic equivalents (METS) per week; METS were transformed prior to analysis by taking the square root and then retransformed by squaring after analysis
[f] SF-8: Short-Form 8 Health Survey; Physical Component Scores (PCS) and Mental Component Scores (MCS) were calculated using norm based scoring (population norm 50 SD 10, high scorer = better health-related quality of life)

Elite vs. recreational cricketer subgroup analysis

Multivariable linear regressions found that findings were similar between recreational and elite cricketer subgroups (Table 3). Persistent joint pain was not associated with physical activity and MCS scores, and PCS scores were impaired in both former elite and recreational cricketers with upper extremity (Adjusted effect (95% CI) Elite: -5.3 (-8.8, -1.9); Recreational: -5.8 (-8.1, -3.4)) or lower extremity (Elite: -7.0 (-9.7, -4.3); Recreational: -6.2 (-8.0, -4.5)) persistent joint pain, compared to their counterparts with no persistent joint pain (Table 3).

For peer review only - http://bmjopen.bmj.com/site/about/guidelines.xhtml

TABLE 3. The relationship between persistent joint pain, physical activity and health related quality of life, in former recreational and elite cricketer subgroups

		Weekly METS		PCS score		MCS score	
Former elite cricketers (n=280)		Unadjusted Effect[b] (95% CI)	Adjusted[a] Effect[b] (95% CI)	Unadjusted Effect[c] (95% CI)	Adjusted[a] Effect[c] (95% CI)	Unadjusted Effect[c] (95% CI)	Adjusted[a] Effect[c] (95% CI)
	Upper extremity persistent joint pain	70 (-1.6, 324), p=0.09	88 (-0.1, 356), p=0.05	-5.3 (-8.6, -2.0), p=0.001	-5.3 (-8.8, -1.9), p=0.002	0.055 (-2.3, 3.4), p=0.70	5.3 (-1.1, 11.7), p=0.10
	Lower extremity persistent joint pain	5.2 (-28, 97), p=0.56	8.2 (-21, 106), p=0.45	-7.5 (-10.1, -4.9), p<0.001	-7.0 (-9.7, -4.3), p<0.001	0.1 (-1.7, 2.7), p=0.64	2.2 (-2.7, 7.0), p=0.39
	No persistent joint pain	*Reference group*		*Reference group*		*Reference group*	
Former recreational cricketers (n=423)		Unadjusted Effect[b] (95% CI)	Adjusted[a] Effect[b] (95% CI)	Unadjusted Effect[c] (95% CI)	Adjusted[a] Effect[c] (95% CI)	Unadjusted Effect[c] (95% CI)	Adjusted[a] Effect[c] (95% CI)
	Upper extremity persistent joint pain	5.6 (-37, 117), p=0.58	10.5 (-29, 149), p=0.47	-5.6 (-7.9, -3.3), p<0.001	-5.8 (-8.1, -3.4), p<0.001	0.8 (-1.5, 3.0), p=0.50	1.1 (-1.1, 3.2), p=0.35
	Lower extremity persistent joint pain	-0.7 (-49, 29), p=0.83	1.8 (-26, 61), p=0.68	-6.6 (-8.3, -4.9), p<0.001	-6.2 (-8.0, -4.5), p<0.001	-0.8 (-1.1, 2.7), p=0.33	1.2 (-0.5, 2.8), p=0.17

No persistent joint pain	*Reference group*	*Reference group*	*Reference group*

[a] Estimates are adjusted for age, body mass index, and comorbidities
[b] Participants with memory impairments were excluded from the analyses
[c] Comorbidities were defined as not present (0) and presence of at least one comorbidity (1). Comorbidities included were diabetes, stroke, skin cancer, and other cancer
[e] Upper extremity (shoulder, elbow, wrist, or hand) and lower extremity (hip, knee, or ankle) persistent joint pain were assessed by asking individuals if they had joint-specific pain on 'most days of the last month.'
[f] Questionnaire Short-Form (IPAQ-SF); Physical activity was calculated as metabolic equivalents (METS) per week; METS were transformed prior to analysis by taking the square root and then retransformed by squaring after analysis
[g] SF-8: Short-Form 8 Health Survey; Physical Component Scores (PCS) were calculated using norm based scoring (population norm 50 SD 10, high scorer = better health-related quality of life); Mental Component Scores (MCS) were calculated using norm based scoring (population norm 50 SD 10, high scorer = better health-related quality of life)

For peer review only

For peer review only - http://bmjopen.bmj.com/site/about/guidelines.xhtml

DISCUSSION

Suprisingly, despite impaired PCS scores, there was no difference in weekly PA or MCS scores, between former cricketers with upper or lower extremity persistent joint pain, and those with no persistent joint pain. Additionally, former cricketers with lower extremity persistent joint pain reported worse physical-components of HRQoL than those with upper extremity persistent joint pain. We also found that the relationship between joint pain, PA and HRQoL were similar amougnst former elite and recreational cricketer subgroups.

Former cricketers with persistent joint pain did not report reduced weekly PA compared to former cricketers without persistent joint pain. In the general population, people with OA have reduced PA levels compared to matched controls without OA.[66] In this study, former cricketers with and without persistent joint pain had higher PA levels compared to the general population.[67-69] Former cricketers reported median daily PA levels of 120 METS per day, this is in comparison to an average range of 41 to 50 METS per day for European men.[67-69] Former cricketers may find alternative strategies to exercise, despite persistent joint pain.[41 70] In a qualitative study, former cricketers with pain and physical impairment found alternative strategies to perform PA, and shared specific psychological strengths that may enable them to effectively cope with joint pain.[41] Intrinsic motivation,[71] higher levels of resilience,[32 40] and effective coping strategies[40 41] are all characteristics common amongst successful athletes. Such psychological strengths may be more common in former sport participants compared to the general population.[29-32] Additionally, sport participants may be encouraged to play through pain[72] and are often rewarded for doing so.[73] Such pain behaviours could influence how former sport participants perceive and cope with joint pain. Pain behaviours and psychological strengths common amougnst sport participants could provide a potential explanation for the high physical activity levels in former cricketers living with joint pain.

For peer review only

For peer review only - http://bmjopen.bmj.com/site/about/guidelines.xhtml

Further research is required to understand the potential interplay between persistent pain, psychological characteristics, and PA in former athletes.

The psychological strengths that are common amoungst former sports participants including resilience,[32 40] mental toughness,[74 75] and pain coping strategies,[40 75] could also provide a potential explanation for why MCS scores were not impaired in former cricketers with persistent joint pain, despite impaired PCS scores. In a recent meta-analysis, former athletes with impaired PCS scores reported greater MCS scores compared to general population norms.[76] In a typical OA population, both PCS and MCS scores are impaired,[77] suggesting HRQoL in former sport participants with OA may differ from the typical OA population. Furthermore, the high PA levels in former cricketers in our study could also contribute to the high MCS scores. Physical activity has been shown to have a protective effect against depression and a positive impact on mental-components of HRQoL in the general population, older adults, and in former elite athletes.[78-81] Irrespective of joint pain, former cricketers reported MCS scores 5 to 6 points better than the population normative average, and this difference is likely to be clinically meaningful.[82]

Former cricketers with persistent joint pain had impaired physical-components of HRQoL compared to cricketers without persistent pain. This supports previous literature which found impaired PCS scores in former collegiate[83-85] and professional athletes[86] compared to non-athlete controls. Former cricketers with persistent pain reported PCS scores 5 to 7 points lower than former cricketers without persistent pain, demonstrating a clinical important difference between persistent pain and no pain groups. Impaired physical-components of HRQoL in former athletes has been attributed to poor joint health,[83-85] which may be due to previous musculoskeletal injury.[87-89] Former athletes with a history of musculoskeletal injury

For peer review only

For peer review only - http://bmjopen.bmj.com/site/about/guidelines.xhtml

report worse physical-components of HRQoL, than former athletes without a history of injury[87] and the general population.[88 89] In our study, former cricketers without persistent pain reported a median PCS score 4 points above the population normative mean of 50, suggesting that former cricket participants without joint pain have greater levels of physical functioning compared to the general population. Importantly, despite impaired PCS scores in former cricketers with persistent pain, MCS scores were not impaired. This highlights the importance of selecting a HRQoL measure that differentiates assessment of pain and physical function, from assessment of mental health and wellbeing, when evaluating HRQoL in former sport participants.

Cricketers with persistent lower extremity pain reported 3.1 points worse PCS scores compared to cricketers with upper extremity pain, and this difference is likely to be clinically meaningful.[82] Worse PCS scores in former cricketers with lower extremity pain may be due to greater difficulties performing usual physical activities (such as difficulties with ambulation and stair navigation[90 91]) or experiencing more severe bodily pain than those who report upper extremity pain. For example, former collegiate athletes with lower extremity persistent pain were 2.5 times more likely to be limited in physical function, such as stair navigation, and have greater impaired HRQoL, compared to non-athletes.[84] While there is research investigating the impacts of lower extremity pain on function in former athletes, our understanding of the impact of upper extremity pain on function in former athletes is poor. Further research could inform strategies to improve physical-components of HRQoL amongst former athletes with persistent upper extremity pain.

The relationship between persistent joint pain, PA, and HRQoL was similar between elite and recreational former cricketers. Few studies have investigated PA or HRQoL in former

For peer review only - http://bmjopen.bmj.com/site/about/guidelines.xhtml

athletes from different standards-of-play. Higher standards of sport competition requires elevated levels of resilience and psychological hardiness due to the increased levels of training and competition.[29-32 92] Despite the observed psychological strengths associated with elite cricket participation, any level of sport participation is associated with favourable psychological strengths such as resilience.[30 32 92] Thus, it is possible that all standards of cricket participation is associated with psychological benefits that may explain the high PA levels and mental-components of HRQoL in former cricketers living with persistant pain; further research is needed to explore this possibility.

Strengths and potential limitations

This study took into account the non-linear relationships between different continuous variables (i.e. by using fractional polynomials), where most studies assume linearity.[93] Missing data was low, decreasing the risk of bias in a complete case analysis. Physical activity was truncated above 180 minutes, which may introduce a ceiling effect. Further, PA recall may not be as prcise as PA monitors, which may introduce measurement error, and social desirability may cause PA overestimation.[94 95] Persistent joint pain was assessed by asking cricketers if they had joint pain on most days of the last month, which is based on the National health and Nutrition Examination Survey (NHANES). However, joint pain may occur intermittently, and still effect PA and HRQoL. As a result, this study may have missed participants with significant pain. Participants with persistent pain in both the upper and lower extremities, and participants with back pain were excluded from the analyses. This limits the scope of this study to participants with only upper or lower extremity persistent pain, or no persistent pain, decreasing the generalisability of these findings. The SF-8 minimal detectable difference and minimal clinical important difference literature is sparse, and does not evaluate athletic populations. This decreases the interpretability of these data.

For peer review only - http://bmjopen.bmj.com/site/about/guidelines.xhtml

There is also the possibility of bias due to unmeasured confounding. Potential confounders that were not measured in this study include socio-economic status and other sports played, which both have been observed to be related to HRQoL.[76 96 97] These discrepancies may decrease the generalisability of these findings. Potential participants were informed of the inclusion and exclusion criteria during recruitment, and were able to self-select eligibility to participate. Due to this recruitment strategy, it is not possible to determine the questionnaire response rate, nor responder bias. This recruitment strategy may decrease the generalisabilty of these findings.

Conclusions

Physical components of HRQoL were impaired in former cricketers with persistent joint pain compared to those without joint pain, and PCS scores were more impaired in those with lower extremity pain compared to those with upper extremity pain. However, former cricketers with persistent joint pain did not have reduced PA levels or mental components of HRQoL, compared to those with no persistent joint pain. These relationships were similar amongst former elite and recreational cricketers. These results highlight the need to select a HRQoL measure that differentiates assessment of pain and physical functional, from assessment of mental health and wellbeing, when evaluating HRQoL in former sport participants. The high average physical activity levels amongst former cricketers with persistent joint pain could explain the high MCS scores in this group. In contrast, former cricketers with joint pain who are physically inactive may benefit from targeted strategies to increase activity levels, with potential to positively impact QOL. Further research is needed to determine whether the psychological benefits of sport participation explain why former cricketers had high PA levels and mental-components of HRQoL, despite living with persistant joint-pain.

For peer review only - http://bmjopen.bmj.com/site/about/guidelines.xhtml

Author's Contributions

GSB, GSC, NP, NKA, SRF conceived the study idea. GSB, GSC, NP, NKA, SRF were involved in methodological design and planning. GSB and SRF wrote the first draft of the manuscript. GSB, GSC, SRF contributed to statistical analyses. GSB, GSC, NP, NKA, SRF all critically revised the manuscript. GSB, GSC, NP, NKA, SRF all approved the final version of the manuscript.

Funding: This study was funded by Centre for Sport, Exercise and Osteoarthritis Research Versus Arthritis (grant reference 21595). Gary Collins was supported by the NIHR Biomedical Research Centre, Oxford.

Data Availability

Data is available upon request. Please contact the authors for further inquiry.

Figure and Table Captions

Figure 1. Study Flow Chart

Table 1. Participant Characteristics

Figure 2. Weekly physical activity levels and health related quality of life in former cricketers with upper extremity persistent joint pain, lower extremity persistent joint pain, or no persistent joint pain

Table 2. The relationship between persistent joint pain, physical activity and health related quality of life

Table 3. The relationship between persistent joint pain, physical activity and health related quality of life, in former recreational and elite cricketer subgroups

For peer review only - http://bmjopen.bmj.com/site/about/guidelines.xhtml

For peer review only

References

1. Plotnikoff R, Karunamuni N, Lytvyak E, et al. Osteoarthritis prevalence and modifiable factors: a population study. *BMC public health* 2015;15:1195. doi: 10.1186/s12889-015-2529-0 [published Online First: 2015/12/02]
2. van Schoor NM, Zambon S, Castell MV, et al. Impact of clinical osteoarthritis of the hip, knee and hand on self-rated health in six European countries: the European Project on OSteoArthritis. *Quality of life research : an international journal of quality of life aspects of treatment, care and rehabilitation* 2016;25(6):1423-32. doi: 10.1007/s11136-015-1171-8 [published Online First: 2015/11/09]
3. Palazzo C, Nguyen C, Lefevre-Colau MM, et al. Risk factors and burden of osteoarthritis. *Annals of physical and rehabilitation medicine* 2016;59(3):134-38. doi: 10.1016/j.rehab.2016.01.006 [published Online First: 2016/02/26]
4. Cross M, Smith E, Hoy D, et al. The global burden of hip and knee osteoarthritis: estimates from the global burden of disease 2010 study. *Annals of the rheumatic diseases* 2014;73(7):1323-30. doi: 10.1136/annrheumdis-2013-204763 [published Online First: 2014/02/21]
5. Carbone A, Rodeo S. Review of current understanding of post-traumatic osteoarthritis resulting from sports injuries. *Journal of orthopaedic research : official publication of the Orthopaedic Research Society* 2017;35(3):397-405. doi: 10.1002/jor.23341 [published Online First: 2016/06/17]
6. Brown TD, Johnston RC, Saltzman CL, et al. Posttraumatic osteoarthritis: a first estimate of incidence, prevalence, and burden of disease. *Journal of orthopaedic trauma* 2006;20(10):739-44. doi: 10.1097/01.bot.0000246468.80635.ef [published Online First: 2006/11/16]
7. Vannini F, Spalding T, Andriolo L, et al. Sport and early osteoarthritis: the role of sport in aetiology, progression and treatment of knee osteoarthritis. *Knee surgery, sports traumatology, arthroscopy : official journal of the ESSKA* 2016;24(6):1786-96. doi: 10.1007/s00167-016-4090-5 [published Online First: 2016/04/05]
8. Whittaker JL, Woodhouse LJ, Nettel-Aguirre A, et al. Outcomes associated with early post-traumatic osteoarthritis and other negative health consequences 3-10 years following knee joint injury in youth sport. *Osteoarthritis and cartilage* 2015;23(7):1122-9. doi: 10.1016/j.joca.2015.02.021 [published Online First: 2015/03/01]
9. Neogi T. The epidemiology and impact of pain in osteoarthritis. *Osteoarthritis and cartilage* 2013;21(9):1145-53. doi: 10.1016/j.joca.2013.03.018 [published Online First: 2013/08/27]
10. Arliani GG, Astur DC, Yamada RK, et al. Early osteoarthritis and reduced quality of life after retirement in former professional soccer players. *Clinics (Sao Paulo, Brazil)* 2014;69(9):589-94. [published Online First: 2014/10/16]
11. Turner AP, Barlow JH, Heathcote-Elliott C. Long term health impact of playing professional football in the United Kingdom. *British journal of sports medicine* 2000;34(5):332-6. [published Online First: 2000/10/26]
12. Jones ME, Davies MAM, Leyland KM, et al. Osteoarthritis and other long-term health conditions in former elite cricketers. *Journal of science and medicine in sport* 2018;21(6):558-63. doi: 10.1016/j.jsams.2017.10.013 [published Online First: 2017/11/02]
13. Davies MAM, A DJ, Delmestri A, et al. Health amongst former rugby union players: A cross-sectional study of morbidity and health-related quality of life. *Scientific reports* 2017;7(1):11786. doi: 10.1038/s41598-017-12130-y [published Online First: 2017/09/30]
14. Spector TD, Harris PA, Hart DJ, et al. Risk of osteoarthritis associated with long-term weight-bearing sports: a radiologic survey of the hips and knees in female ex-athletes and population controls. *Arthritis and rheumatism* 1996;39(6):988-95. [published Online First: 1996/06/01]

For peer review only

15. Lang CE, Bland MD, Bailey RR, et al. Assessment of upper extremity impairment, function, and activity after stroke: foundations for clinical decision making. *Journal of hand therapy : official journal of the American Society of Hand Therapists* 2013;26(2):104-14;quiz 15. doi: 10.1016/j.jht.2012.06.005 [published Online First: 2012/09/15]
16. Shaw WS, Feuerstein M, Lincoln AE, et al. Ergonomic and psychosocial factors affect daily function in workers' compensation claimants with persistent upper extremity disorders. *Journal of occupational and environmental medicine* 2002;44(7):606-15. [published Online First: 2002/07/24]
17. Peters SE, Johnston V, Ross M, et al. Expert consensus on facilitators and barriers to return-to-work following surgery for non-traumatic upper extremity conditions: a Delphi study. *The Journal of hand surgery, European volume* 2017;42(2):127-36. doi: 10.1177/1753193416669263 [published Online First: 2016/09/23]
18. Hundza S, Quartly C, Kim JM, et al. Similar barriers and facilitators to physical activity across different clinical groups experiencing lower limb spasticity. *Disability and rehabilitation* 2016;38(14):1370-81. doi: 10.3109/09638288.2015.1101789 [published Online First: 2016/01/05]
19. Barnes KR, McGuigan MR, Kilding AE. Lower-body determinants of running economy in male and female distance runners. *Journal of strength and conditioning research* 2014;28(5):1289-97. doi: 10.1519/jsc.0000000000000267 [published Online First: 2013/10/16]
20. Vogt MT, Lauerman WC, Chirumbole M, et al. A community-based study of postmenopausal white women with back and leg pain: health status and limitations in physical activity. *The journals of gerontology Series A, Biological sciences and medical sciences* 2002;57(8):M544-50. [published Online First: 2002/07/30]
21. Montero A, Mulero JF, Tornero C, et al. Pain, disability and health-related quality of life in osteoarthritis-joint matters: an observational, multi-specialty trans-national follow-up study. *Clin Rheumatol* 2016;35(9):2293-305. doi: 10.1007/s10067-016-3248-3 [published Online First: 2016/04/14]
22. Verma C, Parikh R, Nadkar M, et al. Correlation of Functional Ability of the Hand with Upper Limb Function and Quality of Life in Patients with Rheumatoid Arthritis. *J Assoc Physicians India* 2017;65(3):20-24. [published Online First: 2017/05/04]
23. Rincon-Hurtado AM, Rocha-Buelvas A, Lopez-Cardona A, et al. Health-related quality of life of patients with rotator cuff injuries, Cofee Triangle, Colombia, 2013. *Rev Bras Ortop* 2018;53(3):364-72. doi: 10.1016/j.rboe.2018.03.018 [published Online First: 2018/06/13]
24. Cassou B, Derriennic F, Monfort C, et al. Chronic neck and shoulder pain, age, and working conditions: longitudinal results from a large random sample in France. *Occup Environ Med* 2002;59(8):537-44. [published Online First: 2002/08/02]
25. Grzywacz JG, Arcury TA, Mora D, et al. Work organization and musculoskeletal health: clinical findings from immigrant Latino poultry processing and other manual workers. *J Occup Environ Med* 2012;54(8):995-1001. doi: 10.1097/JOM.0b013e318254640d [published Online First: 2012/07/24]
26. Sauers EL, Dykstra DL, Bay RC, et al. Upper extremity injury history, current pain rating, and health-related quality of life in female softball pitchers. *Journal of sport rehabilitation* 2011;20(1):100-14. [published Online First: 2011/03/18]
27. Soomro N, Strasiotto L, Sawdagar T, et al. Cricket Injury Epidemiology in the Twenty-First Century: What is the Burden? *Sports medicine (Auckland, NZ)* 2018;48(10):2301-16. doi: 10.1007/s40279-018-0960-y [published Online First: 2018/07/19]
28. Posner M, Cameron KL, Wolf JM, et al. Epidemiology of Major League Baseball injuries. *The American journal of sports medicine* 2011;39(8):1676-80. doi: 10.1177/0363546511411700 [published Online First: 2011/06/29]
29. Cowden RG, Fuller DK, Anshel MH. Psychological predictors of mental toughness in elite tennis: an exploratory study in learned resourcefulness and competitive trait anxiety. *Perceptual*

For peer review only - http://bmjopen.bmj.com/site/about/guidelines.xhtml

and motor skills 2014;119(3):661-78. doi: 10.2466/30.PMS.119c27z0 [published Online First: 2014/11/12]
30. Secades XG, Molinero O, Salguero A, et al. Relationship Between Resilience and Coping Strategies in Competitive Sport. *Perceptual and motor skills* 2016;122(1):336-49. doi: 10.1177/0031512516631056 [published Online First: 2016/07/16]
31. Shields MR, Brooks MA, Koltyn KF, et al. Cognitive Resilience and Psychological Responses across a Collegiate Rowing Season. *Medicine and science in sports and exercise* 2017;49(11):2276-85. doi: 10.1249/mss.0000000000001363 [published Online First: 2017/07/07]
32. Sarkar M, Fletcher D. Psychological resilience in sport performers: a review of stressors and protective factors. *Journal of sports sciences* 2014;32(15):1419-34. doi: 10.1080/02640414.2014.901551 [published Online First: 2014/04/11]
33. Wermelinger Avila MP, Correa JC, Lucchetti ALG, et al. The Role of Physical Activity in the Association Between Resilience and Mental Health in Older Adults. *Journal of aging and physical activity* 2018;26(2):248-53. doi: 10.1123/japa.2016-0332 [published Online First: 2017/07/18]
34. Taylor K, Scruggs PW, Balemba OB, et al. Associations between physical activity, resilience, and quality of life in people with inflammatory bowel disease. *European journal of applied physiology* 2018;118(4):829-36. doi: 10.1007/s00421-018-3817-z [published Online First: 2018/02/08]
35. Seaton CL, Bottorff JL, Jones-Bricker M, et al. The Role of Positive Emotion and Ego-Resilience in Determining Men's Physical Activity Following a Workplace Health Intervention. *American journal of men's health* 2018;12(6):1916-28. doi: 10.1177/1557988318803744 [published Online First: 2018/10/20]
36. Paffenbarger RS, Jr., Lee IM, Leung R. Physical activity and personal characteristics associated with depression and suicide in American college men. *Acta psychiatrica Scandinavica Supplementum* 1994;377:16-22. [published Online First: 1994/01/01]
37. Camacho TC, Roberts RE, Lazarus NB, et al. Physical activity and depression: evidence from the Alameda County Study. *American journal of epidemiology* 1991;134(2):220-31. [published Online First: 1991/07/15]
38. Farmer ME, Locke BZ, Moscicki EK, et al. Physical activity and depressive symptoms: the NHANES I Epidemiologic Follow-up Study. *American journal of epidemiology* 1988;128(6):1340-51. [published Online First: 1988/12/01]
39. Jones ME, Davies MA, Leyland KM, et al. The association of previous injury with joint pain, osteoarthritis, and joint replacement across all joints in former elite English cricketers. *Osteoarthritis and Cartilage* 2017;25 (Supplement 1):S200-S01.
40. Filbay SR, Bishop F, Peirce N, et al. Common attributes in retired professional cricketers that may enhance or hinder quality of life after retirement: a qualitative study. *BMJ open* 2017;7(7):e016541. doi: 10.1136/bmjopen-2017-016541 [published Online First: 2017/07/29]
41. Filbay SR, Bishop FL, Peirce N, et al. Physical activity in former elite cricketers and strategies for promoting physical activity after retirement from cricket: a qualitative study. *BMJ open* 2017;7(11):e017785. doi: 10.1136/bmjopen-2017-017785 [published Online First: 2017/11/21]
42. Igolnikov I, Gallagher RM, Hainline B. Sport-related injury and pain classification. *Handbook of clinical neurology* 2018;158:423-30. doi: 10.1016/b978-0-444-63954-7.00039-2 [published Online First: 2018/11/30]
43. Harris PA, Taylor R, Thielke R, et al. Research electronic data capture (REDCap)--a metadata-driven methodology and workflow process for providing translational research informatics support. *Journal of biomedical informatics* 2009;42(2):377-81. doi: 10.1016/j.jbi.2008.08.010 [published Online First: 2008/10/22]

For peer review only

For peer review only - http://bmjopen.bmj.com/site/about/guidelines.xhtml

44. Lyon JA, Garcia-Milian R, Norton HF, et al. The use of Research Electronic Data Capture (REDCap) software to create a database of librarian-mediated literature searches. *Medical reference services quarterly* 2014;33(3):241-52. doi: 10.1080/02763869.2014.925379 [published Online First: 2014/07/16]

45. Committee IR. Guidelines for data processing and analysis of the International Physical Activity Questionnaire (IPAQ)-short and long forms. *http://www ipaq ki se/scoring pdf* 2005

46. Rosemann T, Kuehlein T, Laux G, et al. Osteoarthritis of the knee and hip: a comparison of factors associated with physical activity. *Clinical rheumatology* 2007;26(11):1811-7. doi: 10.1007/s10067-007-0579-0 [published Online First: 2007/03/03]

47. Hultenheim Klintberg I, Karlsson J, Svantesson U. Health-related quality of life, patient satisfaction, and physical activity 8-11 years after arthroscopic subacromial decompression. *Journal of shoulder and elbow surgery* 2011;20(4):598-608. doi: 10.1016/j.jse.2010.08.021 [published Online First: 2010/12/15]

48. Ekelund U, Sepp H, Brage S, et al. Criterion-related validity of the last 7-day, short form of the International Physical Activity Questionnaire in Swedish adults. *Public health nutrition* 2006;9(2):258-65.

49. Mader U, Martin BW, Schutz Y, et al. Validity of four short physical activity questionnaires in middle-aged persons. *Medicine and science in sports and exercise* 2006;38(7):1255-66. doi: 10.1249/01.mss.0000227310.18902.28 [published Online First: 2006/07/11]

50. Macfarlane DJ, Lee CC, Ho EY, et al. Convergent validity of six methods to assess physical activity in daily life. *Journal of applied physiology (Bethesda, Md : 1985)* 2006;101(5):1328-34. doi: 10.1152/japplphysiol.00336.2006 [published Online First: 2006/07/11]

51. Turner-Bowker DM, Bayliss MS, Ware JE, Jr., et al. Usefulness of the SF-8 Health Survey for comparing the impact of migraine and other conditions. *Quality of life research : an international journal of quality of life aspects of treatment, care and rehabilitation* 2003;12(8):1003-12. [published Online First: 2003/12/04]

52. Hays RD, Sherbourne CD, Mazel RM. The RAND 36-Item Health Survey 1.0. *Health economics* 1993;2(3):217-27. [published Online First: 1993/10/01]

53. Ware JE, Jr., Sherbourne CD. The MOS 36-item short-form health survey (SF-36). I. Conceptual framework and item selection. *Medical care* 1992;30(6):473-83. [published Online First: 1992/06/11]

54. Ware JE, Kosinski M, Dewey JE, et al. How to score and interpret single-item health status measures: a manual for users of the SF-8 health survey. *Lincoln, RI: QualityMetric Incorporated* 2001;15(10):5.

55. Angst F, Aeschlimann A, Stucki G. Smallest detectable and minimal clinically important differences of rehabilitation intervention with their implications for required sample sizes using WOMAC and SF-36 quality of life measurement instruments in patients with osteoarthritis of the lower extremities. *Arthritis Care & Research: Official Journal of the American College of Rheumatology* 2001;45(4):384-91.

56. Samsa G, Edelman D, Rothman ML, et al. Determining clinically important differences in health status measures. *Pharmacoeconomics* 1999;15(2):141-55.

57. Mols F, Vingerhoets AJ, Coebergh JW, et al. Quality of life among long-term breast cancer survivors: a systematic review. *European journal of cancer* 2005;41(17):2613-19.

58. Doyle C, Kushi LH, Byers T, et al. Nutrition and physical activity during and after cancer treatment: an American Cancer Society guide for informed choices. *CA: a cancer journal for clinicians* 2006;56(6):323-53.

59. Manson JE, Stampfer M, Colditz G, et al. Physical activity and incidence of non-insulin-dependent diabetes mellitus in women. *The Lancet* 1991;338(8770):774-78.

60. Goldney RD, Phillips PJ, Fisher LJ, et al. Diabetes, depression, and quality of life: a population study. *Diabetes care* 2004;27(5):1066-70.

For peer review only - http://bmjopen.bmj.com/site/about/guidelines.xhtml

61. Moore SA, Hallsworth K, Plötz T, et al. Physical activity, sedentary behaviour and metabolic control following stroke: a cross-sectional and longitudinal study. *PloS one* 2013;8(1):e55263.
62. Royston P, Altman DG. Regression using fractional polynomials of continuous covariates: parsimonious parametric modelling. *Journal of the Royal Statistical Society: Series C (Applied Statistics)* 1994;43(3):429-53.
63. Hadley Wickham RF, Lionel Henry, Kirill Muller. dplyr: A Grammar of Data Manipulation 2018 [R package version 0.7.6.:[Available from: https://CRAN.R-project.org/package=dplyr.
64. Nicholas Tierney DC, Miles McBain, Colin Fay, Mitchell O'Hara-Wild, Jim Hester, Luke Smith. naniar 2018 [R Package version: 0.4.1:[Available from: https://cran.r-project.org/web/packages/naniar/index.html.
65. Gareth Ambler AB. mfp. R package 1.5.2 ed2015.
66. Wallis JA, Webster KE, Levinger P, et al. What proportion of people with hip and knee osteoarthritis meet physical activity guidelines? A systematic review and meta-analysis. *Osteoarthritis and cartilage* 2013;21(11):1648-59. doi: 10.1016/j.joca.2013.08.003 [published Online First: 2013/08/21]
67. Aadahl M, Kjaer M, Jorgensen T. Associations between overall physical activity level and cardiovascular risk factors in an adult population. *European journal of epidemiology* 2007;22(6):369-78. doi: 10.1007/s10654-006-9100-3 [published Online First: 2007/03/03]
68. Schaller N, Seiler H, Himmerich S, et al. Estimated physical activity in Bavaria, Germany, and its implications for obesity risk: results from the BVS-II Study. *International Journal of Behavioral Nutrition and Physical Activity* 2005;2(1):6.
69. Norman A, Bellocco R, Bergström A, et al. Validity and reproducibility of self-reported total physical activity—differences by relative weight. *International journal of obesity* 2001;25(5):682.
70. Holden MA, Nicholls EE, Young J, et al. Exercise and physical activity in older adults with knee pain: a mixed methods study. *Rheumatology (Oxford, England)* 2015;54(3):413-23. doi: 10.1093/rheumatology/keu333 [published Online First: 2014/09/05]
71. Teixeira PJ, Carraça EV, Markland D, et al. Exercise, physical activity, and self-determination theory: a systematic review. *International journal of behavioral nutrition and physical activity* 2012;9(1):78.
72. Hammond LE, Lilley JM, Pope GD, et al. 'We've just learnt to put up with it': an exploration of attitudes and decision-making surrounding playing with injury in English professional football. *Qualitative Research in Sport, Exercise and Health* 2014;6(2):161-81.
73. Roderick M, Waddington I, Parker G. Playing hurt: Managing injuries in English professional football. *International review for the sociology of sport* 2000;35(2):165-80.
74. Meggs J, Ditzfeld C, Golby J. Self-concept organisation and mental toughness in sport. *Journal of sports sciences* 2014;32(2):101-9. doi: 10.1080/02640414.2013.812230 [published Online First: 2013/08/24]
75. Nicholls AR, Polman RCJ, Levy AR, et al. Mental toughness, optimism, pessimism, and coping among athletes. *Personality and Individual Differences* 2008;44(5):1182-92. doi: https://doi.org/10.1016/j.paid.2007.11.011
76. FIlbay SR PT, Thomas B, McKay C, Adams J, Arden NK. Quality of life and life satisfaction in former athletes: a systematic review and meta-analysis. *Sports Medicine* 2019. In Press.
77. Kobayashi S, Pappas E, Fransen M, et al. The prevalence of patellofemoral osteoarthritis: a systematic review and meta-analysis. *Osteoarthritis and cartilage* 2016;24(10):1697-707. doi: 10.1016/j.joca.2016.05.011 [published Online First: 2016/05/18]
78. Harvey SB, Overland S, Hatch SL, et al. Exercise and the Prevention of Depression: Results of the HUNT Cohort Study. *The American journal of psychiatry* 2018;175(1):28-36. doi: 10.1176/appi.ajp.2017.16111223 [published Online First: 2017/10/04]

For peer review only - http://bmjopen.bmj.com/site/about/guidelines.xhtml

79. Salmon P. Effects of physical exercise on anxiety, depression, and sensitivity to stress: a unifying theory. *Clinical psychology review* 2001;21(1):33-61. [published Online First: 2001/01/10]

80. Vasiliadis HM, Belanger MF. The prospective and concurrent effect of exercise on health related quality of life in older adults over a 3 year period. *Health and quality of life outcomes* 2018;16(1):15. doi: 10.1186/s12955-018-0843-9 [published Online First: 2018/01/18]

81. Bäckmand H, Kaprio J, Kujala U, et al. Influence of physical activity on depression and anxiety of former elite athletes. *International journal of sports medicine* 2003;24(08):609-19.

82. Hays RD, Morales LS. The RAND-36 measure of health-related quality of life. *Annals of medicine* 2001;33(5):350-57.

83. Simon JE, Docherty CL. Current Health-Related Quality of Life in Former National Collegiate Athletic Association Division I Collision Athletes Compared With Contact and Limited-Contact Athletes. *Journal of athletic training* 2016;51(3):205-12. doi: 10.4085/1062-6050-51.4.05 [published Online First: 2016/03/10]

84. Simon JE, Docherty CL. Current health-related quality of life is lower in former Division I collegiate athletes than in non-collegiate athletes. *The American journal of sports medicine* 2014;42(2):423-9. doi: 10.1177/0363546513510393 [published Online First: 2013/12/10]

85. Sorenson SC, Romano R, Scholefield RM, et al. Holistic life-span health outcomes among elite intercollegiate student-athletes. *Journal of athletic training* 2014;49(5):684-95. doi: 10.4085/1062-6050-49.3.18 [published Online First: 2014/08/15]

86. Gouttebarge V, Aoki H, Kerkhoffs G. Lower extremity osteoarthritis is associated with lower health-related quality of life among retired professional footballers. *The Physician and sportsmedicine* 2018;46(4):471-76. doi: 10.1080/00913847.2018.1451718 [published Online First: 2018/03/13]

87. Cameron KL, Thompson BS, Peck KY, et al. Normative values for the KOOS and WOMAC in a young athletic population: history of knee ligament injury is associated with lower scores. *The American journal of sports medicine* 2013;41(3):582-9. doi: 10.1177/0363546512472330 [published Online First: 2013/01/19]

88. McAllister DR, Motamedi AR, Hame SL, et al. Quality of life assessment in elite collegiate athletes. *The American journal of sports medicine* 2001;29(6):806-10. doi: 10.1177/03635465010290062201 [published Online First: 2001/12/06]

89. Valovich McLeod TC, Decoster LC, Loud KJ, et al. National Athletic Trainers' Association position statement: prevention of pediatric overuse injuries. *Journal of athletic training* 2011;46(2):206-20. doi: 10.4085/1062-6050-46.2.206 [published Online First: 2011/03/12]

90. Gardner AW, Montgomery PS, Wang M, et al. Predictors of health-related quality of life in patients with symptomatic peripheral artery disease. *Journal of vascular surgery* 2018;68(4):1126-34. doi: 10.1016/j.jvs.2017.12.074 [published Online First: 2018/04/05]

91. Zhao Y, Chung PK. Neighborhood environment walkability and health-related quality of life among older adults in Hong Kong. *Archives of gerontology and geriatrics* 2017;73:182-86. doi: 10.1016/j.archger.2017.08.003 [published Online First: 2017/08/22]

92. Ninkovic S, Avramov S, Harhaji V, et al. INFLUENCE OF DIFFERENT LEVELS OF SPORTS ACTIVITIES ON THE QUALITY OF LIFE AFTER THE RECONSTRUCTION OF ANTERIOR CRUCIATE LIGAMENT. *Medicinski pregled* 2015;68(3-4):116-21. [published Online First: 2015/07/29]

93. Royston P, Sauerbrei W. Building multivariable regression models with continuous covariates in clinical epidemiology. *Methods of information in medicine* 2005;44(04):561-71.

94. Brenner PS, DeLamater JD. Social desirability bias in self-reports of physical activity: is an exercise identity the culprit? *Social Indicators Research* 2014;117(2):489-504.

95. Crutzen R, Göritz AS. Does social desirability compromise self-reports of physical activity in web-based research? *International Journal of Behavioral Nutrition and Physical Activity* 2011;8(1):31.

96. Sesso R, Rodrigues-Neto JF, Ferraz MB. Impact of socioeconomic status on the quality of life of ESRD patients. *American Journal of Kidney Diseases* 2003;41(1):186-95.

For peer review only

For peer review only - http://bmjopen.bmj.com/site/about/guidelines.xhtml

97. Penson DF, Stoddard ML, Pasta DJ, et al. The association between socioeconomic status, health insurance coverage, and quality of life in men with prostate cancer. *Journal of clinical epidemiology* 2001;54(4):350-58.

For peer review only

FIGURE 1. Study Flow Chart

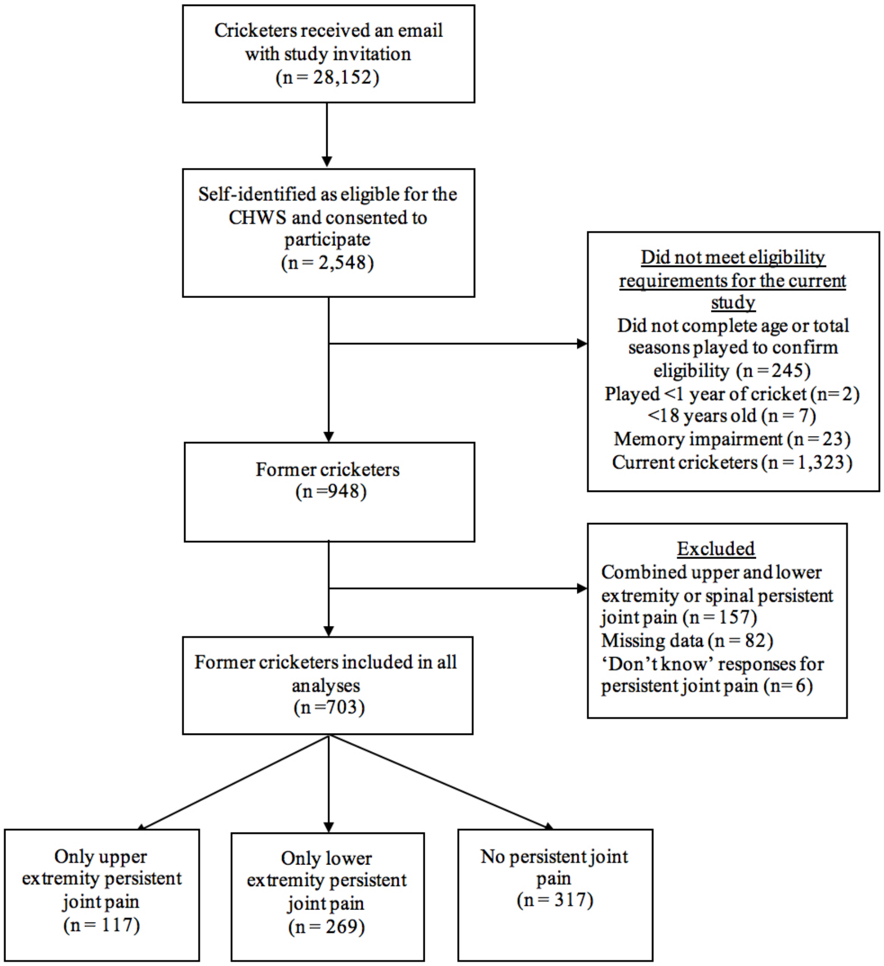

Figure 1. Study Flow Chart

196x210mm (144 x 144 DPI)

Figure 2: Weekly physical activity levels and health related quality of life in former cricketers with upper extremity persistent joint pain, lower extremity persistent joint pain, or no persistent joint pain

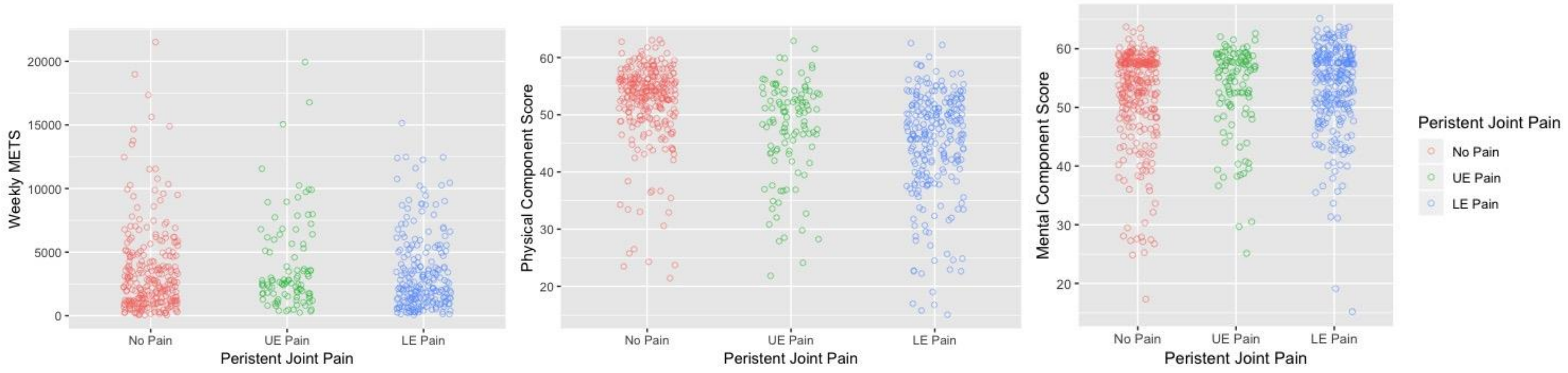

METS = Metabolic Equivalents, PCS = Physical Component Score, MCS = Mental Component Score, UE= Upper Extremity, LE=Lower Extremity

Appendix 1. Missing Data Responses in Former Cricketers

Variable	Count	Percentage
Physical Component Score	82	8.6%
Mental Component Score	80	8.4%
Body mass Index	61	6.3%
IPAQ-SF	56	5.9%
Cricket Seasons	12	1.7%
Persistent Pain	10	1.4%
Osteoarthritis	10	1.4%
Other Cancer	10	1.4%
Skin Cancer	8	1.1%
Stroke	8	1.1%
Diabetes	1	0.1%
Age	0	0%

For peer review only

STROBE Statement—checklist of items that should be included in reports of observational studies

	Item No	Recommendation
**Title and abstract**	1	(*a*) Indicate the study's design with a commonly used term in the title or the abstract **Page 1 and 3**
		(*b*) Provide in the abstract an informative and balanced summary of what was done and what was found **page 3**
**Introduction**		
Background/rationale	2	Explain the scientific background and rationale for the investigation being reported **Page 4**
Objectives	3	State specific objectives, including any prespecified hypotheses **page 5**
**Methods**		
Study design	4	Present key elements of study design early in the paper **page 5-6**
Setting	5	Describe the setting, locations, and relevant dates, including periods of recruitment, exposure, follow-up, and data collection **page 6**
Participants	6	(*a*) *Cohort study*—Give the eligibility criteria, and the sources and methods of selection of participants. Describe methods of follow-up<br>*Case-control study*—Give the eligibility criteria, and the sources and methods of case ascertainment and control selection. Give the rationale for the choice of cases and controls<br>*Cross-sectional study*—Give the eligibility criteria, and the sources and methods of selection of participants **page 5**
		(*b*) *Cohort study*—For matched studies, give matching criteria and number of exposed and unexposed<br>*Case-control study*—For matched studies, give matching criteria and the number of controls per case
Variables	7	Clearly define all outcomes, exposures, predictors, potential confounders, and effect modifiers. Give diagnostic criteria, if applicable **page 6-8**
Data sources/ measurement	8*	For each variable of interest, give sources of data and details of methods of assessment (measurement). Describe comparability of assessment methods if there is more than one group **page 7-8**
Bias	9	Describe any efforts to address potential sources of bias **page 8**
Study size	10	Explain how the study size was arrived at **figure 1**
Quantitative variables	11	Explain how quantitative variables were handled in the analyses. If applicable, describe which groupings were chosen and why **page 8**
Statistical methods	12	(*a*) Describe all statistical methods, including those used to control for confounding **page 8**
		(*b*) Describe any methods used to examine subgroups and interactions **page 8**
		(*c*) Explain how missing data were addressed **page 8-9**
		(*d*) *Cohort study*—If applicable, explain how loss to follow-up was addressed<br>*Case-control study*—If applicable, explain how matching of cases and controls was addressed<br>*Cross-sectional study*—If applicable, describe analytical methods taking account of sampling strategy **page 4-5**
		(*e*) Describe any sensitivity analyses 8-9

Continued on next page

For peer review only - http://bmjopen.bmj.com/site/about/guidelines.xhtml

For peer review only

**Results**		
Participants	13*	(a) Report numbers of individuals at each stage of study—eg numbers potentially eligible, examined for eligibility, confirmed eligible, included in the study, completing follow-up, and analysed **Figure 1**
		(b) Give reasons for non-participation at each stage **Figure 1**
		(c) Consider use of a flow diagram **Figure 1**
Descriptive data	14*	(a) Give characteristics of study participants (eg demographic, clinical, social) and information on exposures and potential confounders **Table 1**
		(b) Indicate number of participants with missing data for each variable of interest **Appendix 1**
		(c) *Cohort study*—Summarise follow-up time (eg, average and total amount)
Outcome data	15*	*Cohort study*—Report numbers of outcome events or summary measures over time
		Case-control study—Report numbers in each exposure category, or summary measures of exposure
		Cross-sectional study—Report numbers of outcome events or summary measures **page 10-11, Table 1**
Main results	16	(*a*) Give unadjusted estimates and, if applicable, confounder-adjusted estimates and their precision (eg, 95% confidence interval). Make clear which confounders were adjusted for and why they were included **page 10-11, Table 2-3 and figure 2**
		(*b*) Report category boundaries when continuous variables were categorized **page 10-11, Table 2, 3**
		(*c*) If relevant, consider translating estimates of relative risk into absolute risk for a meaningful time period **NA for these analyses**
Other analyses	17	Report other analyses done—eg analyses of subgroups and interactions, and sensitivity analyses 9-11
**Discussion**		
Key results	18	Summarise key results with reference to study objectives **page 12**
Limitations	19	Discuss limitations of the study, taking into account sources of potential bias or imprecision. Discuss both direction and magnitude of any potential bias **page 15**
Interpretation	20	Give a cautious overall interpretation of results considering objectives, limitations, multiplicity of analyses, results from similar studies, and other relevant evidence **page 16**
Generalisability	21	Discuss the generalisability (external validity) of the study results **page 15-16**
**Other information**		
Funding	22	Give the source of funding and the role of the funders for the present study and, if applicable, for the original study on which the present article is based **page 16**

*Give information separately for cases and controls in case-control studies and, if applicable, for exposed and unexposed groups in cohort and cross-sectional studies.

Note: An Explanation and Elaboration article discusses each checklist item and gives methodological background and published examples of transparent reporting. The STROBE checklist is best used in conjunction with this article (freely available on the Web sites of PLoS Medicine at http://www.plosmedicine.org/, Annals of Internal Medicine at http://www.annals.org/, and Epidemiology at http://www.epidem.com/). Information on the STROBE Initiative is available at www.strobe-statement.org.

For peer review only - http://bmjopen.bmj.com/site/about/guidelines.xhtml
