# Supplementary material for: Physical activity and health-related quality of life in former elite and recreational cricketers from the UK with upper extremity or lower extremity persistent joint pain: a cross-sectional study
Source: BMJ Open. 2019 Nov 11;9(11):e032606. doi: 10.1136/bmjopen-2019-032606 (PMC6858171; doi:10.1136/bmjopen-2019-032606)
Supplement: Reviewer comments [file bmjopen-2019-032606.reviewer_comments.pdf]

PEER REVIEW HISTORY

BMJ Open publishes all reviews undertaken for accepted manuscripts. Reviewers are asked to complete a checklist review form (**http://bmjopen.bmj.com/site/about/resources/checklist.pdf**) and are provided with free text boxes to elaborate on their assessment. These free text comments are reproduced below.

ARTICLE DETAILS

**TITLE (PROVISIONAL)**	Physical activity and health related quality of life in former elite and recreational cricketers from the United Kingdom with upper extremity or lower extremity persistent joint pain: A cross-sectional study
**AUTHORS**	Bullock, Garrett; Collins, Gary; Peirce, Nicholas; Arden, Nigel; Filbay, Stephanie

VERSION 1 – REVIEW

**REVIEWER**	Dr Jonathan Quicke<br>Keele University, United Kingdom
**REVIEW RETURNED**	15-Aug-2019

**GENERAL COMMENTS**	Physical activity and health related quality of life in former elite and recreational cricketers with upper extremity or lower extremity persistent joint pain<br><br>Thank you for submitting your paper “Physical activity and health related quality of life in former elite and recreational cricketers with upper extremity or lower extremity persistent joint pain” to BMJ Open.<br><br>In my opinion, the BMJ Open readership with their broad medical research interest may be interested in this paper. The standard of scientific writing was generally strong and clear throughout and there are novel elements in the work. The authors gained ethical approval.<br><br>Aims/ Background<br>The authors introduce the health context of their paper by discussing the link between persistent joint pain in former sports participants and OA. They also report that persistent joint pain in recreational and elite former sports participants may reduce physical activity levels and quality of life. The authors make the case that elite sports participants may possess unique psychological characteristics that may have a positive impact on their physical activity levels and quality of life in the presence of persistent pain when compared to recreational sport participants. Using a sample of former cricketers they evaluate the relationship between persistent joint pain (upper extremity, lower extremity or no persistent pain), physical activity levels and health-related quality of life in former recreational and elite cricketers.<br>In order to aid the flow of the introduction into the methods it may be helpful to add a sentence explaining why cricketers were the selected sample to address the research questions.<br>Typo on page 4, line 121 mental “toughness”

	Methods and analysis<br>A cross-sectional cohort design was used. Statistical methods used for analyses appear appropriate.<br>From the participants and recruitment section it is difficult to know if the response rate is low because the vast majority of the sample frame did not match the inclusion criteria (for example many will have been current players) or if this represents some kind of responder bias. This is worthy of discussion in the limitations section and may affect the generalisability of the findings.<br>Was there likely to be any unmeasured confounders that could have affected your relationships of interest? If so this could added to the discussion.<br>Is it possible that truncating moderate and vigorous physical activity at 180 minutes could produce a ceiling effect in your analysis given that this is a formerly active sample including elite athletes? Was this truncation applied to many participants?<br><br>Results<br>Results were clear. As a journal style point, for consistency, 4 decimal places may not be required for p-values.<br><br>Discussion and conclusion<br>To what extent do you think that measurement error and ceiling effect of your truncated IPAQ SF may have affected your findings?<br>Whilst self-report PA measures may be appropriate for measuring PA at single time points in large samples are there any limitations of self-report measures of PA that could be discussed briefly in the discussion? For example recall bias, social desirability bias and measurement error.<br>Page 15 line 390. In my opinion, it is incorrect to say that low levels of missing data “allowed for low bias in the findings” with complete case analysis. I would consider softening this sentence to talk about “risk of bias”. Reasons for missingness as well as the amount of missing data affect risk of bias.<br>Extra emphasis could be offered on why your findings are of clinical importance.

**REVIEWER**	Takeshi Toyooka<br>Nishikawa orthopaedic Clinic
**REVIEW RETURNED**	23-Aug-2019

**GENERAL COMMENTS**	This article is a very interesting study. There is some points to be worried about how to write statistics. Is the post hoc test results in Table 2 or figure 2? If so, the p-value should be clearly shown in a table or figure where and where compared not only effect size. Additionally, since the unit of the numerical value is described as METS on the line 286 on page 10, isn't this number an effect size? Line 275 on page 10, the result of the difference is presented. Is this also the result of post hoc test? Line 286 on page 10, although you show us " Lower Extremity: 20.3 METS" in the main body, the number is shown as Upper extremity persistent joint pain on table 2. which is correct result? The word "similar" "associate" shold not be used as a result of ANOVA. Finally, did you use two way ANOVA to analyze table 3? It is easier to understand if the statistics used to describe each result are described.

VERSION 1 – AUTHOR RESPONSE

Reviewer: 1
Reviewer Name: Dr Jonathan Quicke
Institution and Country: Keele University, United Kingdom
Please state any competing interests or state 'None declared': No competing interests

Please leave your comments for the authors below

Physical activity and health related quality of life in former elite and recreational cricketers with upper extremity or lower extremity persistent joint pain

Thank you for submitting your paper "Physical activity and health related quality of life in former elite and recreational cricketers with upper extremity or lower extremity persistent joint pain" to BMJ Open.

Reviewer comment 1.1: In my opinion, the BMJ Open readership with their broad medical research interest may be interested in this paper. The standard of scientific writing was generally strong and clear throughout and there are novel elements in the work. The authors gained ethical approval.
Author response 1.1: The authors would like to thank the reviewer for taking the time to review and improve the overall quality of this manuscript.

Aims/ Background
The authors introduce the health context of their paper by discussing the link between persistent joint pain in former sports participants and OA. They also report that persistent joint pain in recreational and elite former sports participants may reduce physical activity levels and quality of life. The authors make the case that elite sports participants may possess unique psychological characteristics that may have a positive impact on their physical activity levels and quality of life in the presence of persistent pain when compared to recreational sport participants.
Using a sample of former cricketers they evaluate the relationship between persistent joint pain (upper extremity, lower extremity or no persistent pain), physical activity levels and health-related quality of life in former recreational and elite cricketers.

Reviewer comment 1.2: In order to aid the flow of the introduction into the methods it may be helpful to add a sentence explaining why cricketers were the selected sample to address the research questions.
Author response 1.2: The authors would like to thank the reviewer for bringing this to our attention. A sentence has been added outlining the reasoning behind exploring this scientific question in cricketers.
Author action 1.2: On lines 127-132, "The rationale for investigating these relationships in former cricketers was largely due to the high prevalence of upper and lower extremity osteoarthritis and persistent pain amongst former cricketers,12 39 and our previous qualitative work highlighting that a subgroup of former cricketers experience high quality of life and maintain physically active lifestyles despite persistent joint pain."

Reviewer comment 1.3: Typo on page 4, line 121 mental "toughness"
Author response 1.3: The authors would like to thank the reviewer for finding this error. The statement has been revised.

Author action 1.3: On line 121, “Psychological characteristics associated with successful high level athletic performance include resilience, mental toughness and advanced coping skills.”

Methods and analysis

Reviewer comment 1.4: A cross-sectional cohort design was used. Statistical methods used for analyses appear appropriate.

Author response 1.4: The authors would like to thank the reviewer for this comment.

Reviewer comment 1.5: From the participants and recruitment section it is difficult to know if the response rate is low because the vast majority of the sample frame did not match the inclusion criteria (for example many will have been current players) or if this represents some kind of responder bias. This is worthy of discussion in the limitations section and may affect the generalisability of the findings.

Author response 1.5: The authors would like to thank the reviewer for bringing this to our attention. Within the limitations paragraph, a sentence has been added to describe the inability of this methodological design to discern the true questionnaire response rate, and the resultant bias associated with this limitation..

Author action 1.5: On lines 469-473, “Potential participants were informed of the inclusion and exclusion criteria during recruitment, and were able to self-select eligibility to participate. Due to this recruitment strategy, it is not possible to determine the questionnaire response rate, nor responder bias. This recruitment strategy may decrease the generalisability of these findings.”

Reviewer comment 1.6: Was there likely to be any unmeasured confounders that could have affected your relationships of interest? If so this could added to the discussion.

Authors response 1.6: The authors would like to thank the reviewer for this comment. Potential confounders that were not measured include socioeconomic status and participation in other sports. Socioeconomic status can have an effect on quality of life. Further, while the number of seasons played in other sports was captured, we did not control for other sports played, which has shown to improve quality of life. These discrepancies could decrease the generalisability of these findings.

Author action 1.6: On lines 465-469, “There is also the possibility of bias due to unmeasured confounding. Potential confounders that were not measured in this study include socio-economic status and other sports played, which both have been observed to be related to HRQoL.76 94 95 These discrepancies may decrease the generalisability of these findings.”

Reviewer comment 1.7: Is it possible that truncating moderate and vigorous physical activity at 180 minutes could produce a ceiling effect in your analysis given that this is a formerly active sample including elite athletes? Was this truncation applied to many participants?

Author response 1.7: The authors would like to thank the reviewer for this question. Truncating physical activity is the standardized methodological approach suggested by the IPAQ scoring protocol (IPAQ Group, 2005). This is performed in order to minimize the effect of responder physical activity overestimation. While performing the truncation could potentially have a ceiling effect, previous research has shown that physical activity overestimation is a problem, and can greatly influence the results. Please refer to Sallis et al. (2000) “Assessment of physical activity by self-report: status, limitations, and future directions” for further explanation. Due to this, performing physical activity truncation was performed.

In terms of number of participants that had their physical activity truncated, 117 (17%) participants were truncated.

Author action 1.7: On lines 196, “A total of 117 (17%) of participants had PA reports truncated.”

Results

Reviewer comment 1.8: Results were clear. As a journal style point, for consistency, 4 decimal places may not be required for p-values.

Author response 1.8: The authors would like to thank the reviewer for bringing this to our attention. The p-values have been decreased to three decimal places where appropriate.

Discussion and conclusion

Reviewer comment 1.9: To what extent do you think that measurement error and ceiling effect of your truncated IPAQ SF may have affected your findings?

Author response 1.9: The authors would like to thank the reviewer for this question. A total of 117 (17%) % of all participant physical activity measurements were truncated. As stated above in author response 1.7, truncating physical activity is the standardized methodological approach suggested by the IPAQ scoring protocol (IPAQ Group, 2005). This is performed in order to minimize the effect of responder physical activity overestimation. However, despite truncation, daily physical activity levels were still greater than physical activity measures for European men (120 METS per day versus 50 METS per day). These findings demonstrate that there is a distinct difference in physical activity in former cricketers in comparison to the average European male population.

In terms of measurement error, the IPAQ has shown to have strong concurrent and construct validity. Specifically, the IPAQ, in relation to activity monitors, demonstrated a strong relationship with total physical activity and vigorous activity (Hägstromer et al. 2006, The International Physical Activity Questionnaire (IPAQ): a study of concurrent and construct validity). Due to this information and considering our cross-sectional study design, we believe that the IPAQ was an appropriate instrument to measure physical activity

However, in response to your comment, greater clarity has been added to the methods discussion sections.

Author action 1.9: On lines 196, “A total of 117 (17%) of participants had PA reports truncated.”

On lines 450-453, “Physical activity was truncated above 180 minutes, which may introduce a ceiling effect. Further, PA recall may not be as precise as PA monitors, which may introduce measurement error.”

Reviewer comment 1.10: Whilst self-report PA measures may be appropriate for measuring PA at single time points in large samples are there any limitations of self-report measures of PA that could be discussed briefly in the discussion? For example recall bias, social desirability bias and measurement error.

Author response 1.10: The authors would like to thank the reviewer for this comment. Physical activity recall may have limitations. These include physical activity overestimation, and decreased precision in comparison to physical activity monitors. Social desirability may also influence over physical activity estimation. Due to these limitations, statements have been added discussing the potential ceiling effect, measurement error, social desirability, and overall recall bias.

Author action 1.10: On lines 452-455, “Physical activity was truncated above 180 minutes, which may introduce a ceiling effect. Further, PA recall may not be as precise as PA monitors, which may introduce measurement error, and social desirability may cause PA overestimation.”

Reviewer comment 1.11: Page 15 line 390. In my opinion, it is incorrect to say that low levels of missing data “allowed for low bias in the findings” with complete case analysis. I would consider softening this sentence to talk about “risk of bias”. Reasons for missingness as well as the amount of missing data affect risk of bias.

Author response 1.11: Thank you for bringing this to our attention. The statement has been revised in order to focus more on risk of bias and tone down the language.

Author action 1.11: On line 452, “Missing data was low, decreasing the risk of bias in a complete case analysis.

Reviewer comment 1.12: Extra emphasis could be offered on why your findings are of clinical importance.

Author response 1.12: The authors would like to thank the reviewer for this suggestion. Greater emphasis has been added regarding the clinical importance of these findings within the conclusion.
Author action 1.12: On line 484-487, “The high average physical activity levels amongst former cricketers with persistent joint pain could explain the high MCS scores in this group. In contrast, former cricketers with joint pain who are physically inactive may benefit from targeted strategies to increase activity levels, with potential to positively impact QOL.”

Reviewer: 2
Reviewer Name: Takeshi Toyooka
Institution and Country: Nishikawa orthopaedic Clinic
Please state any competing interests or state ‘None declared’: None declared

Please leave your comments for the authors below

Reviewer comment 2.1: This article is a very interesting study. There is some points to be worried about how to write statistics.
Author response 2.1: The authors would like to thank the reviewer for their due diligence in editing this manuscript.

Reviewer comment 2.2: Is the post hoc test results in Table 2 or figure 2? If so, the p-value should be clearly shown in a table or figure where and where compared not only effect size.
Author response 2.2: The authors would like to thank the reviewer for this question. The post hoc results are written in the results paragraph, with the visual representation of the physical activity data displayed in figure 2. Table 2 results are for the next paragraph (The relationship between persistent joint pain, physical activity and health related quality of life) This has been clarified for ease of reading.
Author action 2.2: On line 292, “For a visual representation of the PA data, please refer to Figure 2.”

Reviewer comment 2.3: Additionally, since the unit of the numerical value is described as METS on the line 286 on page 10, isn't this number an effect size?
Author response 2.3: The authors would like to thank the reviewer for this comment. The unit is number of METS difference between upper or lower extremity persistent pain groups and former cricketers without persistent pain. In order to provide further clarity, a statement has been added at the beginning of each results section on the type of statistical analyses performed.
Author Action 2.3: On lines 292-293, “Kruskall Wallis test indicated that there was no difference in physical activity levels between former cricketers with upper extremity persistent joint pain (Median (IQR): 2560 (722-4398) METS), lower extremity persistent joint pain (2215 (527-3903) METS), or no persistent joint pain (2449 (695-4203) METS) (Chi-square: 1.91, p=0.39).”

On line 310-312, “Multivariable linear regressions found that persistent joint pain was not related to physical activity levels (Upper Extremity: Adjusted effect (95% CI): 28.1 METS (-1.1 to 135.7); Lower Extremity: 4.6 METS (-7.5 to 49.4)), see Table 2.”

On lines 341-342, “Multivariable linear regressions found that findings were similar between recreational and elite cricketer subgroups (Table 3).”

Reviewer comment 2.4: Line 275 on page 10, the result of the difference is presented. Is this also the result of post hoc test?
Author response 2.4: The authors would like to thank the reviewer for bringing this to our attention. These results are also the results of a post hoc test. This has been further clarified in the results.

Author action 2.4: On lines 296-300, “There were significant PCS differences between pain groups (p<0.001). Post hoc tests demonstrated that former cricketers reported worse PCS scores if they had persistent joint pain in the upper extremity (PCS: 49.8 (44.9-54.8)) or lower extremity (PCS: 46.7 (41.0-51.9)), compared to former cricketers with no persistent joint pain (PCS: 54.2 (51.5-56.9); Chi-square:121.2, p<0.001).”

Reviewer comment 2.5: Line 286 on page 10, although you show us " Lower Extremity: 20.3 METS" in the main body, the number is shown as Upper extremity persistent joint pain on table 2. which is correct result?
Author response 2.5: The authors would like to thank the reviewer for bringing this to our attention. This was a mistake that has been corrected. The true adjusted effect for the lower extremity was 4.6 METS.
Author action 2.5: On lines 308-310, “Multivariable linear regressions revealed that persistent joint pain was not related to physical activity levels (Upper Extremity: Adjusted effect (95% CI): 28.1 METS (-1.1 to 135.7); Lower Extremity: 4.6 METS (-7.5 to 49.4)), see Table 2.”

Reviewer comment 2.6: The word "similar" "associate" should not be used as a result of ANOVA. Finally, did you use two way ANOVA to analyze table 3? It is easier to understand if the statistics used to describe each result are described.
Author response 2.6: The authors would like to thank the reviewer for this suggestion. Multivariable linear regressions were performed for the subgroup analysis, an ANOVA was not performed. The purpose of this study was to evaluate the relationship between persistent joint pain (upper extremity, lower extremity, or no persistent joint pain), PA levels and HRQoL in former cricketers; and then compare these relationships in former elite and recreational cricketer subgroups. Understanding the relationship between standard-of-play (elite and recreational cricketers), PA, and HRQoL was not a direct comparison between elite and recreational cricketers, Due to these methodological strategy, in line with the aims of the study, the words similar and associated were used. However, to increase clarity, the overall statistical method employed for each result section has been added to the beginning of each results section.
Author action 2.6:
On lines 292-293, “Kruskall Wallis test indicated that there was no difference in physical activity levels between former cricketers with upper extremity persistent joint pain (Median (IQR): 2560 (722-4398) METS), lower extremity persistent joint pain (2215 (527-3903) METS), or no persistent joint pain (2449 (695-4203) METS) (Chi-square: 1.91, p=0.39).”

On line 310-312, “Multivariable linear regressions found that persistent joint pain was not related to physical activity levels (Upper Extremity: Adjusted effect (95% CI): 28.1 METS (-1.1 to 135.7); Lower Extremity: 4.6 METS (-7.5 to 49.4)), see Table 2.”

On lines 341-342, “Multivariable linear regressions found that findings were similar between recreational and elite cricketer subgroups (Table 3).”

VERSION 2 – REVIEW

**REVIEWER**	Dr Jonathan Quicke<br>Keele University<br>United Kingdom
**REVIEW RETURNED**	26-Sep-2019

**GENERAL COMMENTS**	I am satisfied that the authors have addressed all of my review comments and questions. One final formatting consideration on my marked copy: in the reference list, the formatting for reference

	number 67 (Filbay et al 2019) looks incomplete and inconsistent with the other references.

**REVIEWER**	Takeshi Toyooka<br>Nishikawa orthopaedic clinic, JAPAN
**REVIEW RETURNED**	11-Sep-2019

**GENERAL COMMENTS**	Thank you for resubmitting your answer. The results have become easier to understand thank you to the author's corrections.

VERSION 2 – AUTHOR RESPONSE

Reviewer: 2

Reviewer Name: Takeshi Toyooka

Institution and Country: Nishikawa orthopaedic clinic, JAPAN

Please state any competing interests or state 'None declared': None declared

Please leave your comments for the authors below

Reviewer comment 2.1: Thank you for resubmitting your answer. The results have become easier to understand thank you to the author's corrections.

Author response 2.1: The authors would like to thank the reviewer for their work on this manuscript.

Reviewer: 1

Reviewer Name: Dr Jonathan Quicke

Institution and Country:

Keele University

United Kingdom

Please state any competing interests or state 'None declared': None declared

Please leave your comments for the authors below

Reviewer comment 3.1: I am satisfied that the authors have addressed all of my review comments and questions. One final formatting consideration on my marked copy: in the reference list, the formatting for reference number 67 (Filbay et al 2019) looks incomplete and inconsistent with the other references.

Author response 3.1: The reference in question has been revised in terms of spacing and further clarity. This reference is in press with the Journal of Sports Medicine and will be published shortly.

Author action 3.1: 76. FIlbay SR PT, Thomas B, McKay C, Adams J, Arden NK. Quality of life and life satisfaction in former athletes: a systematic review and meta-analysis. Sports Medicine 2019. In Press.
